# Shifts in cranial integration associated with ecological specialization in pinnipeds (Mammalia, Carnivora)

**DOI:** 10.1098/rsos.190201

**Published:** 2019-03-27

**Authors:** Marcela Randau, Daniela Sanfelice, Anjali Goswami

**Affiliations:** 1 The Natural History Museum, Life Sciences, London, UK; 2 Instituto Federal do Rio Grande do Sul, Campus Restinga, Porto Alegre, Brazil

**Keywords:** modularity, morphological evolution, shape, morphometrics, disparity, ecological transitions

## Abstract

Patterns of trait integration reflect the underlying genetic and developmental architecture of morphology and significantly influence the direction of evolution. Nevertheless, the relationship between integration and disparity is complex and unlikely to be uniform across large phylogenetic and ecological scales. To date, there are little data comparing patterns of integration across major ecological transitions, limiting understanding of the processes driving changes in trait integration and their consequences. Here, we investigated patterns of cranial integration and disparity across pinnipeds, three closely related carnivoran families that have undergone a secondary adaptation to the aquatic niche with varying levels of ecological differentiation. With a three-dimensional geometric morphometric dataset of 677 specimens spanning 15 species, we compared five models of trait integration, and examined the effects of sexual dimorphism and allometry on model support. Pinnipeds varied greatly in patterns of cranial integration compared to terrestrial carnivorans. Interestingly, this variation is concentrated in phocids, which may reflect the broader range of ecological and life-history specializations across phocid species, and greater independence from the terrestrial habitat observed in that group, relative to otariids. Overall, these results indicate that major ecological transitions, and presumably large changes in selection pressures, may drive changes in phenotypic trait integration.

## 1. Background

Analyses of phenotypic integration measure the magnitude to which traits are correlated and therefore dependent, whether due to genetic, developmental or functional interactions [1]. Sets of traits showing high integration among themselves, but lower integration with traits outside of the set, can be termed modules [1,2]. Within a highly integrated module, variation of a trait is dependent on congruent variation on all other traits within the same module, and therefore, high integration has been hypothesized to greatly shape trait evolution [3–6]. Nevertheless, the relationship between integration and trait variance or, on a macroevolutionary scale, disparity, is complex, and high trait integration has been shown to both constrain and promote trait variation [7–14]. Ultimately, the effect of integration among traits may be dependent on whether the major axis of shape covariation aligns with the direction of selection on those traits [15,16]. In short, whereas high integration among traits forces most variation to happen along few dimensions (and therefore it constrains variation on other directions), if the direction of the selection vector coincides with the main axis of shape variation high integration may promote high variance along that dimension [15,16]. The opposite is expected to happen when there is a significant difference in direction between the selection and integration vectors, and in this case, high trait correlation may constrain increases in shape variance by preventing the exploration of certain trajectories and morphospace regions [9,17–19]. Identifying which, if either, of these effects has dominated through organismal evolution is thus a central question in biology.

The mammalian skull has been the focus of evolutionary questions focusing on trait integration and its relationship to disparification (i.e. increase in morphological variance) and ecological specialization in an extensive number of studies (e.g. [4,6,20–35]). Interspecific comparisons of shape evolution among mammals are facilitated by a relatively high conservation of morphological characters and studies of developmental and genetic skull patterning, alongside a rich literature on species' ecology (e.g. [6,22,23,27,29,32,36–43]). Within this literature, a general mammalian, or more specifically, therian model of skull trait correlation has been previously suggested, with demonstration of a six-module pattern conserved across large phylogenetic sampling [24,27,44,45]. Nevertheless, analyses of whether and how this conserved pattern has been modified within selected groups that have undergone major ecological shifts are still comparatively rare (e.g. [46]). A particular issue in this regard is that a considerable proportion of the existing literature relies on confirmatory analysis of a single hypothesis of modular organization, rather than comparisons of alternative hypotheses (see [44] for a thorough discussion). Comparisons of any deviations from the general pattern of cranial organization observed in most therian mammals are key to understanding how trait integration itself can evolve and affect morphological evolution.

**Figure 1.**
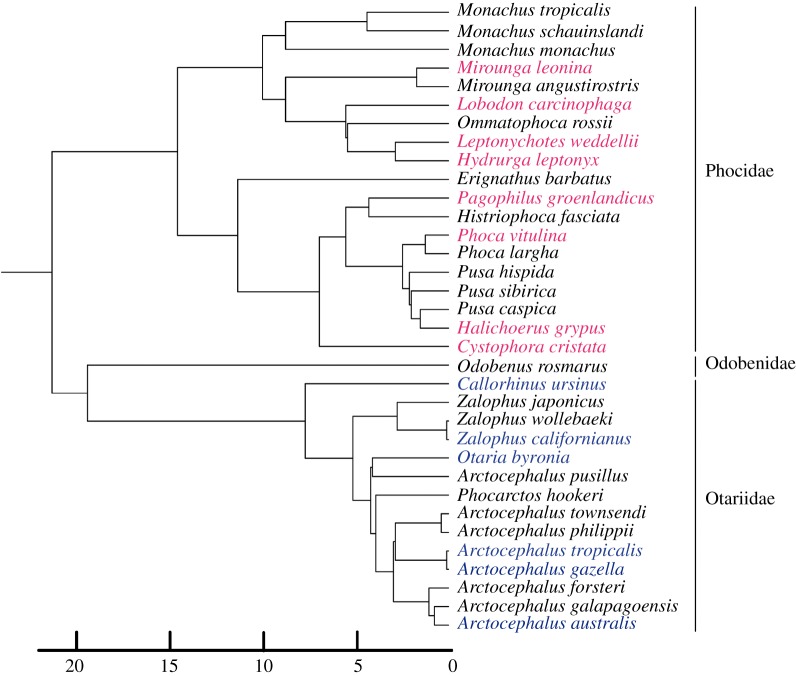
Illustrative phylogenetic relationships between the three pinniped families (Phocidae, Odobenidae and Otariidae) with the mean divergence times estimates. The relationships depicted here are based on [54]. Species included in this study are depicted in bold and coloured (Phocidae: pink; Odobenidae: black; Otariidae: blue).

Pinnipeds are carnivoran mammals that have undergone a secondary adaptation to the aquatic environment [47,48]. Whereas extant species fall within three families: Phocidae (true seals), Otaridae (fur seals and sea lions) and Odobenidae (consisting of only one extant species, the walrus), phylogenetic relationships within Carnivora demonstrate that pinnipeds are arctoid caniforms closely related to musteloids. The reconstructed relationships among the three families vary depending on whether molecular or morphological datasets are used, with molecular analyses indicating a closer association between otariids and odobenids, forming the Otarioidea, and morphological data supporting the Phocomorpha (odobenids with phocids) [48–51]. Although the first stem pinniped fossils date from *ca* 28 million years ago (hereafter, ma), molecular phylogenies support an earlier split between Otarioidea (i.e. odobenids + otariids) and Phocidae at around 33 ma [48,52,53]. Extant Otariidae is thought to represent a relatively recent radiation, with the first unambiguous crown otariid fossils dating from the Late Pliocene [50,53]. By contrast, extant phocids began to diverge earlier, with a split between the two major extant clades, monachines and phocines, at around 22 ma [48,53,54] (figure 1).

The phylogenetic relationships of pinnipeds, nested within a diverse extant terrestrial clade, and the similarities in cranial morphology with their terrestrial relatives, despite their adaptations for life in water, make this group a unique model for studies of morphological evolution, including integration. Specifically, there is behavioural and life-history variation across the three families, including the time and activities performed on land or in water [47]. Although most species are generalist feeders, differences in ecological and life-history traits such as mating strategies and duration of parental care are markedly different across pinnipeds. While odobenids have lactation times as long as 3 years, otariids display external sexual dimorphism with large harems and long pup weaning time. Phocids, however, show a greater diversity of mating strategies and shape differences between sexes with relatively precocial pups, and overall spend less time on land [55,56]. Further, the above-mentioned earlier divergence within Phocidae may relate to the higher degree of ecological diversification observed in extant phocids when compared with otariids. Whereas otariids are generally more similar in skull morphology and diet, a much broader range of dietary specializations and mating displays is observed in extant phocids, as well as an increased diving depth in some species (e.g. the Southern elephant seal, *M. leonina*) and greater independence from the terrestrial environment, which is reflected in greater disparity of phocid skull shape [40,47,48,53,57,58].

Here, we investigate skull modularity and variation in 15 species of pinnipeds across the three families by assessing five alternative hypotheses of modularity based on tissue origination, ossification mode, function, and the previously suggested hypotheses of skull modularity of either two or six partitions (see Material and methods). Further, for the best-supported model, we test if within-module magnitude of integration correlates with levels of morphological disparity per individual species. Finally, we consider our results in relation to variable degrees of independence from the terrestrial realm and ecological specialization, and also compare our results to previously published data on terrestrial carnivorans (fissipeds) to place our results in the context of how ecological shifts may alter evolutionary patterns that are otherwise conserved across large clades and time periods.

## 2. Material and methods

**Figure 2.**
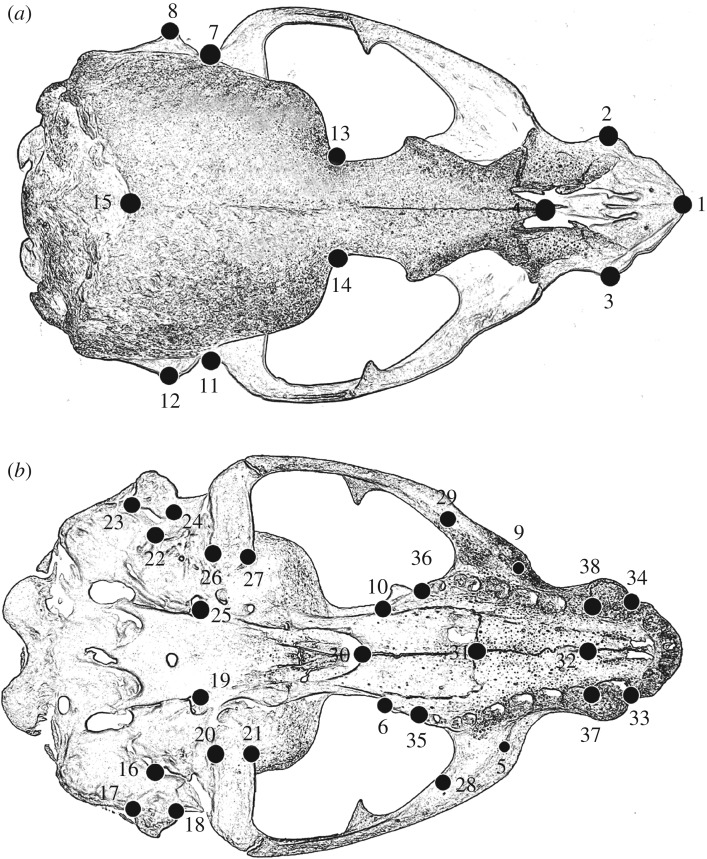
Model representation of landmark distribution across a general pinniped skull. (*a*) dorsal view; (*b*) ventral view.

Skull morphology was characterized with 38 type I and II three-dimensional landmarks across the skull (figure 2 and table 1) in 677 pinniped specimens. Landmarks were digitized using an Immersion Microscribe G2X (Solution Technologies, Inc., Oella, MD, USA). This dataset was composed of 35 walruses (*Odobenus rosmarus*, Odobenidae), 233 otariids across six species (Otariidae) and 409 specimens across eight phocid species (Phocidae) (electronic supplementary material, table S1 for museum specimen numbers). All specimens were of adult individuals, and an effort was made to digitize similar numbers of male and female specimens per species whenever possible (i.e. sex information was recorded when available, however 111 out of the 677 available specimens were unidentified with regard to sex, table 2 and electronic supplementary material, table S1). In order to minimize user measurement error, all specimens were digitized by the same person (D.S.). Specimen numbers varied from 25 specimens for the northern fur seal (*Callorhinus ursinus*, Otariidae) to 70 for the Weddell seal (*Leptonychotes weddellii*, Phocidae), with a mean of 45 specimens per species (table 2)*.* The datasets supporting this article have been uploaded as part of the electronic supplementary material.

**Table 1.** Description of the 38 landmarks comprising the dataset analysed here.

landmark	definition
1	most anterior point of the inter-premaxillae suture
2	most lateral point (extreme) in the canine alveolus near the ventral border
3	most lateral point (extreme) in the canine alveolus near the ventral border
4	posterior limit of the inter-nasal suture
5	most posterior–lateral point of the anterior aperture for the infraorbital canal (left side)
6	most posterior point of the alveolar process of maxilla in the lateral view (left side)
7	most dorsal point of the external acoustic meatus roof (left side)
8	most dorsal point of the mastoid process (left side)
9	most posterior–lateral point of the anterior aperture for the infraorbital canal (right side)
10	most posterior point of the alveolar process of maxilla in the lateral view (right side)
11	most dorsal point of the external acoustic meatus roof (right side)
12	most dorsal point of the mastoid process (right side)
13	most posterior point of the postorbital constriction
14	most posterior point of the postorbital constriction
15	most dorsal point of the dorsal nuchal crest (or the anterior extremity of the supraoccipital)
16	most posterior point of the facial foramen (left side)
17	ventral limit of the posterior mastoid crest (left side)
18	ventral limit of the anterior mastoid crest (left side)
19	most anterior point of the anterior foramen for the carotid canal (left side)
20	medial limit of the retroglenoid process (left side)
21	anterior–medial corner of the glenoid fossa (left side)
22	most anterior point of the anterior foramen for the carotid canal (right side)
23	ventral limit of the posterior mastoid crest (right side)
24	ventral limit of the anterior mastoid crest (right side)
25	most posterior point of the facial foramen (right side)
26	medial limit of the retroglenoid process (right side)
27	anterior–medial corner of the glenoid fossa (right side)
28	posterior limit for the dorsal border of the zygomatic process of the maxilla
29	posterior limit for the dorsal border of the zygomatic process of the maxilla
30	most posterior point of the inter-palatine suture
31	most posterior point of the inter-maxillae suture
32	most posterior point of the inter-premaxilla suture
33	most anterior point of the premaxilla-maxilla suture
34	most anterior point of the premaxilla-maxilla suture
35	posterior limit for the last post-canine alveolus
36	posterior limit for the last post-canine alveolus
37	anterior limit for the ‘first’ post-canine alveolus present
38	anterior limit for the ‘first’ post-canine alveolus present

**Table 2.** Summary of specimen numbers per species and family within Pinnipedia included in the analyses presented here.

family	number of specimens	female	male	unidentified
Odobenidae				
*Odobenus rosmarus*	35	14	20	1
number of specimens per family	35			
sex-identified specimens	34			
Otariidae				
*Arctocephalus australis*	42	13	29	0
*Arctocephalus gazella*	47	22	25	0
*Arctocephalus tropicalis*	29	14	15	0
*Callorhinus ursinus*	25	12	13	0
*Otaria byronia*	48	30	18	0
*Zalophus californianus*	42	17	25	0
number of specimens per family	233			
sex-identified specimens	233			
Phocidae				
*Cystophora cristata*	50	17	22	11
*Halichoerus grypus*	50	24	20	6
*Hydrurga leptonyx*	57	21	20	16
*Lobodon carcinophaga*	60	20	25	15
*Leptonychotes weddellii*	70	22	20	28
*Mirounga leonina*	30	12	18	0
*Pagophilus groenlandicus*	47	11	19	17
*Phoca vitulina*	45	14	14	17
number of specimens per family	409			
sex-identified specimens	299			
total number of specimens	677			
total number of sex-identified specimens	566			

### 2.1. Data analyses

All analyses were performed in R v. 3.5.0, using packages ‘geomorph’ v. 3.0.6 [59] and ‘EMMLi’ v. 0.0.3 [44].

### 2.2. Pinniped skull shape variation

First, all specimens were subjected to a general Procrustes superimposition in order to remove all non-shape information (i.e. rotation, translation and scale), and centroid size per specimen was recorded as a proxy for specimen size. A principal component analysis (PCA) was performed on the Procrustes coordinates of the 677 pinniped specimens to visualize the occupation of the morphospace of the sampled species. Differential occupation of the morphospace by each of the pinniped families with a similar landmark dataset has been explored in detail in previous publications [52,53], and therefore, here, we focus only on the results in the first two main PC axes (see below).

### 2.3. Dimorphism and allometry

Prior to all subsequent analyses, raw specimen data were subjected to separate species-specific general Procrustes superimpositions. This was performed in order to account for different developmental or size factors that may be singular to each species within the pinniped families.

In order to test if size-related shape variation (allometry) or sexual dimorphism influence patterns of modularity, we performed regressions of the shape variables on centroid size while specifying sex as the groups to be considered (i.e. shape ∼ size + sex). This analysis was necessarily limited to specimens with sex information available (table 2). For each species, if males and females differed significantly in the estimated influence of allometry on skull shape (*p* < 0.05), a homogeneity of slopes test was performed. This test was performed with the function ‘procD.allometry’ in the geomorph package in R. If results supported a significant influence of size or size + sex on skull shape, we re-analysed the hypotheses of modularity (see below) on the shape residuals from the relevant significant regression (i.e. either allometry-corrected across the entire species sample if males and females displayed the same allometric trajectory or separately corrected for allometric shape changes on each sex per species).

### 2.4. Cranial modularity

**Figure 3.**
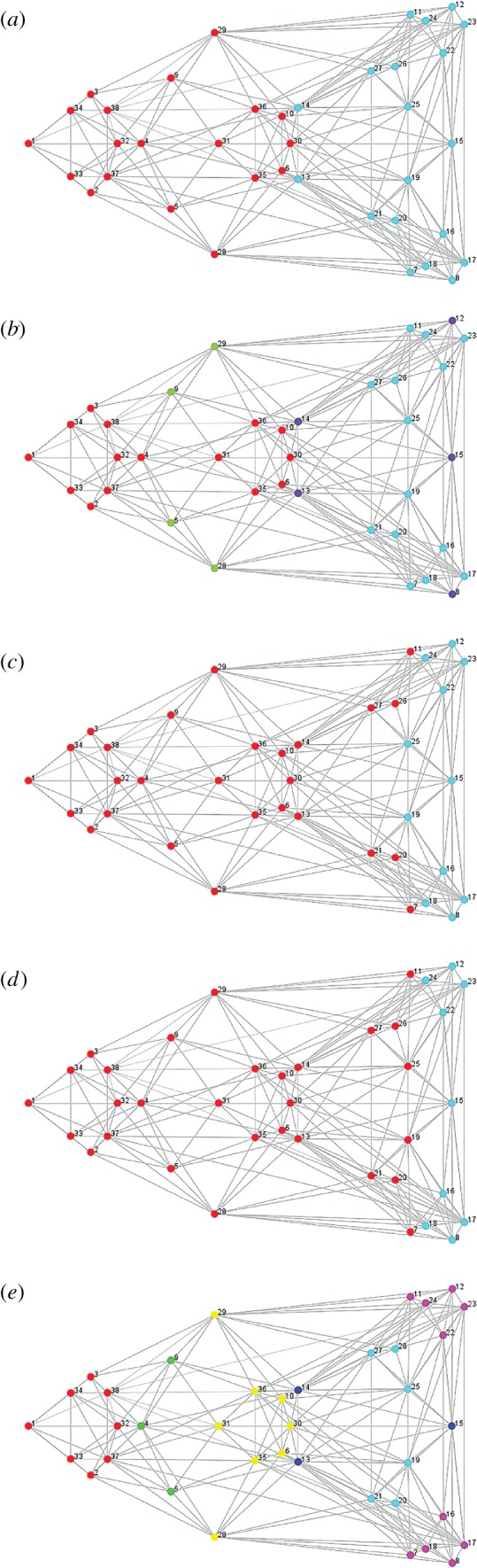
Hypothesized modularity models with numbers of modules varying from two to six. Models: (*a*) face and neurocranium model; (*b*) functional groups model (four modules: oral–nasal, orbital, vault and basicranial); (*c*) tissue origin (two modules: neural crest and paraxial mesoderm); (*d*) ossification mode model (two modules: dermal and endochondral) and (*e*) the six-cluster model (six modules: oral, nasal–orbital, molar–zygomatic, vault, pterygoid and basicranial regions).

Five alternative hypotheses of cranial modularity were defined by assigning landmarks to hypothesized modules, which ranged from two to six modules per model (figure 3 and table 3): (i) two-module model: face and neurocranium [60]; (ii) functional groups model (four modules: oral–nasal, orbital, vault and basicranial); (iii) tissue origin model (two modules: neural crest and paraxial mesoderm); (iv) ossification mode model (two modules: dermal and endochondral); and (v) six-module model (six modules: oral, nasal–orbital, molar–zygomatic, vault, pterygoid and basicranial regions; modified from [27]). Support for these alternative hypotheses was compared individually for each species using EMMLi (Evaluating Modularity with Maximum Likelihood) [44], a maximum-likelihood-based approach that factors in model parametrization and comparison of model likelihoods with a sample-corrected Akaike information criterion. As part of its output, EMMLi supplies the posterior probability for each of the models tested, allowing for direct comparison of the preferred model to all other available models and their parameters. Further, EMMLi returns both intra- and inter-module magnitudes of correlation (i.e. the levels of integration both within and between modules, see below). In order to test the hypotheses of modularity for each of the species included here, congruence coefficients were calculated on the species-specific Procrustes coordinates, as implemented in EMMLi.

**Table 3.** Schematic distribution of landmarks per each of the five modularity models tested here.

landmark	model 1: face and neurocranium	model 2: functional groups	model 3: tissue origin	model 4: ossification mode	model 5: six-cluster
1	1	1	1	1	1
2	1	1	1	1	1
3	1	1	1	1	1
4	1	1	1	1	2
5	1	2	1	1	2
6	1	1	1	1	3
7	2	4	1	1	6
8	2	3	2	2	6
9	1	2	1	1	2
10	1	1	1	1	3
11	2	4	1	1	6
12	2	3	2	2	6
13	2	3	1	1	4
14	2	3	1	1	4
15	2	3	2	2	4
16	2	4	2	2	6
17	2	4	2	2	6
18	2	4	2	2	6
19	2	4	2	1	5
20	2	4	1	1	5
21	2	4	1	1	5
22	2	4	2	1	5
23	2	4	2	2	6
24	2	4	2	2	6
25	2	4	2	2	6
26	2	4	1	1	5
27	2	4	1	1	5
28	1	2	1	1	3
29	1	2	1	1	3
30	1	1	1	1	3
31	1	1	1	1	3
32	1	1	1	1	1
33	1	1	1	1	1
34	1	1	1	1	1
35	1	1	1	1	3
36	1	1	1	1	3
37	1	1	1	1	1
38	1	1	1	1	1

Confirmation of the best-supported model of modularity from EMMLi analysis was conducted using covariance ratio (CR) analysis [61]. Significance levels for CR results were obtained by random assignation of landmarks to 5000 alternative models of modularity, and tested with a *p* < 0.05 threshold.

### 2.5. Integration and disparity

In addition to estimating magnitude of within-module integration with the *ρ* values output by EMMLi for the best-supported model of modularity (see above), magnitudes of integration for each module were calculated with a widely applied method, the relative eigenvalue standard deviation analysis (i.e. eigenvalue dispersion) [62]. With high levels of shape integration, variance concentrates in the first few eigenvectors due to high covariance between shape traits, and this is reflected in high levels of eigenvalue dispersion [7,16,62].

Module disparities were calculated as maximum Procrustes distance between specimens on each species-specific dataset [63]. Due to the differences in landmark number across modules within models, total module morphological disparity was scaled by landmark number for each module before comparing across modules.

The magnitude of integration for each module was compared to the species' mean and median integration levels across modules within the selected model. Modules were considered to be more integrated if they displayed integration magnitudes that were higher than the mean and median values for each species across modules. The same comparison was conducted for magnitude of disparity across species. Results were then compared to investigate whether the most disparate modules were the ones with either the highest or lowest magnitudes of integration.

These analyses of integration and disparity were performed on the shape data prior to allometric size-correction (i.e. on the coordinate data after Procrustes superimposition per species, which corrects for shape variation related to isometric size, but not allometry). The decision to do so was based on the empirical and theoretical observations that size can be one of the major drivers of morphological integration and one which has an overall effect across the whole structure [2,64,65]. Because size-related shape change (allometry) affects most, if not all cranial regions, it is expected to impose integration across the entire structure. This effect is likely to obscure more subtle or localized patterns from developmental or functional interactions of traits. Therefore, comparing analyses of modularity conducted with and without correction for allometry (as was performed here) allows for isolating potential drivers of covariation (e.g. allometry, function or development) [66,67]. However, from the perspective of how integration influences disparity, it is by modulating the ability to respond to selection [15,16], for which the total amount of integration is the most informative aspect, regardless of the source of that integration, and so allometry was not removed for the comparison of total integration and disparity.

## 3. Results

### 3.1. Pinniped skull shape variation

**Figure 4.**
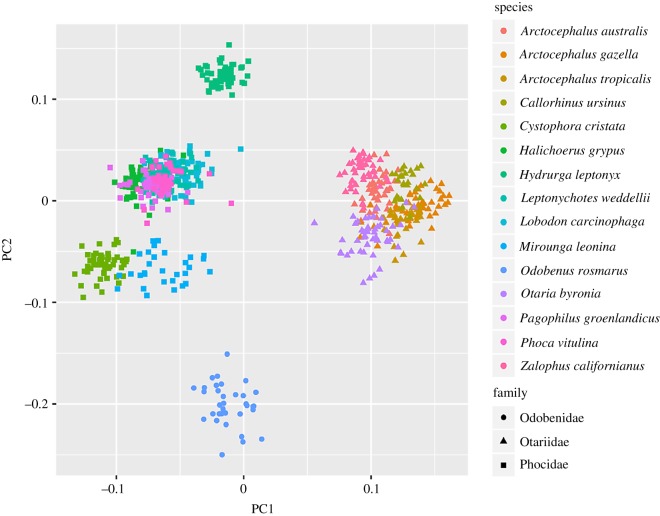
Results from the PCA displaying distribution of specimens across the PC1 (33.5% of variation) × PC2 (20% of variation) morphospace. Whereas familial identity of specimens is marked by symbols (i.e. circles for odobenids, triangles for otariids and squares for phocids), species are differentiated by the colour scheme on the right.

Principal components 1 and 2 explained *ca* 54% of total shape variation. All other PCs explained a relatively small percentage of total variation, with PCs 3 and 4 explaining around 8% and 5%, respectively, and all other PCs summarizing less than 5% each (table 4). The three pinniped families occupied distinct regions of morphospace on the first two principal component axes (figure 4). Otariid species cluster on the positive extreme of PC1, but phocids are more dispersed across the two major axes, with three main phocid groupings: a *Hydrurga leptonyx* cluster with the most positive PC2 scores, an intermediate *L. weddellii* + *Pagophilus groenlandicus* + *Halichoerus grypus* + *Phoca vitulina* + *Lobodon carcinophaga* cluster, and a *Cystophora cristata* + *Mirounga leonina* cluster on the negative end of the phocid PC2 distribution, towards the walrus morphospace.

**Table 4.** Summary of results from the PCA for the first 35 PCs (95.1% of total variance). Subsequent PCs explained either 0.2% or less of total shape variance.

	standard deviation	proportion of variance	cumulative proportion
PC1	0.086	0.335	0.335
PC2	0.066	0.200	0.535
PC3	0.042	0.082	0.616
PC4	0.035	0.054	0.671
PC5	0.031	0.043	0.714
PC6	0.027	0.034	0.747
PC7	0.023	0.025	0.772
PC8	0.022	0.023	0.795
PC9	0.020	0.018	0.812
PC10	0.019	0.016	0.828
PC11	0.016	0.012	0.840
PC12	0.015	0.010	0.851
PC13	0.014	0.009	0.860
PC14	0.013	0.008	0.868
PC15	0.013	0.008	0.875
PC16	0.012	0.007	0.882
PC17	0.012	0.006	0.888
PC18	0.011	0.006	0.894
PC19	0.011	0.005	0.899
PC20	0.010	0.005	0.904
PC21	0.010	0.005	0.909
PC22	0.010	0.004	0.913
PC23	0.009	0.004	0.917
PC24	0.009	0.004	0.921
PC25	0.009	0.004	0.924
PC26	0.009	0.003	0.927
PC27	0.008	0.003	0.931
PC28	0.008	0.003	0.934
PC29	0.008	0.003	0.936
PC30	0.008	0.003	0.939
PC31	0.008	0.003	0.942
PC32	0.007	0.003	0.944
PC33	0.007	0.002	0.947
PC34	0.007	0.002	0.949
PC35	0.007	0.002	0.951

### 3.2. Dimorphism and allometry

#### 3.2.1. Odobenidae

Analyses of shape and allometric size dimorphism showed that whereas there was a significant amount of shape change that was driven by changes in specimen allometric size (*R*^2^ = 0.13, *p* = 0.0002; table 7), there was no difference between the amount of size-related shape changes between males and females (i.e. after size-correction, *p* ≫ 0.05, table 5).

**Table 5.** Results from the Procrustes regressions of the shape variables on centroid size while specifying sex as the groups to be considered (i.e. shape ∼ size + sex). Significant results for the sex variable are bold, whereas significant results for the homogeneity of slopes (i.e. displaying different allometric shape trajectories per sex) are bold and in bold italics.

species	variables	*R*^2^	*p*-value
Odobenidae			
*Odobenus rosmarus*	Log(Csize)	0.132	0.000
	sex	0.028	0.360
Otariidae			
*Arctocephalus gazella*	Log(Csize)	0.167	0.000
	sex	0.014	0.089
*Arctocephalus australis*	Log(Csize)	0.114	0.000
	sex	0.047	**0.000**
	homogeneity of slopes	0.026	0.165
*Arctocephalus tropicalis*	Log(Csize)	0.272	0.000
	sex	0.030	**0.026**
	homogeneity of slopes	0.021	0.545
*Callorhinus ursinus*	Log(Csize)	0.341	0.000
	sex	0.030	0.302
*Otaria byronia*	Log(Csize)	0.273	0.002
	sex	0.154	**0.020**
	homogeneity of slopes	0.026	***0.025***
*Zalophus californianus*	Log(Csize)	0.111	0.000
	sex	0.037	**0.023**
	homogeneity of slopes	0.016	0.795
Phocidae			
*Cystophora cristata*	Log(Csize)	0.236	0.000
	sex	0.033	**0.024**
	homogeneity of slopes	0.025	0.205
*Halichoerus grypus*	Log(Csize)	0.134	0.000
	sex	0.058	**0.033**
	homogeneity of slopes	0.083	***0.025***
*Hydrurga leptonyx*	Log(Csize)	0.045	0.009
	sex	0.076	**0.035**
	homogeneity of slopes	0.053	0.245
*Leptonychotes weddellii*	Log(Csize)	0.051	0.005
	sex	0.042	0.636
*Lobodon carcinophaga*	Log(Csize)	0.052	0.001
	sex	0.043	0.470
*Mirounga leonina*	Log(Csize)	0.205	0.000
	sex	0.037	0.123
*Pagophilus groenlandicus*	Log(Csize)	0.073	0.001
	sex	0.027	0.700
*Phoca vitulina*	Log(Csize)	0.065	0.128
	sex	0.069	**0.040**
	Log(Csize) : sex	0.099	0.010
	homogeneity of slopes	0.099	***0.015***

#### 3.2.2. Otariidae

Among the six otariid species included here, the only species for which there was an allometric trajectory that was significantly different between sexes was *Otaria byronia* (table 5). For all other otariid species, allometric correction was performed at the species level.

#### 3.2.3. Phocidae

Allometric size-correction was performed at the species level for six of the eight species (*C. cristata*, *H. leptonyx*, *L. weddellii*, *Lo. carcinophaga*, *M. leonina* and *Pa. groenlandicus*; table 5). The size + sex regression analyses for the grey seal (*Ha. grypus*) and harbour seal (*Ph. vitulina*) demonstrated that males and females within these species present different allometric trajectories for changes in skull shape (table 5, homogeneity of slopes test *p* < 0.05). Whereas a separate correction was performed for males and females of the grey seal, only the male skull shape of the harbour seal displayed a significant relationship with size, and therefore, only the male shape was corrected (i.e. a separate modularity model test was performed on the uncorrected shape coordinates of female harbour seals).

### 3.3. Cranial modularity

#### 3.3.1. Odobenidae

Prior to any correction, the best-supported model for the walrus skull modularity was model ‘1’ (i.e. face and neurocranium), supporting a two-module organization of skull traits (table 6; electronic supplementary material, table S2). This result was confirmed with a CR value of 0.8 (*p* = 0.0002, table 7).

**Table 6.** Summary of the best-supported model of modularity selected with EMMLi analysis per species and their respective posterior probabilities. Abbreviations for the model parametrization are as follows: whereas ‘sep’ and ‘same’ stand for separate and same integration levels, respectively, ‘within’ and ‘between’ refer to integration levels within and between modules.

	species	best-supported model	posterior probability
Odobenidae	*Odobenus rosmarus*	face versus neurocranium.sep.within + same.between	0.469
Otariidae	*Arctocephalus australis*	6-clusters.sep.within + same.between	0.896
	*Arctocephalus gazella*	6-clusters.sep.within + sep.between	1.000
	*Arctocephalus tropicalis*	6-clusters.sep.within + sep.between	1.000
	*Callorhinus ursinus*	6-clusters.sep.within + sep.between	0.993
	*Otaria byronia*	6-clusters.sep.within + sep.between	0.844
	*Zalophus californianus*	6-clusters.sep.within + sep.between	0.435
Phocidae	*Cystophora cristata*	face versus neurocranium.same.within + same.between	0.356
	*Halichoerus grypus*	6-clusters.sep.within + sep.between	0.986
	*Hydrurga leptonyx*	face versus neurocranium.same.within + same.between	0.548
	*Lobodon carcinophaga*	face versus neurocranium.same.within + same.between	0.474
	*Leptonychotes weddellii*	6-clusters.sep.within + sep.between	0.995
	*Mirounga leonina*	function.sep.within + same.between	0.949
	*Pagophilus groenlandicus*	6-clusters.sep.within + same.between	0.367
	*Phoca vitulina*	face versus neurocranium.sep.within + same.between	0.569

**Table 7.** Summary of results from the CR analysis of each of the modularity models selected from the EMMLi tests both prior and after corrections. Abbreviations for the model parametrization are as follows: whereas ‘sep’ and ‘same’ stand for separate and same integration levels, respectively, ‘within’ and ‘between’ refer to integration levels within and between modules. Abbreviations in the ‘Sample’ column are as follows: ‘W’ for whole species sample; ‘F’ for the female specimens of individual species; ‘M’ for the male specimens of individual species.

family	modularity model	CR	*p*-value	sample	modularity model (after correction)	CR	*p*-value
Odobenidae							
*Odobenus rosmarus*	face and neurocranium sep.within + same.between	0.804	2.00 × 10^−4^	W	6-clusters same.within + same.between*	0.621	0.000
Otariidae							
*Arctocephalus australis*	6-clusters sep.within + same.between	0.725	2.00 × 10^−4^	W	6-clusters sep.within + same.between		
*Arctocephalus gazella*	6-clusters sep.within + sep.between	0.948	0.005	W	6-clusters sep.within + sep.between		
*Arctocephalus tropicalis*	6-clusters sep.within + sep.between	0.801	2.00 × 10^−4^	W	6-clusters same.within + same.between		
*Callorhinus ursinus*	6-clusters sep.within + sep.between	0.868	0.007	W	6-clusters sep.within + same.between		
*Otaria byronia*	6-clusters sep.within + sep.between	0.983	5.78×10^−2^				
				F	6-clusters same.within + same.between	0.717	0.000
				M	6-clusters same.within + same.between	0.857	0.001
*Zalophus californianus*	6-clusters sep.within + sep.between	0.657	2.00 × 10^−4^	W	face and neurocranium same.within + same.between*	0.621	0.000
Phocidae							
*Cystophora cristata*	face and neurocranium same.within + same.between	0.787	4.00 × 10^−4^	W	face and neurocranium same.within + same.between		
*Halichoerus grypus*	6-clusters sep.within + sep.between	*0.78*	*0.078*				
				F	face and neurocranium sep.within + same.between*	*0.805*	*0.309*
				M	face and neurocranium sep.within + same.between*	*0.777*	*0.29*
*Hydrurga leptonyx*	face and neurocranium same.within + same.between	0.708	2.00 × 10^−4^	W	face and neurocranium same.within + same.between		
*Lobodon carcinophaga*	face and neurocranium same.within + same.between	0.722	2.00 × 10^−4^	W	face and neurocranium same.within + same.between		
*Leptonychotes weddellii*	6-clusters sep.within + sep.between	0.62	2.00 × 10^−4^	W	6-clusters sep.within + sep.between		
*Mirounga leonina*	function sep.within + same.between	0.724	4.00 × 10^−4^	W	function sep.within + same.between		
*Pagophilus groenlandicus*	6-clusters sep.within + same.between	0.649	2.00 × 10^−4^	W	6-clusters sep.within + same.between		
*Phoca vitulina*	face and neurocranium sep.within + same.between	0.733	3.85 × 10^−1^				
				F	face and neurocranium sep.within + same.between	0.716	0.000
				M	6-clusters same.within + same.between*	*0.91*	*0.068*

After a species-level allometric size correction, the best-supported model of modularity for the walrus skull changed to model ‘5’ (i.e. the six-cluster model) with the same magnitudes of integration both across and within modules (table 8). This model was again supported by CR analysis of the corrected data (CR = 0.62, *p* = 0.0002; table 7).

**Table 8.** Summary of the best-supported model of modularity selected with EMMLi analysis per species and their respective posterior probabilities after respective allometric or sex-specific allometric corrections (see main text). Abbreviations for the model parametrization are as follows: whereas ‘sep’ and ‘same’ stand for separate and same integration levels, respectively’, ‘within’ and ‘between’ refer to integration levels within and between modules.

	species	best-supported model	posterior probability
Odobenidae	*Odobenus rosmarus*	6-clusters same.within + same.between	0.511
Otariidae	*Arctocephalus australis*	6-clusters sep.within + same.between	0.688
	*Arctocephalus gazella*	6-clusters sep.within + sep.between	1.000
	*Arctocephalus tropicalis*	6-clusters same.within + same.between	0.531
	*Callorhinus ursinus*	6-clusters sep.within + same.between	0.755
	*Otaria byronia* (females)	6-clusters same.within + same.between	0.365
	*Otaria byronia* (males)	6-clusters same.within + same.between	0.497
	*Zalophus californianus*	face and neurocranium same.within + same.between	0.577
Phocidae	*Cystophora cristata*	face and neurocranium same.within + same.between	0.619
	*Halichoerus grypus* (females)	face and neurocranium sep.within + same.between	0.820
	*Halichoerus grypus* (males)	face and neurocranium sep.within + same.between	0.556
	*Hydrurga leptonyx*	face and neurocranium same.within + same.between	0.389
	*Lobodon carcinophaga*	face and neurocranium same.within + same.between	0.701
	*Leptonychotes weddellii*	6-clusters sep.within + sep.between	0.998
	*Mirounga leonina*	function sep.within + same.between	0.960
	*Pagophilus groenlandicus*	6-clusters sep.within + same.between	0.433
	*Phoca vitulina* (females)	face and neurocranium sep.within + same.between	0.634
	*Phoca vitulina* (males)	6-clusters same.within + same.between	0.819

#### 3.3.2. Otariidae

Before allometric size-correction, all otariid species supported the same model for skull shape organization: the six-cluster model. Additionally, all species but one supported the same parametrization of the model, with different magnitudes of integration both between and within modules. The sole exception was *Arctocephalus australis*, which supported the same levels of integration between modules (table 6). CR analyses significantly supported the respective model for all species (table 7, *p* < 0.05) with the exception of the South American sea lion (*O. byronia*). For *O*. *byronia*, although EMMLi analysis supported the six-cluster model with different intra- and inter-module magnitudes of integration with a relatively high posterior probability of 0.844, and the three other models with the subsequent highest probabilities were variations of the parametrization of the same model (posterior probabilities between 0.075 and 0.005; electronic supplementary material, table S2), CR analysis was not significant for this modular organization (i.e. CR = 0.98, *p* = 0.0578; table 7).

After size correction, all species but one still supported the same six-module model as before correction for allometry (table 8). The single species for which there was a change in the best-supported model after allometric correction was *Zalophus californianus*, for which the best model after correction was the ‘face and neurocranium’ two-module model. After sex-specific allometric correction of *O. byronia*, CR analysis of the six-cluster model was highly significant for both sexes (*p* ≪ 0.05, table 6). CR analysis of *Z. californianus* was also significant for the two-module model after size-correction (*p* ≪ 0.05).

#### 3.3.3. Phocidae

There was a much greater variation in the best-supported models of modularity across phocid species. Of the eight species in this study, four supported the ‘face and neurocranium’ two-module model (*C. cristata*, *H. leptonyx*, *Lo. carcinophaga* and *Ph. vitulina*; table 6). For those, the best-supported parametrization of the model involved same within-species levels of integration across the two partitions, except for the harbour seal (*Ph. vitulina*), for which there were different magnitudes of integration across the two modules. CR analyses of the two-module model were significant for all species (*p* ≪ 0.05, table 7), with the exception of the harbour seal (*Ph. vitulina*, CR = 0.73 and *p* = 0.385).

The six-cluster model was the best supported for three phocid species: *Ha. grypus*, *L. weddellii*, both with different magnitudes of integration within and between modules, and *Pa. groenlandicus*, with separate within-module integration magnitudes but similar magnitudes across modules (table 6). CR analyses of the six-module model were significant for *L. weddellii* and *Pa. groenlandicus* (*p* = 0.0002), but not for *Ha. grypus* (*p* > 0.05). Finally, the best-supported model for the southern elephant seal (*M. leonina*) was the ‘functional groups' model, which was also supported by CR analysis (CR = 0.724, *p* ≪ 0.05).

After allometric size-correction at the species-level for six of the eight species (*C. cristata*, *H. leptonyx*, *L. weddellii*, *Lo. carcinophaga*, *M. leonina* and *Pa. groenlandicus*), there was no change in the best-supported model (table 8). After sex-specific corrections, the best-supported model for both females and males of grey seals (*Ha. grypus*) was the ‘face and neurocranium’ model. However, these results were not supported by CR analysis (*p* ≫ 0.05; table 7). Similar results were found for male grey seal specimens when analysed separately, with a change in the best-supported model, in this case to the six-cluster model, but again without support from CR analysis (*p* > 0.05; tables 7 and 8). When female grey seals were analysed separately, without allometric correction due to the lack of a significant effect of allometry on female skull shape, as noted above, the best-supported model was the ‘face and neurocranium’ model, as for the uncorrected joint analysis of both sexes, and this model was also supported by CR analysis (*p* = 0.0004).

### 3.4. Integration and disparity

The relationship between magnitudes of integration and disparity across modules varied largely with the best-supported model of cranial modularity.

#### 3.4.1. Modules within the ‘face and neurocranium’ model

For all five species that showed strongest support for the two-module model (*O. rosmarus*, *C. cristata*, *H. leptonyx*, *Lo. carcinophaga* and *Ph. vitulina*), the module with the highest integration also presented the greatest disparity (table 9). Nevertheless, which of the two modules presented these higher magnitudes was not consistent across the five species.

**Table 9.** Comparison of integration levels, both as a measure of eigenvalue dispersion and *ρ*, and disparity levels weighted by landmark count per module for the species for which the ‘face and neurocranium’ model of modularity was preferred by the EMMLi analysis. Bold formatting highlights values of integration and disparity that were higher than each variable's mean.

species	module	integration (eigenvalue dispersion)	*ρ* (EMMLi)	disparity
*O. rosmarus*	1	**0.205**	**0.17**	**0.195**
*O. rosmarus*	2	0.160	0.13	0.180
	mean	0.182	0.15	0.188

*C. cristata*	1	**0.242**	0.2	**2.525**
*C. cristata*	2	0.227	0.2	2.260
	mean	0.235		2.392

*H. leptonyx*	1	0.212	0.18	2.233
*H. leptonyx*	2	**0.214**	0.18	**2.242**
	mean	0.213		2.237

*L. carcinophaga*	1	**0.219**	0.18	**2.140**
*L. carcinophaga*	2	0.207	0.18	1.939
	mean	0.213		2.039

*P. vitulina*	1	0.187	0.14	3.367
*P. vitulina*	2	**0.235**	**0.19**	**3.778**
	mean	0.211	0.165	3.572

#### 3.4.2. Modules within the six-cluster model

There was no clear relationship between magnitudes of morphological integration and disparity across the skull modules of the nine species for which this was the best-supported model (i.e. otariids *Ar. australis*, *A. gazelle*, *A. tropicalis*, *C. ursinus*, *O. byronia*, *Z. californianus* and phocids *Ha. grypus*, *Pa. groenlandicus* and *L. weddellii*). However, across all species, there was a strong consistency in which modules were either more integrated (both by *ρ* and eigenvalue dispersion magnitudes) or more disparate. Seven of the listed species displayed the highest levels of integration within modules ‘4’ and ‘5’ (i.e. vault and pterygoid modules, respectively). Additionally, five of those species (i.e. *A. tropicalis*, *C. ursinus*, *L. weddellii*, *O. byronia* and *P. groenlandicus*) also displayed magnitudes of integration for module ‘1’ (i.e. oral module) that were higher than the mean and median values for module integration for the respective species (table 10). With regard to disparity, all species showed magnitudes which were higher than the mean and median for modules ‘1’ and ‘3’ (i.e. oral and molar modules, respectively), whereas two species also presented higher disparity for module ‘5’ (i.e. pterygoid module in *A. gazella*, and *Z. californianus*).

**Table 10.** Comparison of integration levels, both as a measure of eigenvalue dispersion and *ρ*, and disparity levels weighted by landmark count per module for the species for which the ‘six-cluster’ model of modularity was preferred by the EMMLi analysis. Bold formatting highlights values of integration and disparity that were higher than each variable's mean and median. Bold italic formatting highlights the lowest values of integration and disparity.

species	module	integration (eigenvalue dispersion)	*ρ* (EMMLi)	weighted disparity
*A. australis*	1	**0.258**	0.210	**0.389**
*A. australis*	2	***0.174***	***0.170***	***0.087***
*A. australis*	3	0.218	***0.170***	**0.232**
*A. australis*	4	**0.441**	**0.420**	***0.093***
*A. australis*	5	**0.327**	**0.270**	0.148
*A. australis*	6	***0.192***	***0.150***	***0.140***
	mean	0.268	0.232	0.182
	median	0.238	0.190	0.144
*A. gazella*	1	***0.254***	***0.200***	**0.318**
*A. gazella*	2	**0.474**	**0.470**	***0.072***
*A. gazella*	3	***0.200***	***0.170***	**0.355**
*A. gazella*	4	***0.251***	***0.220***	***0.118***
*A. gazella*	5	**0.385**	**0.330**	**0.266**
*A. gazella*	6	**0.324**	**0.290**	***0.181***
	mean	0.315	0.280	0.218
	median	0.289	0.255	0.223
*A. tropicalis*	1	**0.288**	**0.240**	**0.309**
*A. tropicalis*	2	***0.227***	***0.110***	***0.077***
*A. tropicalis*	3	***0.201***	***0.170***	**0.226**
*A. tropicalis*	4	**0.311**	**0.230**	***0.105***
*A. tropicalis*	5	**0.462**	**0.210**	***0.136***
*A. tropicalis*	6	***0.218***	***0.190***	0.160
	mean	0.285	0.192	0.169
	median	0.258	0.200	0.148
*C. ursinus*	1	**0.383**	**0.350**	**0.224**
*C. ursinus*	2	***0.315***	***0.230***	0.146
*C. ursinus*	3	***0.248***	***0.210***	**0.283**
*C. ursinus*	4	**0.467**	**0.430**	***0.101***
*C. ursinus*	5	**0.444**	**0.410**	***0.137***
*C. ursinus*	6	***0.257***	***0.220***	***0.127***
	mean	0.352	0.308	0.170
	median	0.349	0.290	0.142
*H. grypus*	1	***0.252***	***0.210***	**0.318**
*H. grypus*	2	***0.244***	***0.240***	***0.077***
*H. grypus*	3	***0.141***	***0.090***	**0.508**
*H. grypus*	4	**0.437**	**0.420**	***0.144***
*H. grypus*	5	**0.416**	**0.350**	***0.132***
*H. grypus*	6	**0.354**	**0.320**	0.152
	mean	0.307	0.272	0.222
	median	0.303	0.280	0.148
*L. weddellii*	1	**0.295**	**0.250**	**0.335**
*L. weddellii*	2	***0.127***	***0.110***	***0.082***
*L. weddellii*	3	***0.229***	***0.190***	**0.346**
*L. weddellii*	4	**0.424**	**0.390**	***0.065***
*L. weddellii*	5	**0.258**	**0.210**	***0.165***
*L. weddellii*	6	***0.170***	***0.150***	0.179
	mean	0.250	0.217	0.195
	median	0.243	0.200	0.172
*O. byronia*	1	**0.290**	**0.260**	**0.335**
*O. byronia*	2	***0.175***	***0.150***	***0.191***
*O. byronia*	3	***0.233***	***0.190***	**0.267**
*O. byronia*	4	**0.416**	**0.400**	***0.150***
*O. byronia*	5	**0.320**	**0.270**	0.224
*O. byronia*	6	***0.207***	***0.170***	0.197
	mean	0.273	0.240	0.227
	median	0.261	0.225	0.210
*P. groenlandicus*	1	**0.293**	**0.250**	**0.269**
*P. groenlandicus*	2	***0.112***	***0.090***	***0.090***
*P. groenlandicus*	3	***0.200***	0.160	**0.263**
*P. groenlandicus*	4	**0.369**	**0.310**	***0.119***
*P. groenlandicus*	5	0.223	***0.150***	0.153
*P. groenlandicus*	6	***0.184***	0.160	***0.137***
	mean	0.230	0.187	0.172
	median	0.212	0.160	0.145
*Z. californianus*	1	***0.230***	**0.260**	**0.379**
*Z. californianus*	2	**0.326**	***0.150***	***0.071***
*Z. californianus*	3	***0.213***	***0.190***	**0.239**
*Z. californianus*	4	**0.430**	**0.400**	***0.082***
*Z. californianus*	5	**0.313**	**0.270**	**0.196**
*Z. californianus*	6	***0.220***	***0.170***	***0.184***
	mean	0.289	0.240	0.192
	median	0.272	0.225	0.190

#### 3.4.3. Modules within the ‘functional groups' model

For the southern elephant seal (*M. leonina*), there was also no relationship between magnitude of module disparity and integration. The highest integration was observed in module ‘4’ (basicranium), while the highest disparity was in module ‘3’ (vault) (table 11).

**Table 11.** Comparison of integration levels, both as a measure of eigenvalue dispersion and *ρ*, and disparity levels weighted by landmark count per module for the species for which the ‘functional modules’ model of modularity was preferred by the EMMLi analysis. Bold formatting highlights values of integration and disparity that were higher than each variable's mean and median.

species	module	integration (eigenvalue dispersion)	*ρ* (EMMLi)	weighted disparity
*M. leonina*	1	0.231	0.19	0.123
	2	0.226	0.18	0.112
	3	0.246	0.15	**0.313**
	4	**0.331**	**0.3**	*0.136*
	mean	0.259	0.205	0.171
	median	0.239	0.185	0.13

## 4. Discussion

The evolution of shape is dependent on intrinsic characteristics of the phenotype, such as how traits coevolve and respond to selection [7,9,11,15,16,68]. Here, we have demonstrated that ecological shifts, with the opening of niches and exposure to new adaptive optima, may drive evolution changes in patterns of trait covariation by greatly altering selection pressures on a structure. More specifically, our results indicate that the secondary adaptation to the aquatic environment in pinnipeds may have driven reorganization of cranial modularity relative to that observed in terrestrial carnivorans. This effect is particularly apparent within the more divergent and ecologically specialized phocids, which also have a more ancient crown group origin than otariids. More specifically, we hypothesize that greater ecological specialization in phocids may have driven a change in the modular patterns of the skull from a terrestrial ancestor, generating new organization of skull modules across the family. Nevertheless, the results presented here also corroborate the understanding that the effects of integration on shape disparification may rely more strongly on how aligned are the vectors for the directions of selection and major axes of variation than on the magnitude of integration itself, as no straightforward relationship between levels of integration and disparity was found across modules or species.

### 4.1. Shape and modularity

The three pinniped families occupy distinct regions of cranial morphospace (figure 4), indicating that higher-level phylogenetic relationship is a major constraint of morphological variation across pinnipeds, which has also been previously observed in the literature [53,69]. The larger morphospace occupation by the phocids further corroborates the suggestion that these species display more variation in skull shape than do otariids, possibly reflecting greater ecological diversification in phocids, as well as older divergence times among extant taxa [48,49,52–54,70].

This greater morphological disparity in phocids is reflected in the diversity of patterns of cranial modularity reported here. We have demonstrated that, whereas all otariids support the six-cluster model of skull modularity (with the exception of *Zaphilus californianus* after allometric size-correction), there was much greater variation in the best-supported model across phocids, both before and after allometric corrections. As a six-module model appears to represent a relatively conservative pattern of skull shape organization across placental mammals [25,27,44], these results strongly suggest that otariids have not diverged from this general pattern either due to constraints, lack of strong selection or limited time since divergence. By contrast, the variation in patterns of modularity observed across phocid species would suggest that selection for ecomorphological specialization in skull shape across phocids may have reshaped the variance–covariance matrix underlying the evolution of this structure. It has been hypothesized that this secondary aquatic adaptation in pinnipeds has been accompanied by a shift of functions more frequently associated with postcranial elements onto the cranium, such as antagonistic behaviours, sexual display and specializations for prey capture [52,53]. This suggestion is corroborated by the higher variation in patterns of modularity found in seals (phocids) than in otariids, which may also reflect the difference in divergence time between the two families [48,49,53]. The otariid crown group has a more recent origin than that of phocids, and otariids remain more dependent on terrestrial habitats during breeding and weaning of pups and are predominantly generalist feeders. By contrast, phocids demonstrate a greater shift towards aquatic niches throughout their lives and a broader range of dietary specializations, from filter-feeders to specialists on large tetrapod prey [40,49,53,71]. Although this difference between families may be due to distinct selection pressures across their evolutionary history, the earlier divergence of phocids may also have facilitated their greater variation in ecology and morphological traits.

Within phocids, most species which differed from the six-module model (i.e. *C. cristata*, *Ha. grypus*, *H. leptonyx*, *Lo. carcinophaga* and *M. leonina*) have been shown to display morphological changes in their skull morphology that are either correlated with a specialized diet or mating strategy [48,53]. Whereas *L. carcinophaga* (crabeater seal) is a filter feeder and *H. leptonyx* (leopard seal) specializes in large and warm-blooded vertebrate prey, *C. cristata* (hooded seal) and *M. leonina* (southern elephant seal) show specializations with regard to mating displays [47]. Further, *M. leonina* may show modifications of skull shape which correlate with an ability to deep-dive [40,57,58].

Although the influence of size on shape (allometry) was widespread, either on a species- or a sex-specific level, there was little change in the best-supported model of modularity after correcting for allometry. An interesting exception was the walrus' (*O*. *rosmarus*), in which, prior to correction, the favoured model was the partitioning of the skull into two modules (face and neurocranium; [60,65]). This result changed to the general mammalian model of six partitions after correcting for skull allometry. Here, size may have acted as a driver of morphological integration, linking multiple anterior and posterior modules into these two larger partitions, possibly as a consequence of both a specialized diet (i.e. suction feeding) and to accommodate anteriorly localized skull changes caused by the extreme growth of the upper canines.

Within otariids, the only example of a change in best-supported model after allometric size-correction was in the Californian sea lion (*Z. californianus*), and here the change was from the general six-module pattern to the ‘face and neurocranium’ model (tables 6 and 8). This change in best-supported model following an allometric correction suggests that size may not influence skull shape uniformly in this species, and allometric effects differ across skull modules. For this reason, removing allometric effects increases the observed integration (i.e. reduces the modularity) of the cranium. Although there is observed sexual dimorphism in Californian sea lions, our analyses showed that there was no difference in the amount or direction of size-related shape variation between males and females.

Within phocids, changes in the best-supported model after allometric correction occurred only on analyses of skull modularity in the grey seal (*Ha. grypus*) and for the separate analyses of male specimens of the harbour seal (*Ph. vitulina*). Interestingly, in both cases, the model that was best-supported with the EMMLi analyses was not confirmed by the CR test either before or after corrections (tables 6 and 8). These results potentially suggest that the patterns of cranial modularity for the grey seal would be best described by a model not tested here.

Similarly, although still significant when tested with CR analysis, some of the best-supported models from analysis with EMMLi displayed low posterior probabilities (i.e. less than 0.5). Examining the *ρ* values for within and between-modules in these instances showed either very similar or even higher correlation between modules than within them (tables 6 and 8) again suggesting that the models tested here may not be those that best describe cranial modularity in a few pinniped species, especially in phocids.

### 4.2. Integration and disparity

The relationship between magnitude of integration and module disparity varied with the model of modularity that was best supported for each species. Whereas there was also no correlation between trait integration and disparity in the skull modules of the only species to support the ‘functional groups’ model of modularity, the southern elephant seal (*M. leonina*; table 11), a different result was found for the five species which supported the two-module model of ‘face and neurocranium’ (table 9). Here, the most integrated of the modules was also the most disparate. The ‘face’ was more integrated and disparate than neurocranium for the walrus (*O. rosmarus*), *C. cristata* and *L. carcinophaga*, which are species that display great shape changes with regard to specializations for diet or mating display (e.g. great elongation of upper canines, facial bladder and suction feeding). For *P. vitulina,* the neurocranium, rather than the face, was more integrated and disparate. In *H. leptonyx*, integration and disparity had similar magnitudes in both modules.

For the six-module model, which was best supported in nine species, there was no clear relationship between magnitudes of integration and disparity (table 10). Whereas the modules with the lowest integration displayed the highest disparity in three of the studied species (i.e. *A. gazella*, *C. ursinus* and *H. grypus*), in other species, the lowest integration was found in the same module that showed the lowest disparity (*A. australis, L. weddellii* and *P. groenlandicus*). Similarly, in most cases, the most integrated modules only showed intermediate levels of disparity and vice-versa.

Overall, our results show a strong consistency in which modules were the most disparate or the most integrated. The modules with the highest disparity were concentrated in the anterior oral and molar regions, suggesting that diet (i.e. prey acquisition and processing) may be the strongest driver of shape change across these species.

Interestingly, our results concerning the most integrated modules differ from a previous study of skull morphological integration across carnivoran species [27]. Whereas the vault and pterygoid were the modules with the highest level of integration here, Goswami [27] reported them to be relatively weakly integrated when compared with oral–nasal, molar and basicranial regions. Although this discrepancy could be a reflection of a difference in the number of landmarks collected and measures of integration between the two studies, it may also be suggestive of a shift in integration patterns which may be correlated with a major ecological transition. Whereas in this previous study [27], the focus was on fissiped (i.e. terrestrial) carnivoran species, here we focus only on the species which have gone through a secondary aquatic adaptation.

Finally, these results demonstrate that trait integration can have varying effects on trait evolvability and response to selection. Both theoretical and empirical evidence have recently started to accumulate on the dichotomous effect of strong trait integration in promoting but also constraining morphological change across a variety of taxa (albeit most empirical work has been done on tetrapod vertebrates) [2,8,11,12,15,16,28,29,66,68,72–75]. As discussed above, rather than having an uniform effect across structures and taxa, the consequences of high integration for macroevolution may depend more strongly on whether the main axis of shape covariation aligns with the direction of selection (i.e. the line of least resistance) [15,16] than on raw magnitude of integration itself.

Here, this absence of a straightforward relationship is highlighted by the diversity in relationships between magnitudes of morphological integration and disparity across a sample of closely related species. Furthermore, the results discussed here clearly demonstrate that patterns of modularity are themselves evolvable and responsive to selection, and repartitioning of modules may occur to accommodate strong selection for functional and morphological changes [1,27,76–81].

In conclusion, we have shown that the patterns of cranial phenotypic modularity are not uniform across the three families of pinnipeds. Importantly, the variation in pattern of modularity may reflect differences in time since divergence of the extant members of Otariidae and Phocidae, or with the shift towards a more specialized aquatic niche and generally greater ecological diversity in phocids. Additionally, we have shown that there is no uniform relationship between magnitude of morphological integration and amount of disparity in individual modules both across and within pinniped families. Whereas there is no obvious correlation between those two variables for species that follow either the six-cluster or the functional models of modularity, there was a direct and positive correlation for those under the ‘face and neurocranium’ model. Finally, we suggest that the increased ecological specialization observed in phocids may have driven the multiple shifts in the pattern of cranial modularity that is otherwise conserved in most therian mammals, including the other pinniped clades.

## Supplementary Material

Table S1

Reviewer comments

## Supplementary Material

Table S2

## Supplementary Material

Dataset

## Data accessibility

The datasets supporting this article have been made available as part of the electronic supplementary material. The supplied tables detail specimen location and accession number, and the complete dataset collected and analysed here.
