# Supplementary material for: Shifts in cranial integration associated with ecological specialization in pinnipeds (Mammalia, Carnivora)
Source: R Soc Open Sci. 2019 Mar 27;6(3):190201. doi: 10.1098/rsos.190201 (PMC6458409; doi:10.1098/rsos.190201)
Supplement: Table S1 [file rsos190201supp1.docx]

Table S1: Collection and museum number information for all pinniped specimens analysed:

| Family | Sex | Museum | Acronym |
| --- | --- | --- | --- |
| Odobenidae | Female | Museum fur Naturkunde | MNHB-35672 |
| Odobenidae | Female | Museum fur Naturkunde | MNHB-46496 |
| Odobenidae | Female | Museum fur Naturkunde | MNHB-70679 |
| Odobenidae | Female | Museum fur Naturkunde | MNHB-no number |
| Odobenidae | Female | British Natural History Museum | BMNH-1855.11.26.38 |
| Odobenidae | Female | British Natural History Museum | BMNH-1926.12.2.1 |
| Odobenidae | Female | Museum National d'Histoire Naturelle | MNHN-1933-561 |
| Odobenidae | Female | British Natural History Museum | BMNH-1948.4.24.1 |
| Odobenidae | Female | British Natural History Museum | BMNH-3 |
| Odobenidae | Female | British Natural History Museum | BMNH-331.b |
| Odobenidae | Female | Museum of Zoology Cambridge | MZC-K.7481 |
| Odobenidae | Female | Museum of Zoology Cambridge | MZC-K.7483 |
| Odobenidae | Female | Museum of Zoology Cambridge | MZC-K.7490 |
| Odobenidae | Female | Oxford University Museum of Natural History | OUM-13863 |
| Odobenidae | Male | Museum fur Naturkunde | MNHB-29553 |
| Odobenidae | Male | Museum fur Naturkunde | MNHB-43047 |
| Odobenidae | Male | Museum fur Naturkunde | MNHB-5928 |
| Odobenidae | Male | Museum fur Naturkunde | MNHB-70677 |
| Odobenidae | Male | Museum fur Naturkunde | MNHB-93323 |
| Odobenidae | Male | British Natural History Museum | BMNH-1842.10.8.2 |
| Odobenidae | Male | British Natural History Museum | BMNH-1903.1.25.1 |
| Odobenidae | Male | British Natural History Museum | BMNH-1926.12.2.2 |
| Odobenidae | Male | British Natural History Museum | BMNH-1926.12.2.4 |
| Odobenidae | Male | British Natural History Museum | BMNH-1926.12.2.5 |
| Odobenidae | Male | British Natural History Museum | BMNH-1926.12.2.6 |
| Odobenidae | Male | British Natural History Museum | BMNH-1926.9.27.1 |
| Odobenidae | Male | Museum National d'Histoire Naturelle | MNHN-1928-312 |
| Odobenidae | Male | British Natural History Museum | BMNH-1950.9.27.1 |
| Odobenidae | Male | Museum fur Naturkunde | MNHB-1999.20 |
| Odobenidae | Male | Museum fur Naturkunde | MNHB-39.4474 |
| Odobenidae | Male | Museum of Zoology Cambridge | MZC-K.7482 |
| Odobenidae | Male | Grant Museum of Zoology - UCL | UCL-GMZ-Z2270 |
| Odobenidae | Male | Museum fur Naturkunde | MNHB-no history |
| Odobenidae | Male | Oxford University Museum of Natural History | OUM-13862 |
| Odobenidae | Unidentified | Museum fur Naturkunde | MNHB-1939 |
| Otariidae | Female | British Natural History Museum | BMNH-18979.8.21.5 |
| Otariidae | Female | Museum fur Naturkunde | MNHB-1984.919 |
| Otariidae | Female | British Natural History Museum | BMNH-83.314 |
| Otariidae | Female | British Natural History Museum | BMNH-84.910 |
| Otariidae | Female | British Natural History Museum | BMNH-84.915 |
| Otariidae | Female | British Natural History Museum | BMNH-84.916 |
| Otariidae | Female | British Natural History Museum | BMNH-84.917 |
| Otariidae | Female | British Natural History Museum | BMNH-84.925 |
| Otariidae | Female | British Natural History Museum | BMNH-84.937 |
| Otariidae | Female | British Natural History Museum | BMNH-84.957 |
| Otariidae | Female | British Natural History Museum | BMNH-84.967 |
| Otariidae | Female | British Natural History Museum | BMNH-84.968 |
| Otariidae | Female | British Natural History Museum | BMNH-84.974 |
| Otariidae | Male | British Natural History Museum | BMNH-1949.3.17.1 |
| Otariidae | Male | British Natural History Museum | BMNH-1949.3.17.11 |
| Otariidae | Male | British Natural History Museum | BMNH-1949.3.17.13 |
| Otariidae | Male | British Natural History Museum | BMNH-1949.3.17.15 |
| Otariidae | Male | British Natural History Museum | BMNH-1949.3.17.19 |
| Otariidae | Male | British Natural History Museum | BMNH-1949.3.17.21 |
| Otariidae | Male | British Natural History Museum | BMNH-1949.3.17.23 |
| Otariidae | Male | British Natural History Museum | BMNH-1949.3.17.34 |
| Otariidae | Male | British Natural History Museum | BMNH-1949.3.17.5 |
| Otariidae | Male | British Natural History Museum | BMNH-1949.3.17.58 |
| Otariidae | Male | British Natural History Museum | BMNH-1949.3.17.6 |
| Otariidae | Male | British Natural History Museum | BMNH-1949.3.17.8 |
| Otariidae | Male | British Natural History Museum | BMNH-1949.3.17.9 |
| Otariidae | Male | British Natural History Museum | BMNH-1950.11.14.1 |
| Otariidae | Male | British Natural History Museum | BMNH-1984.942 |
| Otariidae | Male | British Natural History Museum | BMNH-84.911 |
| Otariidae | Male | British Natural History Museum | BMNH-84.912 |
| Otariidae | Male | British Natural History Museum | BMNH-84.921 |
| Otariidae | Male | British Natural History Museum | BMNH-84.923 |
| Otariidae | Male | British Natural History Museum | BMNH-1842.10.8.2 |
| Otariidae | Male | British Natural History Museum | BMNH-84.930 |
| Otariidae | Male | British Natural History Museum | BMNH-84.932 |
| Otariidae | Male | British Natural History Museum | BMNH-84.933 |
| Otariidae | Male | British Natural History Museum | BMNH-84.935 |
| Otariidae | Male | British Natural History Museum | BMNH-84.947 |
| Otariidae | Male | British Natural History Museum | BMNH-84.948 |
| Otariidae | Male | British Natural History Museum | BMNH-84.973 |
| Otariidae | Male | British Natural History Museum | BMNH-84.978 |
| Otariidae | Male | British Natural History Museum | BMNH-84918 |
| Otariidae | Female | Museum fur Naturkunde | MNHB-86626 |
| Otariidae | Female | British Natural History Museum | BMNH-1958.4.24.4 |
| Otariidae | Female | British Natural History Museum | BMNH-1960.8.10.14 |
| Otariidae | Female | British Natural History Museum | BMNH-1960.8.10.29 |
| Otariidae | Female | British Natural History Museum | BMNH-1960.8.10.31 |
| Otariidae | Female | British Natural History Museum | BMNH-1960.8.10.32 |
| Otariidae | Female | British Natural History Museum | BMNH-1960.8.10.36 |
| Otariidae | Female | British Natural History Museum | BMNH-1960.8.10.37 |
| Otariidae | Female | British Natural History Museum | BMNH-1960.8.10.38 |
| Otariidae | Female | British Natural History Museum | BMNH-1960.8.10.39 |
| Otariidae | Female | British Natural History Museum | BMNH-1960.8.10.41 |
| Otariidae | Female | British Natural History Museum | BMNH-1960.8.10.5 |
| Otariidae | Female | British Natural History Museum | BMNH-1960.8.10.7 |
| Otariidae | Female | British Natural History Museum | BMNH-1960.8.10.8 |
| Otariidae | Female | British Natural History Museum | BMNH-1962.6.14.13 |
| Otariidae | Female | British Natural History Museum | BMNH-1962.6.14.14 |
| Otariidae | Female | British Natural History Museum | BMNH-1991.40 |
| Otariidae | Female | Museum of Zoology Cambridge | MZC-K.7321 |
| Otariidae | Female | Museum of Zoology Cambridge | MZC-K.7321.D |
| Otariidae | Female | Museum of Zoology Cambridge | MZC-K.7321.E |
| Otariidae | Female | Museum of Zoology Cambridge | MZC-K.7321.F |
| Otariidae | Female | Museum of Zoology Cambridge | MZC-K.7321.J |
| Otariidae | Male | British Natural History Museum | BMNH-1930.81.10.57 |
| Otariidae | Male | British Natural History Museum | BMNH-1960.8.10.2 |
| Otariidae | Male | British Natural History Museum | BMNH-1960.8.10.24 |
| Otariidae | Male | British Natural History Museum | BMNH-1960.8.10.46 |
| Otariidae | Male | British Natural History Museum | BMNH-1960.8.10.49 |
| Otariidae | Male | British Natural History Museum | BMNH-1960.8.10.50 |
| Otariidae | Male | British Natural History Museum | BMNH-1960.8.10.51 |
| Otariidae | Male | British Natural History Museum | BMNH-1960.8.10.54 |
| Otariidae | Male | British Natural History Museum | BMNH-1960.8.10.55 |
| Otariidae | Male | British Natural History Museum | BMNH-1962.10.16.1 |
| Otariidae | Male | British Natural History Museum | BMNH-1964.9.22.1 |
| Otariidae | Male | British Natural History Museum | BMNH-1964.9.22.2 |
| Otariidae | Male | British Natural History Museum | BMNH-1980.8.4.4 |
| Otariidae | Male | British Natural History Museum | BMNH-1981.1239 |
| Otariidae | Male | British Natural History Museum | BMNH-1981.1240 |
| Otariidae | Male | British Natural History Museum | BMNH-1981.1243 |
| Otariidae | Male | British Natural History Museum | BMNH-1981.125 |
| Otariidae | Male | British Natural History Museum | BMNH-1981.1250 |
| Otariidae | Male | British Natural History Museum | BMNH-1981.1251 |
| Otariidae | Male | British Natural History Museum | BMNH-1981.1254 |
| Otariidae | Male | British Natural History Museum | BMNH-1981.1255 |
| Otariidae | Male | British Natural History Museum | BMNH-1981.1257 |
| Otariidae | Male | Museum of Zoology Cambridge | MZC-K.7321.L |
| Otariidae | Male | Museum of Zoology Cambridge | MZC-K.7321.O |
| Otariidae | Male | British Natural History Museum | BNHM-no.number |
| Otariidae | Female | Museum National d'Histoire Naturelle | MNHN-1875-623 |
| Otariidae | Female | Zoologisch Museum Amsterdam | ZMA-19.585 |
| Otariidae | Female | British Natural History Museum | BMNH-1955.3.14.18 |
| Otariidae | Female | British Natural History Museum | BMNH-1955.3.14.5 |
| Otariidae | Female | British Natural History Museum | BMNH-1955.3.14.7 |
| Otariidae | Female | British Natural History Museum | BMNH-1957.4.23.20 |
| Otariidae | Female | Museum National d'Histoire Naturelle | MNHN-1962-4153 |
| Otariidae | Female | Museum National d'Histoire Naturelle | MNHN-1963-30 |
| Otariidae | Female | British Natural History Museum | BMNH-1968.4.4.1 |
| Otariidae | Female | British Natural History Museum | BMNH-1968.4.4.2 |
| Otariidae | Female | British Natural History Museum | BMNH-1968.4.4.9 |
| Otariidae | Female | Museum National d'Histoire Naturelle | MNHN-1976-378 |
| Otariidae | Female | Museum National d'Histoire Naturelle | MNHN-1976-384 |
| Otariidae | Female | Museum National d'Histoire Naturelle | MNHN-1986-072 |
| Otariidae | Male | Zoologisch Museum Amsterdam | ZMA-19.584 |
| Otariidae | Male | British Natural History Museum | BMNH-1955.3.14.2 |
| Otariidae | Male | British Natural History Museum | BMNH-1957.4.23.10 |
| Otariidae | Male | British Natural History Museum | BMNH-1957.4.23.11 |
| Otariidae | Male | British Natural History Museum | BMNH-1957.8.1.1 |
| Otariidae | Male | British Natural History Museum | BMNH-1957.8.1.2 |
| Otariidae | Male | Museum National d'Histoire Naturelle | MNHN-1962-4146 |
| Otariidae | Male | Museum National d'Histoire Naturelle | MNHN-1962-4147 |
| Otariidae | Male | Museum National d'Histoire Naturelle | MNHN-1962-4148 |
| Otariidae | Male | Museum National d'Histoire Naturelle | MNHN-1962-4150 |
| Otariidae | Male | Museum National d'Histoire Naturelle | MNHN-1962-4151 |
| Otariidae | Male | Museum National d'Histoire Naturelle | MNHN-1971-118 |
| Otariidae | Male | Museum National d'Histoire Naturelle | MNHN-1972-644 |
| Otariidae | Male | Museum National d'Histoire Naturelle | MNHN-1978-334 |
| Otariidae | Male | Museum National d'Histoire Naturelle | MNHN-1978-339 |
| Otariidae | Female | Museum fur Naturkunde | MNHB-37403 |
| Otariidae | Female | Museum fur Naturkunde | MNHB-5648 |
| Otariidae | Female | Museum fur Naturkunde | MNHB-74328 |
| Otariidae | Female | British Natural History Museum | BMNH-1891.12.18.10 |
| Otariidae | Female | British Natural History Museum | BMNH-1891.18.12.11 |
| Otariidae | Female | British Natural History Museum | BMNH-1928.4.21.52 |
| Otariidae | Female | British Natural History Museum | BMNH-1928.4.21.53 |
| Otariidae | Female | British Natural History Museum | BMNH-1928.4.21.63 |
| Otariidae | Female | British Natural History Museum | BMNH-1928.4.21.65 |
| Otariidae | Female | British Natural History Museum | BMNH-1950.3.29.7 |
| Otariidae | Female | British Natural History Museum | BMNH-1960.5.2.2 |
| Otariidae | Female | Museum National d'Histoire Naturelle | MNHN-1978-198 |
| Otariidae | Male | Museum fur Naturkunde | MNHB-37402 |
| Otariidae | Male | Museum fur Naturkunde | MNHB-5627 |
| Otariidae | Male | Zoologisch Museum Amsterdam | ZMA-17.132 |
| Otariidae | Male | Zoologisch Museum Amsterdam | ZMA-17.992 |
| Otariidae | Male | British Natural History Museum | BMNH-1878.5.10.2 |
| Otariidae | Male | British Natural History Museum | BMNH-1891.12.18.9 |
| Otariidae | Male | British Natural History Museum | BMNH-1893.1.28.2 |
| Otariidae | Male | British Natural History Museum | BMNH-1928.4.21.59 |
| Otariidae | Male | British Natural History Museum | BMNH-1928.4.21.60 |
| Otariidae | Male | British Natural History Museum | BMNH-1928.4.21.61 |
| Otariidae | Male | British Natural History Museum | BMNH-1950.3.29.6 |
| Otariidae | Male | Museum fur Naturkunde | MNHB-26 |
| Otariidae | Male | Museum fur Naturkunde | MNHB-27 |
| Otariidae | Female | Museum fur Naturkunde | MNHB-288 |
| Otariidae | Female | British Natural History Museum | BMNH-1900.5.7.10 |
| Otariidae | Female | British Natural History Museum | BMNH-1939.1.21.104 |
| Otariidae | Female | British Natural History Museum | BMNH-1939.1.21.106 |
| Otariidae | Female | British Natural History Museum | BMNH-1939.1.21.107 |
| Otariidae | Female | British Natural History Museum | BMNH-1939.1.21.108 |
| Otariidae | Female | British Natural History Museum | BMNH-1939.1.21.109 |
| Otariidae | Female | British Natural History Museum | BMNH-1939.1.21.112 |
| Otariidae | Female | British Natural History Museum | BMNH-1939.1.21.113 |
| Otariidae | Female | British Natural History Museum | BMNH-1939.1.21.114 |
| Otariidae | Female | British Natural History Museum | BMNH-1939.1.21.115 |
| Otariidae | Female | British Natural History Museum | BMNH-1939.1.21.116 |
| Otariidae | Female | British Natural History Museum | BMNH-1939.1.21.118 |
| Otariidae | Female | British Natural History Museum | BMNH-1939.1.21.119 |
| Otariidae | Female | British Natural History Museum | BMNH-1939.1.21.120 |
| Otariidae | Female | British Natural History Museum | BMNH-1939.1.21.122 |
| Otariidae | Female | British Natural History Museum | BMNH-1939.1.21.71 |
| Otariidae | Female | British Natural History Museum | BMNH-1939.1.21.72 |
| Otariidae | Female | British Natural History Museum | BMNH-1939.1.21.76 |
| Otariidae | Female | British Natural History Museum | BMNH-1939.1.21.77 |
| Otariidae | Female | British Natural History Museum | BMNH-1939.1.21.78 |
| Otariidae | Female | British Natural History Museum | BMNH-1939.1.21.81 |
| Otariidae | Female | British Natural History Museum | BMNH-1939.1.21.83 |
| Otariidae | Female | British Natural History Museum | BMNH-1939.1.21.84 |
| Otariidae | Female | British Natural History Museum | BMNH-1939.1.21.85 |
| Otariidae | Female | British Natural History Museum | BMNH-1939.1.21.86 |
| Otariidae | Female | British Natural History Museum | BMNH-1939.1.21.89 |
| Otariidae | Female | British Natural History Museum | BMNH-1949.3.17.83 |
| Otariidae | Female | British Natural History Museum | BMNH-84.984 |
| Otariidae | Female | British Natural History Museum | BMNH-OfFA-no.number |
| Otariidae | Male | British Natural History Museum | BMNH-OfMA.no.number |
| Otariidae | Male | British Natural History Museum | BMNH-1851.5.5.51 |
| Otariidae | Male | British Natural History Museum | BMNH-1869.2.24.1 |
| Otariidae | Male | British Natural History Museum | BMNH-1869.8.10.1 |
| Otariidae | Male | British Natural History Museum | BMNH-1925.12.17.11 |
| Otariidae | Male | British Natural History Museum | BMNH-1939.1.21.163 |
| Otariidae | Male | British Natural History Museum | BMNH-1939.1.21.177 |
| Otariidae | Male | British Natural History Museum | BMNH-1939.1.21.180 |
| Otariidae | Male | British Natural History Museum | BMNH-1950.11.6.1 |
| Otariidae | Male | British Natural History Museum | BMNH-1959.12.4.6 |
| Otariidae | Male | British Natural History Museum | BMNH-335m |
| Otariidae | Male | Museum of Zoology Cambridge | MZC-907.AB |
| Otariidae | Male | Museum of Zoology Cambridge | MZC-K.7029 |
| Otariidae | Male | Museum of Zoology Cambridge | MZC-K.7030 |
| Otariidae | Male | Oxford University Museum of Natural History | OUM-13885 |
| Otariidae | Male | Oxford University Museum of Natural History | OUM-13887 |
| Otariidae | Male | British Natural History Museum | BMNH-nonumber2 |
| Otariidae | Male | British Natural History Museum | BMNH-sem.numero.WS.479 |
| Otariidae | Female | Museum fur Naturkunde | MNHB-31399 |
| Otariidae | Female | Museum fur Naturkunde | MNHB-43163 |
| Otariidae | Female | Zoologisch Museum Amsterdam | ZMA-11.126 |
| Otariidae | Female | Zoologisch Museum Amsterdam | ZMA-17.819 |
| Otariidae | Female | Museum National d'Histoire Naturelle | MNHN-1882-190 |
| Otariidae | Female | British Natural History Museum | BMNH-1903.10.11.4 |
| Otariidae | Female | British Natural History Museum | BMNH-1903.10.11.6 |
| Otariidae | Female | Museum National d'Histoire Naturelle | MNHN-1944-276 |
| Otariidae | Female | British Natural History Museum | BMNH-1951.3.6.2 |
| Otariidae | Female | British Natural History Museum | BMNH-1951.3.62.2 |
| Otariidae | Female | British Natural History Museum | BMNH-1954.2.9.1 |
| Otariidae | Female | Museum National d'Histoire Naturelle | MNHN-1962-1155 |
| Otariidae | Female | Museum National d'Histoire Naturelle | MNHN-1962-1158 |
| Otariidae | Female | Zoologisch Museum Amsterdam | ZMA-21.902 |
| Otariidae | Female | Zoologisch Museum Amsterdam | ZMA-22.956 |
| Otariidae | Female | Zoologisch Museum Amsterdam | ZMA-667 |
| Otariidae | Female | Grant Museum of Zoology - UCL | UCL-GMZ-Z301 |
| Otariidae | Male | Museum fur Naturkunde | MNHB-61617 |
| Otariidae | Male | Museum fur Naturkunde | MNHB-72821 |
| Otariidae | Male | Museum fur Naturkunde | MNHB-92601 |
| Otariidae | Male | Zoologisch Museum Amsterdam | ZMA-11.124 |
| Otariidae | Male | Zoologisch Museum Amsterdam | ZMA-11.125 |
| Otariidae | Male | Zoologisch Museum Amsterdam | ZMA-1698 |
| Otariidae | Male | Museum National d'Histoire Naturelle | MNHN-1879-269 |
| Otariidae | Male | Museum National d'Histoire Naturelle | MNHN-1879-270 |
| Otariidae | Male | Museum National d'Histoire Naturelle | MNHN-1879-271 |
| Otariidae | Male | Museum National d'Histoire Naturelle | MNHN-1897-22 |
| Otariidae | Male | Museum National d'Histoire Naturelle | MNHN-1901-646 |
| Otariidae | Male | Museum National d'Histoire Naturelle | MNHN-1934-528 |
| Otariidae | Male | Museum National d'Histoire Naturelle | MNHN-1962-1141 |
| Otariidae | Male | Museum National d'Histoire Naturelle | MNHN-1962-1142 |
| Otariidae | Male | Museum National d'Histoire Naturelle | MNHN-1962-1146 |
| Otariidae | Male | Museum National d'Histoire Naturelle | MNHN-1962-1148 |
| Otariidae | Male | Museum National d'Histoire Naturelle | MNHN-1962-1149 |
| Otariidae | Male | Museum National d'Histoire Naturelle | MNHN-1962-1156 |
| Otariidae | Male | Zoologisch Museum Amsterdam | ZMA-20.346 |
| Otariidae | Male | Zoologisch Museum Amsterdam | ZMA-24.440 |
| Otariidae | Male | Museum National d'Histoire Naturelle | MNHN-7394 |
| Otariidae | Male | Museum National d'Histoire Naturelle | MNHN-Otarie |
| Otariidae | Male | University of Edinburgh | UE-NHC-XH13-3.1 |
| Otariidae | Male | University of Edinburgh | UE-NHC-XH13-3.5 |
| Otariidae | Male | University of Edinburgh | UE-NHC-XH13-3.6 |
| Phocidae | Female | Museum fur Naturkunde | MNHB-16322 |
| Phocidae | Female | Museum fur Naturkunde | MNHB-33244 |
| Phocidae | Female | Zoologisch Museum Amsterdam | ZMA-16.338 |
| Phocidae | Female | Zoologisch Museum Amsterdam | ZMA-16.339 |
| Phocidae | Female | Zoologisch Museum Amsterdam | ZMA-16.343 |
| Phocidae | Female | Zoologisch Museum Amsterdam | ZMA-16.344 |
| Phocidae | Female | Zoologisch Museum Amsterdam | ZMA-16.345 |
| Phocidae | Female | Zoologisch Museum Amsterdam | ZMA-18.060 |
| Phocidae | Female | Zoologisch Museum Amsterdam | ZMA-18.066 |
| Phocidae | Female | Zoologisch Museum Amsterdam | ZMA-18.098 |
| Phocidae | Female | British Natural History Museum | BMNH-1844.6.23.1 |
| Phocidae | Female | British Natural History Museum | BMNH-1890.8.1.4 |
| Phocidae | Female | Zoologisch Museum Amsterdam | ZMA-25.448 |
| Phocidae | Female | British Natural History Museum | BNHM-332.d |
| Phocidae | Female | British Natural History Museum | BMNH-332.g |
| Phocidae | Female | British Natural History Museum | BMNH-332.h |
| Phocidae | Female | Museum fur Naturkunde | MNHB-33244 |
| Phocidae | Male | Museum fur Naturkunde | MNHB-3296 |
| Phocidae | Male | Museum fur Naturkunde | MNHB-33258 |
| Phocidae | Male | Museum fur Naturkunde | MNHB-ohne Fundort |
| Phocidae | Male | Zoologisch Museum Amsterdam | ZMA-16.334 |
| Phocidae | Male | Zoologisch Museum Amsterdam | ZMA-16.336 |
| Phocidae | Male | Zoologisch Museum Amsterdam | ZMA-16.336 |
| Phocidae | Male | Zoologisch Museum Amsterdam | ZMA-16.337 |
| Phocidae | Male | Zoologisch Museum Amsterdam | ZMA-16.340 |
| Phocidae | Male | Zoologisch Museum Amsterdam | ZMA-16.341 |
| Phocidae | Male | Zoologisch Museum Amsterdam | ZMA-16.346 |
| Phocidae | Male | Zoologisch Museum Amsterdam | ZMA-16.347 |
| Phocidae | Male | Zoologisch Museum Amsterdam | ZMA-16.436 |
| Phocidae | Male | Zoologisch Museum Amsterdam | ZMA-18.059 |
| Phocidae | Male | Zoologisch Museum Amsterdam | ZMA-18.062 |
| Phocidae | Male | Zoologisch Museum Amsterdam | ZMA-18.063 |
| Phocidae | Male | Zoologisch Museum Amsterdam | ZMA-18.065 |
| Phocidae | Male | Zoologisch Museum Amsterdam | ZMA-18.067 |
| Phocidae | Male | Zoologisch Museum Amsterdam | ZMA-18.069 |
| Phocidae | Male | Zoologisch Museum Amsterdam | ZMA-18.081 |
| Phocidae | Male | Zoologisch Museum Amsterdam | ZMA-18.088 |
| Phocidae | Male | Zoologisch Museum Amsterdam | ZMA-24.696 |
| Phocidae | Male | Museum fur Naturkunde | MNHB-3 |
| Phocidae | Unidentified | Museum fur Naturkunde | MNHB-33255 |
| Phocidae | Unidentified | British Natural History Museum | BMNH-1843.10.7.7 |
| Phocidae | Unidentified | British Natural History Museum | BMNH-1844.2.2.1 |
| Phocidae | Unidentified | British Natural History Museum | BMNH-1907.9.4.4 |
| Phocidae | Unidentified | Museum National d'Histoire Naturelle | MNHN-1929-226 |
| Phocidae | Unidentified | British Natural History Museum | BMNH-1938.6.28.6 |
| Phocidae | Unidentified | British Natural History Museum | BMNH-1949.2.3.8 |
| Phocidae | Unidentified | Museum National d'Histoire Naturelle | MNHN-2007-406 |
| Phocidae | Unidentified | Zoologisch Museum Amsterdam | ZMA-25.384 |
| Phocidae | Unidentified | Zoologisch Museum Amsterdam | ZMA-25.384 |
| Phocidae | Unidentified | Museum of Zoology Cambridge | MZC-K.7741 |
| Phocidae | Female | Museum fur Naturkunde | MNHB-26346 |
| Phocidae | Female | British Natural History Museum | BMNH-1934.6.20.1 |
| Phocidae | Female | British Natural History Museum | BMNH-1938.3.12.1 |
| Phocidae | Female | British Natural History Museum | BMNH-1951.11.28.1 |
| Phocidae | Female | British Natural History Museum | BMNH-196.5.18.13 |
| Phocidae | Female | British Natural History Museum | BMNH-196.5.18.23 |
| Phocidae | Female | British Natural History Museum | BMNH-196.5.18.33 |
| Phocidae | Female | British Natural History Museum | BMNH-196.5.18.36 |
| Phocidae | Female | British Natural History Museum | BMNH-1961.1.23.3 |
| Phocidae | Female | British Natural History Museum | BMNH-1961.1.23.4 |
| Phocidae | Female | British Natural History Museum | BMNH-1961.1.23.7 |
| Phocidae | Female | British Natural History Museum | BMNH-1961.5.18.25 |
| Phocidae | Female | British Natural History Museum | BMNH-1961.5.18.26 |
| Phocidae | Female | British Natural History Museum | BMNH-1961.5.18.27 |
| Phocidae | Female | British Natural History Museum | BMNH-1961.5.18.30 |
| Phocidae | Female | British Natural History Museum | BMNH-1961.5.18.31 |
| Phocidae | Female | British Natural History Museum | BMNH-1961.5.18.32 |
| Phocidae | Female | British Natural History Museum | BMNH-1961.5.18.33 |
| Phocidae | Female | British Natural History Museum | BMNH-1961.5.18.34 |
| Phocidae | Female | British Natural History Museum | BMNH-1961.5.18.35 |
| Phocidae | Female | British Natural History Museum | BMNH-1961.5.18.36 |
| Phocidae | Female | British Natural History Museum | BMNH-1961.5.18.37 |
| Phocidae | Female | British Natural History Museum | BMNH-88.328 |
| Phocidae | Female | Museum of Zoology Cambridge | MZC-K.7943 |
| Phocidae | Male | Museum fur Naturkunde | MNHB-31975 |
| Phocidae | Male | Museum fur Naturkunde | MNHB-56792 |
| Phocidae | Male | Zoologisch Museum Amsterdam | ZMA-14.514 |
| Phocidae | Male | British Natural History Museum | BMNH-1845.3.17.8 |
| Phocidae | Male | British Natural History Museum | BMNH-1950.1.23.5 |
| Phocidae | Male | British Natural History Museum | BMNH-1956.9.26.4 |
| Phocidae | Male | British Natural History Museum | BMNH-1961.1.23.8 |
| Phocidae | Male | British Natural History Museum | BMNH-1961.5.18.10 |
| Phocidae | Male | British Natural History Museum | BMNH-1961.5.18.11 |
| Phocidae | Male | British Natural History Museum | BMNH-1961.5.18.12 |
| Phocidae | Male | British Natural History Museum | BMNH-1961.5.18.13 |
| Phocidae | Male | British Natural History Museum | BMNH-1961.5.18.14 |
| Phocidae | Male | British Natural History Museum | BMNH-1961.5.18.15 |
| Phocidae | Male | British Natural History Museum | BMNH-1961.5.18.16 |
| Phocidae | Male | British Natural History Museum | BMNH-1961.5.18.17 |
| Phocidae | Male | British Natural History Museum | BMNH-1962.3.6.1 |
| Phocidae | Male | Museum National d'Histoire Naturelle | MNHN-1978-48 |
| Phocidae | Male | Museum National d'Histoire Naturelle | MNHN-1991-723 |
| Phocidae | Male | Zoologisch Museum Amsterdam | ZMA-5845 |
| Phocidae | Male | Zoologisch Museum Amsterdam | ZMA-no.number |
| Phocidae | Unidentified | Zoologisch Museum Amsterdam | ZMA.1502 |
| Phocidae | Unidentified | Zoologisch Museum Amsterdam | ZMA.26584 |
| Phocidae | Unidentified | British Natural History Museum | BMNH-1955.9.23.1 |
| Phocidae | Unidentified | Museum of Zoology Cambridge | MZC-K.7941 |
| Phocidae | Unidentified | Grant Museum of Zoology - UCL | UCL-GMZ-Z1123 |
| Phocidae | Unidentified | University of Edinburgh | UE-NHC-XH13-4.1 |
| Phocidae | Female | British Natural History Museum | BMNH-1854.8.25.5 |
| Phocidae | Female | British Natural History Museum | BMNH-1939.12.3.2 |
| Phocidae | Female | British Natural History Museum | BMNH-1939.2.11.13 |
| Phocidae | Female | British Natural History Museum | BMNH-1939.2.11.18 |
| Phocidae | Female | British Natural History Museum | BMNH-1939.2.11.23 |
| Phocidae | Female | British Natural History Museum | BMNH-1939.2.25.2 |
| Phocidae | Female | British Natural History Museum | BMNH-1939.2.25.4 |
| Phocidae | Female | British Natural History Museum | BMNH-1939.2.25.5 |
| Phocidae | Female | British Natural History Museum | BMNH-1939.2.25.7 |
| Phocidae | Female | British Natural History Museum | BMNH-1939.4.29.5 |
| Phocidae | Female | British Natural History Museum | BMNH-1940.4.6.126 |
| Phocidae | Female | British Natural History Museum | BMNH-1940.4.6.127 |
| Phocidae | Female | British Natural History Museum | BMNH-1940.4.6.14 |
| Phocidae | Female | British Natural History Museum | BMNH-1940.4.6.142 |
| Phocidae | Female | British Natural History Museum | BMNH-1940.4.6.144 |
| Phocidae | Female | British Natural History Museum | BMNH-1940.4.6.16 |
| Phocidae | Female | British Natural History Museum | BMNH-1940.4.6.28 |
| Phocidae | Female | British Natural History Museum | BMNH-1940.4.6.81 |
| Phocidae | Female | British Natural History Museum | BMNH-1959.12.17.4 |
| Phocidae | Female | British Natural History Museum | BMNH-1959.12.4.3 |
| Phocidae | Female | British Natural History Museum | BMNH-sem.numero |
| Phocidae | Male | British Natural History Museum | BMNH-1901.1.4.15 |
| Phocidae | Male | British Natural History Museum | BMNH-1908.2.20.54 |
| Phocidae | Male | British Natural History Museum | BMNH-1914.1.29.2 |
| Phocidae | Male | British Natural History Museum | BMNH-1939.2.11.10 |
| Phocidae | Male | British Natural History Museum | BMNH-1939.2.11.11 |
| Phocidae | Male | British Natural History Museum | BMNH-1939.2.11.12 |
| Phocidae | Male | British Natural History Museum | BMNH-1939.2.11.15 |
| Phocidae | Male | British Natural History Museum | BMNH-1939.2.11.20 |
| Phocidae | Male | British Natural History Museum | BMNH-1939.2.11.21 |
| Phocidae | Male | British Natural History Museum | BMNH-1939.2.11.3 |
| Phocidae | Male | British Natural History Museum | BMNH-1939.2.11.4 |
| Phocidae | Male | British Natural History Museum | BMNH-1939.2.11.8 |
| Phocidae | Male | British Natural History Museum | BMNH-1939.2.25.1 |
| Phocidae | Male | British Natural History Museum | BMNH-1939.2.25.3 |
| Phocidae | Male | British Natural History Museum | BMNH-1940.4.6.146 |
| Phocidae | Male | Museum National d'Histoire Naturelle | MNHN-1952-194 |
| Phocidae | Male | British Natural History Museum | BMNH-1958.4.25.7 |
| Phocidae | Male | British Natural History Museum | BMNH-1959.12.4.1 |
| Phocidae | Male | Museum National d'Histoire Naturelle | MNHN-1982-799 |
| Phocidae | Male | Zoologisch Museum Amsterdam | ZMA-24.204 |
| Phocidae | Unidentified | Museum fur Naturkunde | MNHB-12714 |
| Phocidae | Unidentified | Museum fur Naturkunde | MNHB-36253 |
| Phocidae | Unidentified | Museum fur Naturkunde | MNHB-38317 |
| Phocidae | Unidentified | Museum fur Naturkunde | MNHB-38321 |
| Phocidae | Unidentified | British Natural History Museum | BMNH-1843.1.8.4 |
| Phocidae | Unidentified | British Natural History Museum | BMNH-1846.4.15.23 |
| Phocidae | Unidentified | British Natural History Museum | BMNH-1846.4.15.24 |
| Phocidae | Unidentified | British Natural History Museum | BMNH-1884-1152 |
| Phocidae | Unidentified | British Natural History Museum | BMNH-1885.10.20.2 |
| Phocidae | Unidentified | British Natural History Museum | BMNH-1893.9.14.1 |
| Phocidae | Unidentified | Museum National d'Histoire Naturelle | MNHN-1955-173 |
| Phocidae | Unidentified | British Natural History Museum | BMNH-1958.4.24.7 |
| Phocidae | Unidentified | Museum National d'Histoire Naturelle | MNHN-1971-116 |
| Phocidae | Unidentified | Museum fur Naturkunde | MNHB-36250 |
| Phocidae | Unidentified | Grant Museum of Zoology - UCL | UCL-GMZ-Z1125 |
| Phocidae | Unidentified | Grant Museum of Zoology - UCL | UCL-GMZ-Z1646 |
| Phocidae | Female | Museum fur Naturkunde | MNHB-86629 |
| Phocidae | Female | British Natural History Museum | BMNH-1908.2.20.36 |
| Phocidae | Female | British Natural History Museum | BMNH-1908.2.20.41 |
| Phocidae | Female | British Natural History Museum | BMNH-1908.2.20.43 |
| Phocidae | Female | British Natural History Museum | BMNH-1935.3.29.4 |
| Phocidae | Female | British Natural History Museum | BMNH-1939.2.4.6 |
| Phocidae | Female | British Natural History Museum | BMNH-1939.2.4.8 |
| Phocidae | Female | British Natural History Museum | BMNH-1940.4.6.12 |
| Phocidae | Female | British Natural History Museum | BMNH-1940.4.6.129 |
| Phocidae | Female | British Natural History Museum | BMNH-1940.4.6.13 |
| Phocidae | Female | British Natural History Museum | BMNH-1940.4.6.132 |
| Phocidae | Female | British Natural History Museum | BMNH-1940.4.6.49 |
| Phocidae | Female | British Natural History Museum | BMNH-1940.4.6.67 |
| Phocidae | Female | British Natural History Museum | BMNH-1940.4.6.7 |
| Phocidae | Female | British Natural History Museum | BMNH-1940.4.6.86 |
| Phocidae | Female | British Natural History Museum | BMNH-1940.4.6.88 |
| Phocidae | Female | British Natural History Museum | BMNH-1940.4.6.90 |
| Phocidae | Female | British Natural History Museum | BMNH-1959.12.8.3 |
| Phocidae | Female | British Natural History Museum | BMNH-1959.12.8.5 |
| Phocidae | Female | British Natural History Museum | BMNH-1959.12.8.9 |
| Phocidae | Male | Museum fur Naturkunde | MNHB-86630 |
| Phocidae | Male | British Natural History Museum | BMNH-1908.2.20.34 |
| Phocidae | Male | British Natural History Museum | BMNH-1935.3.29.1 |
| Phocidae | Male | British Natural History Museum | BMNH-1939.2.4.1 |
| Phocidae | Male | British Natural History Museum | BMNH-1939.2.4.4 |
| Phocidae | Male | British Natural History Museum | BMNH-1939.2.4.7 |
| Phocidae | Male | British Natural History Museum | BMNH-1940.4.6.10 |
| Phocidae | Male | British Natural History Museum | BMNH-1940.4.6.58 |
| Phocidae | Male | British Natural History Museum | BMNH-1940.4.6.63 |
| Phocidae | Male | British Natural History Museum | BMNH-1940.4.6.77 |
| Phocidae | Male | British Natural History Museum | BMNH-1940.4.6.8 |
| Phocidae | Male | British Natural History Museum | BMNH-1940.4.6.85 |
| Phocidae | Male | British Natural History Museum | BMNH-1940.4.6.89 |
| Phocidae | Male | British Natural History Museum | BMNH-1940.4.6.92 |
| Phocidae | Male | British Natural History Museum | BMNH-1940.4.6.93 |
| Phocidae | Male | British Natural History Museum | BMNH-1949.4.6.8 |
| Phocidae | Male | British Natural History Museum | BMNH-1951.6.25.4 |
| Phocidae | Male | British Natural History Museum | BMNH-1956.12.8.6 |
| Phocidae | Male | British Natural History Museum | BMNH-1959.12.8.10 |
| Phocidae | Male | British Natural History Museum | BMNH-1959.12.8.18 |
| Phocidae | Male | British Natural History Museum | BMNH-1959.12.8.2 |
| Phocidae | Male | British Natural History Museum | BMNH-1959.12.8.7 |
| Phocidae | Male | British Natural History Museum | BMNH-1959.12.8.8 |
| Phocidae | Male | Museum National d'Histoire Naturelle | MNHN-1981-1323 |
| Phocidae | Male | Museum National d'Histoire Naturelle | MNHN-1981-1325 |
| Phocidae | Unidentified | Museum fur Naturkunde | MNHB-36258 |
| Phocidae | Unidentified | Museum fur Naturkunde | MNHB-36265 |
| Phocidae | Unidentified | Museum fur Naturkunde | MNHB-38325 |
| Phocidae | Unidentified | Museum fur Naturkunde | MNHB-38329 |
| Phocidae | Unidentified | British Natural History Museum | BMNH-1844.10.29.18 |
| Phocidae | Unidentified | British Natural History Museum | BMNH-1844.11.16.4 |
| Phocidae | Unidentified | British Natural History Museum | BMNH-1901.1.4.10 |
| Phocidae | Unidentified | British Natural History Museum | BMNH-1910.9.19.3 |
| Phocidae | Unidentified | British Natural History Museum | BMNH-1939.2.4.3 |
| Phocidae | Unidentified | Museum National d'Histoire Naturelle | MNHN-1939-4479 |
| Phocidae | Unidentified | British Natural History Museum | BMNH-1940.4.6.11 |
| Phocidae | Unidentified | British Natural History Museum | BMNH-1951.8.28.15 |
| Phocidae | Unidentified | Museum National d'Histoire Naturelle | MNHN-1981-1324 |
| Phocidae | Unidentified | Museum National d'Histoire Naturelle | MNHN-1981-1334 |
| Phocidae | Unidentified | Museum of Zoology Cambridge | MZC-K.7903 |
| Phocidae | Female | British Natural History Museum | BMNH-1908.2.20.11 |
| Phocidae | Female | British Natural History Museum | BMNH-1908.2.20.20 |
| Phocidae | Female | British Natural History Museum | BMNH-1908.2.20.21 |
| Phocidae | Female | British Natural History Museum | BMNH-1908.2.20.25 |
| Phocidae | Female | British Natural History Museum | BMNH-1908.2.20.62 |
| Phocidae | Female | British Natural History Museum | BMNH-1908.2.20.63 |
| Phocidae | Female | British Natural History Museum | BMNH-1940.4.6.21 |
| Phocidae | Female | British Natural History Museum | BMNH-1940.4.6.22 |
| Phocidae | Female | British Natural History Museum | BMNH-1940.4.6.23 |
| Phocidae | Female | British Natural History Museum | BMNH-1940.4.6.24 |
| Phocidae | Female | British Natural History Museum | BMNH-1940.4.6.25 |
| Phocidae | Female | British Natural History Museum | BMNH-1940.4.6.27 |
| Phocidae | Female | British Natural History Museum | BMNH-1940.4.6.32 |
| Phocidae | Female | British Natural History Museum | BMNH-1940.4.6.35 |
| Phocidae | Female | British Natural History Museum | BMNH-1940.4.6.44 |
| Phocidae | Female | British Natural History Museum | BMNH-1940.4.6.47 |
| Phocidae | Female | British Natural History Museum | BMNH-1940.4.6.65 |
| Phocidae | Female | British Natural History Museum | BMNH-1940.4.6.99 |
| Phocidae | Female | British Natural History Museum | BMNH-1959.12.16.13 |
| Phocidae | Female | British Natural History Museum | BMNH-1959.12.16.15 |
| Phocidae | Female | British Natural History Museum | BMNH-1959.12.16.3 |
| Phocidae | Female | British Natural History Museum | BMNH-1959.12.16.4 |
| Phocidae | Male | British Natural History Museum | BMNH-1908.2.20.13 |
| Phocidae | Male | British Natural History Museum | BMNH-1908.2.20.14 |
| Phocidae | Male | British Natural History Museum | BMNH-1908.2.20.16 |
| Phocidae | Male | British Natural History Museum | BMNH-1908.2.20.17 |
| Phocidae | Male | British Natural History Museum | BMNH-1908.2.20.18 |
| Phocidae | Male | British Natural History Museum | BMNH-1908.2.20.2 |
| Phocidae | Male | British Natural History Museum | BMNH-1908.2.20.22 |
| Phocidae | Male | British Natural History Museum | BMNH-1908.2.20.26 |
| Phocidae | Male | British Natural History Museum | BMNH-1908.2.20.59 |
| Phocidae | Male | British Natural History Museum | BMNH-1908.2.20.60 |
| Phocidae | Male | British Natural History Museum | BMNH-1940.4.6.108 |
| Phocidae | Male | British Natural History Museum | BMNH-1940.4.6.109 |
| Phocidae | Male | British Natural History Museum | BMNH-1940.4.6.110 |
| Phocidae | Male | British Natural History Museum | BMNH-1940.4.6.18 |
| Phocidae | Male | British Natural History Museum | BMNH-1940.4.6.72 |
| Phocidae | Male | British Natural History Museum | BMNH-1951.5.1.2 |
| Phocidae | Male | British Natural History Museum | BMNH-1959.12.16.2 |
| Phocidae | Male | British Natural History Museum | BMNH-1959.12.16.6 |
| Phocidae | Male | British Natural History Museum | BMNH-1959.12.16.8 |
| Phocidae | Male | British Natural History Museum | BMNH-1959.5.16.1 |
| Phocidae | Unidentified | Museum fur Naturkunde | MNHB-36268 |
| Phocidae | Unidentified | Museum fur Naturkunde | MNHB-36269 |
| Phocidae | Unidentified | Museum fur Naturkunde | MNHB-36270 |
| Phocidae | Unidentified | Museum fur Naturkunde | MNHB-36271 |
| Phocidae | Unidentified | Museum fur Naturkunde | MNHB-36274 |
| Phocidae | Unidentified | Museum fur Naturkunde | MNHB-36275 |
| Phocidae | Unidentified | Museum fur Naturkunde | MNHB-36278 |
| Phocidae | Unidentified | Museum fur Naturkunde | MNHB-36281 |
| Phocidae | Unidentified | Museum fur Naturkunde | MNHB-36282 |
| Phocidae | Unidentified | Museum fur Naturkunde | MNHB-36283 |
| Phocidae | Unidentified | Museum fur Naturkunde | MNHB-36284 |
| Phocidae | Unidentified | British Natural History Museum | BMNH-1846.4.25.22 |
| Phocidae | Unidentified | British Natural History Museum | BMNH-1908.2.20.23 |
| Phocidae | Unidentified | British Natural History Museum | BMNH-1908.2.20.58 |
| Phocidae | Unidentified | British Natural History Museum | BMNH-1951.5.1.4 |
| Phocidae | Unidentified | British Natural History Museum | BMNH-1951.5.1.6 |
| Phocidae | Unidentified | British Natural History Museum | BMNH-1951.5.1.7 |
| Phocidae | Unidentified | British Natural History Museum | BMNH-1951.5.1.7 |
| Phocidae | Unidentified | British Natural History Museum | BMNH-1951.5.16.5 |
| Phocidae | Unidentified | British Natural History Museum | BMNH-1951.5.8.3 |
| Phocidae | Unidentified | British Natural History Museum | BMNH-1951.5.8.4 |
| Phocidae | Unidentified | British Natural History Museum | BMNH-1951.5.8.6 |
| Phocidae | Unidentified | British Natural History Museum | BMNH-1951.8.28.16 |
| Phocidae | Unidentified | British Natural History Museum | BMNH-1951.8.28.17 |
| Phocidae | Unidentified | British Natural History Museum | BMNH-1959.8.28.18 |
| Phocidae | Unidentified | Museum National d'Histoire Naturelle | MNHN-1981-1787 |
| Phocidae | Unidentified | Grant Museum of Zoology – UCL | UCL-GMZ-Z1126 |
| Phocidae | Unidentified | Grant Museum of Zoology – UCL | UCL-GMZ-Z1127 |
| Phocidae | Female | British Natural History Museum | BMNH-1951.7.17.5 |
| Phocidae | Female | British Natural History Museum | BMNH-1954.5.20.13 |
| Phocidae | Female | British Natural History Museum | BMNH-1954.5.20.21 |
| Phocidae | Female | British Natural History Museum | BMNH-1954.5.20.23 |
| Phocidae | Female | British Natural History Museum | BMNH-1954.5.20.4 |
| Phocidae | Female | British Natural History Museum | BMNH-1954.5.20.52 |
| Phocidae | Female | British Natural History Museum | BMNH-1954.5.20.55 |
| Phocidae | Female | British Natural History Museum | BMNH-1954.5.20.61 |
| Phocidae | Female | British Natural History Museum | BMNH-1954.5.20.8 |
| Phocidae | Female | British Natural History Museum | BMNH-1954.5.20.9 |
| Phocidae | Female | British Natural History Museum | BMNH-1959.12.17.3 |
| Phocidae | Female | British Natural History Museum | BMNH-867 |
| Phocidae | Male | British Natural History Museum | BMNH-1843.11.16.25 |
| Phocidae | Male | British Natural History Museum | BMNH-1894.11.17.1 |
| Phocidae | Male | British Natural History Museum | BMNH-1933.8.12.1 |
| Phocidae | Male | British Natural History Museum | BMNH-1939.4.29.1 |
| Phocidae | Male | British Natural History Museum | BMNH-1939.5.20.1 |
| Phocidae | Male | British Natural History Museum | BMNH-1949.2.1.3 |
| Phocidae | Male | British Natural History Museum | BMNH-1949.2.1.4 |
| Phocidae | Male | British Natural History Museum | BMNH-1949.2.3.11 |
| Phocidae | Male | British Natural History Museum | BMNH-1951.7.17.7 |
| Phocidae | Male | British Natural History Museum | BMNH-1951.7.17.8 |
| Phocidae | Male | British Natural History Museum | BMNH-1954.5.20.32 |
| Phocidae | Male | British Natural History Museum | BMNH-1954.5.20.33 |
| Phocidae | Male | British Natural History Museum | BMNH-1954.5.20.34 |
| Phocidae | Male | British Natural History Museum | BMNH-1954.5.20.64 |
| Phocidae | Male | British Natural History Museum | BMNH-1955.11.24.3 |
| Phocidae | Male | British Natural History Museum | BMNH-1994.11.17.1 |
| Phocidae | Male | Zoologisch Museum Amsterdam | ZMA-23.394 |
| Phocidae | Male | University of Edinburgh | UE-NHC-XH13.6.1 |
| Phocidae | Female | British Natural History Museum | BNHM-1938.12.10.1 |
| Phocidae | Female | Zoologisch Museum Amsterdam | ZMA-20.406 |
| Phocidae | Female | Zoologisch Museum Amsterdam | ZMA-20.413 |
| Phocidae | Female | Zoologisch Museum Amsterdam | ZMA-24.324 |
| Phocidae | Female | Zoologisch Museum Amsterdam | ZMA-24.325 |
| Phocidae | Female | Zoologisch Museum Amsterdam | ZMA-24.326 |
| Phocidae | Female | Zoologisch Museum Amsterdam | ZMA-24.331 |
| Phocidae | Female | Zoologisch Museum Amsterdam | ZMA-24.348 |
| Phocidae | Female | Zoologisch Museum Amsterdam | ZMA-24.364 |
| Phocidae | Female | Zoologisch Museum Amsterdam | ZMA-24.367 |
| Phocidae | Female | Zoologisch Museum Amsterdam | ZMA-24.694 |
| Phocidae | Male | Zoologisch Museum Amsterdam | ZMA-16.851 |
| Phocidae | Male | Zoologisch Museum Amsterdam | ZMA-16.852 |
| Phocidae | Male | British Natural History Museum | BMNH-1938.12.10.2 |
| Phocidae | Male | British Natural History Museum | BMNH-1951.11.28.2 |
| Phocidae | Male | British Natural History Museum | BMNH-1963.7.19.1 |
| Phocidae | Male | British Natural History Museum | BMNH-1963.7.19.3 |
| Phocidae | Male | British Natural History Museum | BMNH-1963.7.19.4 |
| Phocidae | Male | British Natural History Museum | BMNH-1963.7.19.5 |
| Phocidae | Male | Zoologisch Museum Amsterdam | ZMA-20.229 |
| Phocidae | Male | Zoologisch Museum Amsterdam | ZMA-20.301 |
| Phocidae | Male | Zoologisch Museum Amsterdam | ZMA-20.323 |
| Phocidae | Male | Zoologisch Museum Amsterdam | ZMA-20.410 |
| Phocidae | Male | Zoologisch Museum Amsterdam | ZMA-23.233 |
| Phocidae | Male | Zoologisch Museum Amsterdam | ZMA-23.483 |
| Phocidae | Male | Zoologisch Museum Amsterdam | ZMA-24.327 |
| Phocidae | Male | Zoologisch Museum Amsterdam | ZMA-24.334 |
| Phocidae | Male | Zoologisch Museum Amsterdam | ZMA-24.344 |
| Phocidae | Male | Zoologisch Museum Amsterdam | ZMA-24.347 |
| Phocidae | Male | Zoologisch Museum Amsterdam | ZMA-24.382 |
| Phocidae | Unidentified | British Natural History Museum | BNHM-1843.6.23.5 |
| Phocidae | Unidentified | British Natural History Museum | BNHM-1843.6.23.6 |
| Phocidae | Unidentified | British Natural History Museum | BNHM-1843.6.23.7 |
| Phocidae | Unidentified | British Natural History Museum | BNHM-1855.11.26.33 |
| Phocidae | Unidentified | Museum National d'Histoire Naturelle | MNHN-1865-509 |
| Phocidae | Unidentified | British Natural History Museum | BNHM-1919.7.7.3262 |
| Phocidae | Unidentified | British Natural History Museum | BNHM-1938.12.10.3 |
| Phocidae | Unidentified | Museum National d'Histoire Naturelle | MNHN-1985-2007 |
| Phocidae | Unidentified | Zoologisch Museum Amsterdam | ZMA-699 |
| Phocidae | Unidentified | Zoologisch Museum Amsterdam | ZMA-890 |
| Phocidae | Unidentified | Zoologisch Museum Amsterdam | ZMA-893 |
| Phocidae | Unidentified | Museum of Zoology Cambridge | MZC-K.8281 |
| Phocidae | Unidentified | Museum of Zoology Cambridge | MZC-K.8282 |
| Phocidae | Unidentified | Museum of Zoology Cambridge | MZC-K.8283 |
| Phocidae | Unidentified | Museum of Zoology Cambridge | MZC-K.8284 |
| Phocidae | Unidentified | Museum of Zoology Cambridge | MZC-K.8285 |
| Phocidae | Unidentified | Museum National d'Histoire Naturelle | MNHN-1985-2010 |
| Phocidae | Female | Zoologisch Museum Amsterdam | ZMA-23.558 |
| Phocidae | Female | Zoologisch Museum Amsterdam | ZMA-23.559 |
| Phocidae | Female | Zoologisch Museum Amsterdam | ZMA-23.560 |
| Phocidae | Female | Zoologisch Museum Amsterdam | ZMA-23.562 |
| Phocidae | Female | Zoologisch Museum Amsterdam | ZMA-23.716 |
| Phocidae | Female | Zoologisch Museum Amsterdam | ZMA-23.717 |
| Phocidae | Female | Zoologisch Museum Amsterdam | ZMA-23.722 |
| Phocidae | Female | Zoologisch Museum Amsterdam | ZMA-23.735 |
| Phocidae | Female | Zoologisch Museum Amsterdam | ZMA-23.759 |
| Phocidae | Female | Zoologisch Museum Amsterdam | ZMA-23.817 |
| Phocidae | Female | Zoologisch Museum Amsterdam | ZMA-23.819 |
| Phocidae | Female | Zoologisch Museum Amsterdam | ZMA-23.823 |
| Phocidae | Female | Zoologisch Museum Amsterdam | ZMA-23.824 |
| Phocidae | Female | Zoologisch Museum Amsterdam | ZMA-73.713 |
| Phocidae | Male | Zoologisch Museum Amsterdam | ZMA.72453 |
| Phocidae | Male | British Natural History Museum | BNHM-1868.3.21.1 |
| Phocidae | Male | Zoologisch Museum Amsterdam | ZMA-23.556 |
| Phocidae | Male | Zoologisch Museum Amsterdam | ZMA-23.561 |
| Phocidae | Male | Zoologisch Museum Amsterdam | ZMA-23.718 |
| Phocidae | Male | Zoologisch Museum Amsterdam | ZMA-23.721 |
| Phocidae | Male | Zoologisch Museum Amsterdam | ZMA-23.725 |
| Phocidae | Male | Zoologisch Museum Amsterdam | ZMA-23.726 |
| Phocidae | Male | Zoologisch Museum Amsterdam | ZMA-23.758 |
| Phocidae | Male | Zoologisch Museum Amsterdam | ZMA-23.760 |
| Phocidae | Male | Zoologisch Museum Amsterdam | ZMA-23.771 |
| Phocidae | Male | Zoologisch Museum Amsterdam | ZMA-3 |
| Phocidae | Male | Zoologisch Museum Amsterdam | ZMA-73.712 |
| Phocidae | Male | Zoologisch Museum Amsterdam | ZMA-K.8089 |
| Phocidae | Unidentified | Museum fur Naturkunde | MNHB-72451 |
| Phocidae | Unidentified | Museum fur Naturkunde | MNHB-72454 |
| Phocidae | Unidentified | Museum fur Naturkunde | MNHB-72455 |
| Phocidae | Unidentified | Museum fur Naturkunde | MNHB-85866 |
| Phocidae | Unidentified | British Natural History Museum | BNHM-1919.7.7.3260 |
| Phocidae | Unidentified | British Natural History Museum | BNHM-1928.9.1.2 |
| Phocidae | Unidentified | British Natural History Museum | BNHM-1951.3.2.1 |
| Phocidae | Unidentified | Museum National d'Histoire Naturelle | MNHN-2007-411 |
| Phocidae | Unidentified | Museum of Zoology Cambridge | MZC-K.8081 |
| Phocidae | Unidentified | Museum of Zoology Cambridge | MZC-K.8087 |
| Phocidae | Unidentified | Museum of Zoology Cambridge | MZC-K.8088 |
| Phocidae | Unidentified | Museum of Zoology Cambridge | MZC-K.8092 |
| Phocidae | Unidentified | Museum of Zoology Cambridge | MZC-K.8173 |
| Phocidae | Unidentified | Museum of Zoology Cambridge | MZC-K.8191 |
| Phocidae | Unidentified | Grant Museum of Zoology – UCL | UCL-GMZ-Z2250 |
| Phocidae | Unidentified | University of Edinburgh | UE-NHC-XH13-2.3 |
| Phocidae | Unidentified | University of Edinburgh | UE-NHC-XH13-2.3a |
