# Supplementary material for: Shifts in cranial integration associated with ecological specialization in pinnipeds (Mammalia, Carnivora)
Source: R Soc Open Sci. 2019 Mar 27;6(3):190201. doi: 10.1098/rsos.190201 (PMC6458409; doi:10.1098/rsos.190201)
Supplement: Table S2 [file rsos190201supp2.docx]

Table S2: Results for the modularity analyses for individual species, before and after relevant allometric corrections (see main text):

| **Odobenidae** |  |  |  |  |  |  |  |  |
| --- | --- | --- | --- | --- | --- | --- | --- | --- |
| *Odobenus rosmarus* |  |  |  |  |  |  |  |  |
|  | MaxL | K | n | AICc | dAICc | Model_L | Post_Pob |  |
| clusters.sep.Mod + sep.between | 1089.872 | 22 | 703 | -2134.256 | 28.850 | 0.000 | 0.000 |  |
| clusters.same.Mod + sep.between | 1087.454 | 17 | 703 | -2140.014 | 23.091 | 0.000 | 0.000 |  |
| Function.sep.Mod + sep.between | 1086.859 | 11 | 703 | -2151.336 | 11.770 | 0.003 | 0.001 |  |
| Function.sep.Mod + same.between | 1083.125 | 6 | 703 | -2154.130 | 8.976 | 0.011 | 0.005 |  |
| Function.same.Mod + sep.between | 1085.518 | 8 | 703 | -2154.828 | 8.277 | 0.016 | 0.007 |  |
| clusters.sep.Mod + same.between | 1085.687 | 8 | 703 | -2155.167 | 7.938 | 0.019 | 0.009 |  |
| ossification.sep.Mod + same.between | 1082.464 | 4 | 703 | -2156.870 | 6.236 | 0.044 | 0.021 |  |
| ossification.same.Mod + same.between | 1081.777 | 3 | 703 | -2157.520 | 5.585 | 0.061 | 0.029 |  |
| Function.same.Mod + same.between | 1081.784 | 3 | 703 | -2157.534 | 5.571 | 0.062 | 0.029 |  |
| Tissue.origin.sep.Mod + same.between | 1082.823 | 4 | 703 | -2157.588 | 5.517 | 0.063 | 0.030 |  |
| No.modules.default | 1081.021 | 2 | 703 | -2158.025 | 5.080 | 0.079 | 0.037 |  |
| Tissue.origin.same.Mod + same.between | 1082.328 | 3 | 703 | -2158.621 | 4.484 | 0.106 | 0.050 |  |
| clusters.same.Mod + same.between | 1083.269 | 3 | 703 | -2160.504 | 2.602 | 0.272 | 0.128 |  |
| front.back.same.Mod + same.between | 1083.642 | 3 | 703 | -2161.249 | 1.856 | 0.395 | 0.185 |  |
| front.back.sep.Mod + same.between | 1085.581 | 4 | 703 | -2163.105 | 0.000 | 1.000 | 0.469 |  |
|  |  |  |  |  |  |  |  |  |
| front.back.sep.Mod + same.between |  |  |  |  |  |  |  |  |
|  | Module 1 | Module 2 | betweenModules | unintegrated |  |  |  |  |
| MaxL | 237.865 | 267.458 | 580.259 | 0.000 |  |  |  |  |
| MaxL_p | 0.170 | 0.130 | 0.120 | 0.000 |  |  |  |  |
|  |  |  |  |  |  |  |  |  |
| front.back.same.Mod + same.between |  |  |  |  |  |  |  |  |
|  | withinModules | betweenModules | unintegrated |  |  |  |  |  |
| MaxL | 503.383 | 580.259 | 0.000 |  |  |  |  |  |
| MaxL_p | 0.150 | 0.120 | 0.000 |  |  |  |  |  |
|  |  |  |  |  |  |  |  |  |
| clusters.same.Mod + same.between |  |  |  |  |  |  |  |  |
|  | withinModules | betweenModules | unintegrated |  |  |  |  |  |
| MaxL | 159.201 | 924.068 | 0.000 |  |  |  |  |  |
| MaxL_p | 0.160 | 0.130 | 0.000 |  |  |  |  |  |
|  |  |  |  |  |  |  |  |  |
| **After corrections:** |  |  |  |  |  |  |  |  |
|  | MaxL | K | n | AICc | dAICc | Model_L | Post_Pob |  |
| clusters.sep.Mod + sep.between | 1101.487 | 22.000 | 703.000 | -2157.486 | 26.490 | 0.000 | 0.000 |  |
| clusters.same.Mod + sep.between | 1099.974 | 17.000 | 703.000 | -2165.055 | 18.922 | 0.000 | 0.000 |  |
| Function.sep.Mod + sep.between | 1096.234 | 11.000 | 703.000 | -2170.086 | 13.890 | 0.001 | 0.000 |  |
| ossification.sep.Mod + same.between | 1090.597 | 4.000 | 703.000 | -2173.136 | 10.840 | 0.004 | 0.002 |  |
| Function.sep.Mod + same.between | 1093.043 | 6.000 | 703.000 | -2173.966 | 10.011 | 0.007 | 0.003 |  |
| Tissue.origin.sep.Mod + same.between | 1091.412 | 4.000 | 703.000 | -2174.766 | 9.210 | 0.010 | 0.005 |  |
| Function.same.Mod + sep.between | 1095.665 | 8.000 | 703.000 | -2175.123 | 8.853 | 0.012 | 0.006 |  |
| ossification.same.Mod + same.between | 1090.597 | 3.000 | 703.000 | -2175.159 | 8.817 | 0.012 | 0.006 |  |
| No.modules.default | 1090.282 | 2.000 | 703.000 | -2176.547 | 7.430 | 0.024 | 0.012 |  |
| Tissue.origin.same.Mod + same.between | 1091.397 | 3.000 | 703.000 | -2176.759 | 7.217 | 0.027 | 0.014 |  |
| clusters.sep.Mod + same.between | 1096.519 | 8.000 | 703.000 | -2176.830 | 7.147 | 0.028 | 0.014 |  |
| Function.same.Mod + same.between | 1092.474 | 3.000 | 703.000 | -2178.914 | 5.062 | 0.080 | 0.041 |  |
| front.back.sep.Mod + same.between | 1094.820 | 4.000 | 703.000 | -2181.583 | 2.394 | 0.302 | 0.154 |  |
| front.back.same.Mod + same.between | 1094.204 | 3.000 | 703.000 | -2182.373 | 1.603 | 0.449 | 0.229 |  |
| clusters.same.Mod + same.between | 1095.005 | 3.000 | 703.000 | -2183.976 | 0.000 | 1.000 | 0.511 |  |
|  |  |  |  |  |  |  |  |  |
| front.back.sep.Mod + same.between |  |  |  |  |  |  |  |  |
|  | Module 1 | Module 2 | betweenModules | unintegrated |  |  |  |  |
| MaxL | 233.217 | 273.619 | 587.984 | 0.000 |  |  |  |  |
| MaxL_p | 0.150 | 0.130 | 0.100 | 0.000 |  |  |  |  |
|  |  |  |  |  |  |  |  |  |
| front.back.same.Mod + same.between |  |  |  |  |  |  |  |  |
|  | withinModules | betweenModules | unintegrated |  |  |  |  |  |
| MaxL | 506.220 | 587.984 | 0.000 |  |  |  |  |  |
| MaxL_p | 0.140 | 0.100 | 0.000 |  |  |  |  |  |
|  |  |  |  |  |  |  |  |  |
| clusters.same.Mod + same.between |  |  |  |  |  |  |  |  |
|  | withinModules | betweenModules | unintegrated |  |  |  |  |  |
| MaxL | 156.951 | 938.054 | 0.000 |  |  |  |  |  |
| MaxL_p | 0.160 | 0.110 | 0.000 |  |  |  |  |  |
|  |  |  |  |  |  |  |  |  |
| **Otaridae** |  |  |  |  |  |  |  |  |
| ***Arctocephalus australis*** |  |  |  |  |  |  |  |  |
| **Before corrections:** |  |  |  |  |  |  |  |  |
|  | MaxL | K | n | AICc | dAICc | Model_L | Post_Pob |  |
| No.modules.default | 1108.171 | 2.000 | 703.000 | -2212.325 | 24.120 | 0.000 | 0.000 |  |
| ossification.same.Mod + same.between | 1110.452 | 3.000 | 703.000 | -2214.869 | 21.576 | 0.000 | 0.000 |  |
| Function.sep.Mod + sep.between | 1118.870 | 11.000 | 703.000 | -2215.358 | 21.087 | 0.000 | 0.000 |  |
| ossification.sep.Mod + same.between | 1112.095 | 4.000 | 703.000 | -2216.133 | 20.312 | 0.000 | 0.000 |  |
| Tissue.origin.same.Mod + same.between | 1111.368 | 3.000 | 703.000 | -2216.702 | 19.743 | 0.000 | 0.000 |  |
| Function.sep.Mod + same.between | 1115.467 | 6.000 | 703.000 | -2218.813 | 17.633 | 0.000 | 0.000 |  |
| Tissue.origin.sep.Mod + same.between | 1113.572 | 4.000 | 703.000 | -2219.086 | 17.360 | 0.000 | 0.000 |  |
| Function.same.Mod + sep.between | 1118.186 | 8.000 | 703.000 | -2220.164 | 16.282 | 0.000 | 0.000 |  |
| clusters.same.Mod + sep.between | 1128.162 | 17.000 | 703.000 | -2221.431 | 15.014 | 0.001 | 0.000 |  |
| Function.same.Mod + same.between | 1114.782 | 3.000 | 703.000 | -2223.530 | 12.916 | 0.002 | 0.001 |  |
| clusters.sep.Mod + sep.between | 1135.833 | 22.000 | 703.000 | -2226.178 | 10.268 | 0.006 | 0.005 |  |
| front.back.sep.Mod + same.between | 1117.693 | 4.000 | 703.000 | -2227.328 | 9.117 | 0.010 | 0.009 |  |
| front.back.same.Mod + same.between | 1117.365 | 3.000 | 703.000 | -2228.697 | 7.749 | 0.021 | 0.019 |  |
| clusters.same.Mod + same.between | 1118.656 | 3.000 | 703.000 | -2231.278 | 5.168 | 0.075 | 0.068 |  |
| clusters.sep.Mod + same.between | 1126.327 | 8.000 | 703.000 | -2236.446 | 0.000 | 1.000 | 0.896 |  |
|  |  |  |  |  |  |  |  |  |
|  |  |  |  |  |  |  |  |  |
| clusters.same.Mod + same.between |  |  |  |  |  |  |  |  |
|  | withinModules | betweenModules | unintegrated |  |  |  |  |  |
| MaxL | 149.669 | 968.987 | 0.000 |  |  |  |  |  |
| MaxL_p | 0.190 | 0.120 | 0.000 |  |  |  |  |  |
|  |  |  |  |  |  |  |  |  |
| clusters.sep.Mod + same.between |  |  |  |  |  |  |  |  |
|  | Module 1 | Module 2 | Module 3 | Module 6 | Module 4 | Module 5 | betweenModules | unintegrated |
| MaxL | 34.699 | 5.460 | 37.199 | 67.633 | 1.540 | 10.808 | 968.987 | 0.000 |
| MaxL_p | 0.210 | 0.170 | 0.170 | 0.150 | 0.420 | 0.270 | 0.120 | 0.000 |
|  |  |  |  |  |  |  |  |  |
| **After corrections:** |  |  |  |  |  |  |  |  |
|  | MaxL | K | n | AICc | dAICc | Model_L | Post_Pob |  |
| No.modules.default | 1136.412 | 2.000 | 703.000 | -2268.808 | 19.541 | 0.000 | 0.000 |  |
| clusters.same.Mod + sep.between | 1152.041 | 17.000 | 703.000 | -2269.188 | 19.161 | 0.000 | 0.000 |  |
| ossification.same.Mod + same.between | 1137.686 | 3.000 | 703.000 | -2269.338 | 19.011 | 0.000 | 0.000 |  |
| ossification.sep.Mod + same.between | 1139.025 | 4.000 | 703.000 | -2269.993 | 18.356 | 0.000 | 0.000 |  |
| Tissue.origin.same.Mod + same.between | 1138.783 | 3.000 | 703.000 | -2271.533 | 16.816 | 0.000 | 0.000 |  |
| clusters.sep.Mod + sep.between | 1158.900 | 22.000 | 703.000 | -2272.312 | 16.037 | 0.000 | 0.000 |  |
| Tissue.origin.sep.Mod + same.between | 1140.555 | 4.000 | 703.000 | -2273.052 | 15.297 | 0.000 | 0.000 |  |
| Function.sep.Mod + sep.between | 1148.099 | 11.000 | 703.000 | -2273.816 | 14.533 | 0.001 | 0.000 |  |
| Function.sep.Mod + same.between | 1143.536 | 6.000 | 703.000 | -2274.952 | 13.397 | 0.001 | 0.001 |  |
| Function.same.Mod + sep.between | 1146.920 | 8.000 | 703.000 | -2277.633 | 10.715 | 0.005 | 0.003 |  |
| Function.same.Mod + same.between | 1142.358 | 3.000 | 703.000 | -2278.681 | 9.668 | 0.008 | 0.005 |  |
| front.back.sep.Mod + same.between | 1145.564 | 4.000 | 703.000 | -2283.070 | 5.279 | 0.071 | 0.049 |  |
| clusters.same.Mod + same.between | 1145.419 | 3.000 | 703.000 | -2284.803 | 3.545 | 0.170 | 0.117 |  |
| front.back.same.Mod + same.between | 1145.564 | 3.000 | 703.000 | -2285.093 | 3.256 | 0.196 | 0.135 |  |
| clusters.sep.Mod + same.between | 1152.278 | 8.000 | 703.000 | -2288.349 | 0.000 | 1.000 | 0.688 |  |
|  |  |  |  |  |  |  |  |  |
| front.back.same.Mod + same.between |  |  |  |  |  |  |  |  |
|  | withinModules | betweenModules | unintegrated |  |  |  |  |  |
| MaxL | 521.034 | 624.530 | 0.000 |  |  |  |  |  |
| MaxL_p | 0.150 | 0.100 | 0.000 |  |  |  |  |  |
|  |  |  |  |  |  |  |  |  |
| clusters.same.Mod + same.between |  |  |  |  |  |  |  |  |
|  | withinModules | betweenModules | unintegrated |  |  |  |  |  |
| MaxL | 157.494 | 987.925 | 0.000 |  |  |  |  |  |
| MaxL_p | 0.180 | 0.110 | 0.000 |  |  |  |  |  |
|  |  |  |  |  |  |  |  |  |
| clusters.sep.Mod + same.between |  |  |  |  |  |  |  |  |
|  | Module 1 | Module 2 | Module 3 | Module 6 | Module 4 | Module 5 | betweenModules | unintegrated |
| MaxL | 34.459 | 5.334 | 37.174 | 69.696 | 3.013 | 14.677 | 987.925 | 0.000 |
| MaxL_p | 0.200 | 0.110 | 0.170 | 0.140 | 0.410 | 0.230 | 0.110 | 0.000 |
|  |  |  |  |  |  |  |  |  |
| ***Arctocephalus gazella*** |  |  |  |  |  |  |  |  |
| **Before corrections:** |  |  |  |  |  |  |  |  |
|  | MaxL | K | n | AICc | dAICc | Model_L | Post_Pob |  |
| No.modules.default | 903.451 | 2.000 | 703.000 | -1802.885 | 50.892 | 0.000 | 0.000 |  |
| Function.same.Mod + same.between | 904.068 | 3.000 | 703.000 | -1802.102 | 51.675 | 0.000 | 0.000 |  |
| Function.sep.Mod + same.between | 912.413 | 6.000 | 703.000 | -1812.705 | 41.072 | 0.000 | 0.000 |  |
| Function.same.Mod + sep.between | 912.111 | 8.000 | 703.000 | -1808.014 | 45.763 | 0.000 | 0.000 |  |
| Function.sep.Mod + sep.between | 920.456 | 11.000 | 703.000 | -1818.529 | 35.248 | 0.000 | 0.000 |  |
| Tissue.origin.same.Mod + same.between | 903.451 | 3.000 | 703.000 | -1800.868 | 52.909 | 0.000 | 0.000 |  |
| Tissue.origin.sep.Mod + same.between | 904.678 | 4.000 | 703.000 | -1801.299 | 52.478 | 0.000 | 0.000 |  |
| ossification.same.Mod + same.between | 903.451 | 3.000 | 703.000 | -1800.868 | 52.909 | 0.000 | 0.000 |  |
| ossification.sep.Mod + same.between | 903.982 | 4.000 | 703.000 | -1799.907 | 53.870 | 0.000 | 0.000 |  |
| front.back.same.Mod + same.between | 905.160 | 3.000 | 703.000 | -1804.285 | 49.492 | 0.000 | 0.000 |  |
| front.back.sep.Mod + same.between | 910.380 | 4.000 | 703.000 | -1812.702 | 41.075 | 0.000 | 0.000 |  |
| clusters.same.Mod + same.between | 905.620 | 3.000 | 703.000 | -1805.205 | 48.572 | 0.000 | 0.000 |  |
| clusters.sep.Mod + same.between | 920.586 | 8.000 | 703.000 | -1824.964 | 28.813 | 0.000 | 0.000 |  |
| clusters.same.Mod + sep.between | 934.667 | 17.000 | 703.000 | -1834.440 | 19.337 | 0.000 | 0.000 |  |
| clusters.sep.Mod + sep.between | 949.633 | 22.000 | 703.000 | -1853.777 | 0.000 | 1.000 | 1.000 |  |
|  |  |  |  |  |  |  |  |  |
| clusters.sep.Mod + sep.between |  |  |  |  |  |  |  |  |
|  | Module 1 | Module 2 | Module 3 | Module 6 | Module 4 | Module 5 | 1 to 2 | 1 to 3 |
| MaxL | 33.003 | 3.095 | 43.964 | 48.841 | 4.330 | -2.429 | 40.642 | 105.337 |
| MaxL_p | 0.200 | 0.470 | 0.170 | 0.290 | 0.220 | 0.330 | 0.310 | 0.180 |
|  | 1 to 6 | 1 to 4 | 1 to 5 | 2 to 3 | 2 to 6 | 2 to 4 | 2 to 5 | 3 to 6 |
|  | 113.645 | 35.706 | 58.183 | 37.688 | 31.447 | 15.143 | 21.012 | 125.022 |
|  | 0.230 | 0.180 | 0.200 | 0.240 | 0.350 | 0.180 | 0.270 | 0.200 |
|  | 3 to 4 | 3 to 5 | 6 to 4 | 6 to 5 | 4 to 5 | unintegrated |  |  |
|  | 37.550 | 78.265 | 36.785 | 60.062 | 22.344 | 0.000 |  |  |
|  | 0.200 | 0.180 | 0.210 | 0.260 | 0.220 | 0.000 |  |  |
| **After corrections:** |  |  |  |  |  |  |  |  |
|  | MaxL | K | n | AICc | dAICc | Model_L | Post_Pob |  |
| No.modules.default | 894.658 | 2.000 | 703.000 | -1785.300 | 89.862 | 0.000 | 0.000 |  |
| Function.same.Mod + same.between | 897.345 | 3.000 | 703.000 | -1788.655 | 86.506 | 0.000 | 0.000 |  |
| Function.sep.Mod + same.between | 911.447 | 6.000 | 703.000 | -1810.774 | 64.387 | 0.000 | 0.000 |  |
| Function.same.Mod + sep.between | 911.884 | 8.000 | 703.000 | -1807.561 | 67.600 | 0.000 | 0.000 |  |
| Function.sep.Mod + sep.between | 925.987 | 11.000 | 703.000 | -1829.591 | 45.570 | 0.000 | 0.000 |  |
| Tissue.origin.same.Mod + same.between | 895.717 | 3.000 | 703.000 | -1785.399 | 89.762 | 0.000 | 0.000 |  |
| Tissue.origin.sep.Mod + same.between | 897.277 | 4.000 | 703.000 | -1786.496 | 88.665 | 0.000 | 0.000 |  |
| ossification.same.Mod + same.between | 895.446 | 3.000 | 703.000 | -1784.857 | 90.304 | 0.000 | 0.000 |  |
| ossification.sep.Mod + same.between | 896.306 | 4.000 | 703.000 | -1784.555 | 90.606 | 0.000 | 0.000 |  |
| front.back.same.Mod + same.between | 903.802 | 3.000 | 703.000 | -1801.570 | 73.591 | 0.000 | 0.000 |  |
| front.back.sep.Mod + same.between | 916.596 | 4.000 | 703.000 | -1825.135 | 50.026 | 0.000 | 0.000 |  |
| clusters.same.Mod + same.between | 898.768 | 3.000 | 703.000 | -1791.502 | 83.659 | 0.000 | 0.000 |  |
| clusters.sep.Mod + same.between | 917.050 | 8.000 | 703.000 | -1817.892 | 57.269 | 0.000 | 0.000 |  |
| clusters.same.Mod + sep.between | 942.043 | 17.000 | 703.000 | -1849.193 | 25.968 | 0.000 | 0.000 |  |
| clusters.sep.Mod + sep.between | 960.325 | 22.000 | 703.000 | -1875.161 | 0.000 | 1.000 | 1.000 |  |
|  |  |  |  |  |  |  |  |  |
| clusters.sep.Mod + sep.between |  |  |  |  |  |  |  |  |
|  | Module 1 | Module 2 | Module 3 | Module 6 | Module 4 | Module 5 | 1 to 2 | 1 to 3 |
| MaxL | 22.552 | 4.872 | 45.919 | 50.994 | 5.565 | 5.478 | 36.205 | 104.414 |
| MaxL_p | 0.260 | 0.510 | 0.190 | 0.340 | 0.430 | 0.350 | 0.370 | 0.210 |
|  | 1 to 6 | 1 to 4 | 1 to 5 | 2 to 3 | 2 to 6 | 2 to 4 | 2 to 5 | 3 to 6 |
|  | 109.338 | 41.738 | 53.336 | 37.371 | 34.544 | 15.358 | 17.073 | 122.174 |
|  | 0.250 | 0.230 | 0.230 | 0.270 | 0.360 | 0.290 | 0.330 | 0.220 |
|  | 3 to 4 | 3 to 5 | 6 to 4 | 6 to 5 | 4 to 5 | unintegrated |  |  |
|  | 41.919 | 74.887 | 42.491 | 64.418 | 29.676 | 0.000 |  |  |
|  | 0.250 | 0.190 | 0.320 | 0.330 | 0.260 | 0.000 |  |  |
|  |  |  |  |  |  |  |  |  |
|  |  |  |  |  |  |  |  |  |
| ***Arctocephalus tropicalis*** |  |  |  |  |  |  |  |  |
| **Before corrections:** |  |  |  |  |  |  |  |  |
|  | MaxL | K | n | AICc | dAICc | Model_L | Post_Pob |  |
| No.modules.default | 927.738 | 2.000 | 703.000 | -1851.459 | 48.060 | 0.000 | 0.000 |  |
| Function.same.Mod + same.between | 932.373 | 3.000 | 703.000 | -1858.713 | 40.806 | 0.000 | 0.000 |  |
| Function.sep.Mod + same.between | 933.851 | 6.000 | 703.000 | -1855.582 | 43.937 | 0.000 | 0.000 |  |
| Function.same.Mod + sep.between | 938.956 | 8.000 | 703.000 | -1861.705 | 37.814 | 0.000 | 0.000 |  |
| Function.sep.Mod + sep.between | 940.434 | 11.000 | 703.000 | -1858.486 | 41.033 | 0.000 | 0.000 |  |
| Tissue.origin.same.Mod + same.between | 928.961 | 3.000 | 703.000 | -1851.888 | 47.631 | 0.000 | 0.000 |  |
| Tissue.origin.sep.Mod + same.between | 930.394 | 4.000 | 703.000 | -1852.731 | 46.788 | 0.000 | 0.000 |  |
| ossification.same.Mod + same.between | 928.583 | 3.000 | 703.000 | -1851.132 | 48.387 | 0.000 | 0.000 |  |
| ossification.sep.Mod + same.between | 928.602 | 4.000 | 703.000 | -1849.146 | 50.373 | 0.000 | 0.000 |  |
| front.back.same.Mod + same.between | 933.263 | 3.000 | 703.000 | -1860.491 | 39.028 | 0.000 | 0.000 |  |
| front.back.sep.Mod + same.between | 937.680 | 4.000 | 703.000 | -1867.302 | 32.217 | 0.000 | 0.000 |  |
| clusters.same.Mod + same.between | 935.058 | 3.000 | 703.000 | -1864.081 | 35.438 | 0.000 | 0.000 |  |
| clusters.sep.Mod + same.between | 948.288 | 8.000 | 703.000 | -1880.368 | 19.151 | 0.000 | 0.000 |  |
| clusters.same.Mod + sep.between | 959.273 | 17.000 | 703.000 | -1883.653 | 15.865 | 0.000 | 0.000 |  |
| clusters.sep.Mod + sep.between | 972.504 | 22.000 | 703.000 | -1899.519 | 0.000 | 1.000 | 1.000 |  |
|  |  |  |  |  |  |  |  |  |
| clusters.sep.Mod + sep.between |  |  |  |  |  |  |  |  |
|  | Module 1 | Module 2 | Module 3 | Module 6 | Module 4 | Module 5 | 1 to 2 | 1 to 3 |
| MaxL | 32.741 | 3.709 | 37.497 | 59.835 | 4.602 | -0.834 | 37.792 | 95.076 |
| MaxL_p | 0.240 | 0.160 | 0.150 | 0.170 | 0.300 | 0.420 | 0.100 | 0.140 |
|  | 1 to 6 | 1 to 4 | 1 to 5 | 2 to 3 | 2 to 6 | 2 to 4 | 2 to 5 | 3 to 6 |
|  | 119.662 | 31.686 | 50.442 | 36.833 | 45.752 | 13.234 | 28.708 | 124.492 |
|  | 0.120 | 0.230 | 0.250 | 0.140 | 0.100 | 0.150 | 0.060 | 0.100 |
|  | 3 to 4 | 3 to 5 | 6 to 4 | 6 to 5 | 4 to 5 | unintegrated |  |  |
|  | 35.022 | 73.344 | 42.140 | 72.925 | 27.846 | 0.000 |  |  |
|  | 0.140 | 0.110 | 0.150 | 0.200 | 0.270 | 0.000 |  |  |
|  |  |  |  |  |  |  |  |  |
| **After corrections** |  |  |  |  |  |  |  |  |
|  | MaxL | K | n | AICc | dAICc | Model_L | Post_Pob |  |
| No.modules.default | 927.738 | 2.000 | 703.000 | -1851.459 | 48.060 | 0.000 | 0.000 |  |
| Function.same.Mod + same.between | 932.373 | 3.000 | 703.000 | -1858.713 | 40.806 | 0.000 | 0.000 |  |
| Function.sep.Mod + same.between | 933.851 | 6.000 | 703.000 | -1855.582 | 43.937 | 0.000 | 0.000 |  |
| Function.same.Mod + sep.between | 938.956 | 8.000 | 703.000 | -1861.705 | 37.814 | 0.000 | 0.000 |  |
| Function.sep.Mod + sep.between | 940.434 | 11.000 | 703.000 | -1858.486 | 41.033 | 0.000 | 0.000 |  |
| Tissue.origin.same.Mod + same.between | 928.961 | 3.000 | 703.000 | -1851.888 | 47.631 | 0.000 | 0.000 |  |
| Tissue.origin.sep.Mod + same.between | 930.394 | 4.000 | 703.000 | -1852.731 | 46.788 | 0.000 | 0.000 |  |
| ossification.same.Mod + same.between | 928.583 | 3.000 | 703.000 | -1851.132 | 48.387 | 0.000 | 0.000 |  |
| ossification.sep.Mod + same.between | 928.602 | 4.000 | 703.000 | -1849.146 | 50.373 | 0.000 | 0.000 |  |
| front.back.same.Mod + same.between | 933.263 | 3.000 | 703.000 | -1860.491 | 39.028 | 0.000 | 0.000 |  |
| front.back.sep.Mod + same.between | 937.680 | 4.000 | 703.000 | -1867.302 | 32.217 | 0.000 | 0.000 |  |
| clusters.same.Mod + same.between | 935.058 | 3.000 | 703.000 | -1864.081 | 35.438 | 0.000 | 0.000 |  |
| clusters.sep.Mod + same.between | 948.288 | 8.000 | 703.000 | -1880.368 | 19.151 | 0.000 | 0.000 |  |
| clusters.same.Mod + sep.between | 959.273 | 17.000 | 703.000 | -1883.653 | 15.865 | 0.000 | 0.000 |  |
| clusters.sep.Mod + sep.between | 972.504 | 22.000 | 703.000 | -1899.519 | 0.000 | 1.000 | 1.000 |  |
|  |  |  |  |  |  |  |  |  |
| clusters.sep.Mod + sep.between |  |  |  |  |  |  |  |  |
|  | Module 1 | Module 2 | Module 3 | Module 6 | Module 4 | Module 5 | 1 to 2 | 1 to 3 |
| MaxL | 32.741 | 3.709 | 37.497 | 59.835 | 4.602 | -0.834 | 37.792 | 95.076 |
| MaxL_p | 0.240 | 0.160 | 0.150 | 0.170 | 0.300 | 0.420 | 0.100 | 0.140 |
|  | 1 to 6 | 1 to 4 | 1 to 5 | 2 to 3 | 2 to 6 | 2 to 4 | 2 to 5 | 3 to 6 |
|  | 119.662 | 31.686 | 50.442 | 36.833 | 45.752 | 13.234 | 28.708 | 124.492 |
|  | 0.120 | 0.230 | 0.250 | 0.140 | 0.100 | 0.150 | 0.060 | 0.100 |
|  | 3 to 4 | 3 to 5 | 6 to 4 | 6 to 5 | 4 to 5 | unintegrated |  |  |
|  | 35.022 | 73.344 | 42.140 | 72.925 | 27.846 | 0.000 |  |  |
|  | 0.140 | 0.110 | 0.150 | 0.200 | 0.270 | 0.000 |  |  |
|  |  |  |  |  |  |  |  |  |
| *Callorhinus ursinus* |  |  |  |  |  |  |  |  |
| **Before corrections:** |  |  |  |  |  |  |  |  |
|  | MaxL | K | n | AICc | dAICc | Model_L | Post_Pob |  |
| No.modules.default | 853.852 | 2.000 | 703.000 | -1703.687 | 71.463 | 0.000 | 0.000 |  |
| Function.same.Mod + same.between | 867.361 | 3.000 | 703.000 | -1728.687 | 46.462 | 0.000 | 0.000 |  |
| Function.sep.Mod + same.between | 871.725 | 6.000 | 703.000 | -1731.330 | 43.819 | 0.000 | 0.000 |  |
| Function.same.Mod + sep.between | 869.361 | 8.000 | 703.000 | -1722.515 | 52.635 | 0.000 | 0.000 |  |
| Function.sep.Mod + sep.between | 873.726 | 11.000 | 703.000 | -1725.070 | 50.080 | 0.000 | 0.000 |  |
| Tissue.origin.same.Mod + same.between | 853.852 | 3.000 | 703.000 | -1701.670 | 73.480 | 0.000 | 0.000 |  |
| Tissue.origin.sep.Mod + same.between | 855.061 | 4.000 | 703.000 | -1702.064 | 73.086 | 0.000 | 0.000 |  |
| ossification.same.Mod + same.between | 854.169 | 3.000 | 703.000 | -1702.304 | 72.845 | 0.000 | 0.000 |  |
| ossification.sep.Mod + same.between | 854.245 | 4.000 | 703.000 | -1700.432 | 74.717 | 0.000 | 0.000 |  |
| front.back.same.Mod + same.between | 858.147 | 3.000 | 703.000 | -1710.260 | 64.889 | 0.000 | 0.000 |  |
| front.back.sep.Mod + same.between | 858.870 | 4.000 | 703.000 | -1709.683 | 65.467 | 0.000 | 0.000 |  |
| clusters.same.Mod + same.between | 863.479 | 3.000 | 703.000 | -1720.925 | 54.225 | 0.000 | 0.000 |  |
| clusters.sep.Mod + same.between | 873.701 | 8.000 | 703.000 | -1731.194 | 43.956 | 0.000 | 0.000 |  |
| clusters.same.Mod + sep.between | 900.098 | 17.000 | 703.000 | -1765.302 | 9.847 | 0.007 | 0.007 |  |
| clusters.sep.Mod + sep.between | 910.319 | 22.000 | 703.000 | -1775.150 | 0.000 | 1.000 | 0.993 |  |
|  |  |  |  |  |  |  |  |  |
| clusters.sep.Mod + sep.between |  |  |  |  |  |  |  |  |
|  | Module 1 | Module 2 | Module 3 | Module 6 | Module 4 | Module 5 | 1 to 2 | 1 to 3 |
| MaxL | 30.321 | 2.342 | 36.143 | 57.807 | -0.132 | 8.615 | 27.903 | 90.114 |
| MaxL_p | 0.350 | 0.230 | 0.210 | 0.220 | 0.430 | 0.410 | 0.300 | 0.160 |
|  | 1 to 6 | 1 to 4 | 1 to 5 | 2 to 3 | 2 to 6 | 2 to 4 | 2 to 5 | 3 to 6 |
|  | 102.570 | 34.864 | 56.472 | 34.936 | 38.461 | 13.225 | 23.206 | 113.168 |
|  | 0.230 | 0.100 | 0.310 | 0.110 | 0.190 | 0.120 | 0.190 | 0.130 |
|  | 3 to 4 | 3 to 5 | 6 to 4 | 6 to 5 | 4 to 5 | unintegrated |  |  |
|  | 30.322 | 71.737 | 42.320 | 69.007 | 26.918 | 0.000 |  |  |
|  | 0.250 | 0.100 | 0.130 | 0.290 | 0.150 | 0.000 |  |  |
|  |  |  |  |  |  |  |  |  |
| **After corrections:** |  |  |  |  |  |  |  |  |
|  | MaxL | K | n | AICc | dAICc | Model_L | Post_Pob |  |
| clusters.same.Mod + sep.between | 982.351 | 17.000 | 703.000 | -1929.809 | 23.073 | 0.000 | 0.000 |  |
| Function.sep.Mod + sep.between | 979.082 | 11.000 | 703.000 | -1935.782 | 17.099 | 0.000 | 0.000 |  |
| Tissue.origin.sep.Mod + same.between | 971.977 | 4.000 | 703.000 | -1935.896 | 16.986 | 0.000 | 0.000 |  |
| clusters.sep.Mod + sep.between | 990.957 | 22.000 | 703.000 | -1936.426 | 16.456 | 0.000 | 0.000 |  |
| Tissue.origin.same.Mod + same.between | 971.888 | 3.000 | 703.000 | -1937.741 | 15.140 | 0.001 | 0.000 |  |
| ossification.sep.Mod + same.between | 972.952 | 4.000 | 703.000 | -1937.847 | 15.034 | 0.001 | 0.000 |  |
| No.modules.default | 971.045 | 2.000 | 703.000 | -1938.072 | 14.810 | 0.001 | 0.000 |  |
| ossification.same.Mod + same.between | 972.908 | 3.000 | 703.000 | -1939.781 | 13.101 | 0.001 | 0.001 |  |
| Function.same.Mod + sep.between | 978.533 | 8.000 | 703.000 | -1940.859 | 12.023 | 0.002 | 0.002 |  |
| front.back.sep.Mod + same.between | 975.547 | 4.000 | 703.000 | -1943.037 | 9.845 | 0.007 | 0.005 |  |
| front.back.same.Mod + same.between | 975.351 | 3.000 | 703.000 | -1944.667 | 8.215 | 0.016 | 0.012 |  |
| Function.sep.Mod + same.between | 978.600 | 6.000 | 703.000 | -1945.080 | 7.802 | 0.020 | 0.015 |  |
| clusters.same.Mod + same.between | 975.939 | 3.000 | 703.000 | -1945.844 | 7.038 | 0.030 | 0.022 |  |
| Function.same.Mod + same.between | 978.051 | 3.000 | 703.000 | -1950.069 | 2.813 | 0.245 | 0.185 |  |
| clusters.sep.Mod + same.between | 984.545 | 8.000 | 703.000 | -1952.882 | 0.000 | 1.000 | 0.755 |  |
|  |  |  |  |  |  |  |  |  |
| Function.same.Mod + same.between |  |  |  |  |  |  |  |  |
|  | withinModules | betweenModules | unintegrated |  |  |  |  |  |
| MaxL | 269.426 | 708.625 | 0.000 |  |  |  |  |  |
| MaxL_p | 0.190 | 0.130 | 0.000 |  |  |  |  |  |
|  |  |  |  |  |  |  |  |  |
| clusters.sep.Mod + same.between |  |  |  |  |  |  |  |  |
|  | Module 1 | Module 2 | Module 3 | Module 6 | Module 4 | Module 5 | betweenModules | unintegrated |
| MaxL | 35.626 | 3.344 | 39.088 | 63.190 | 2.402 | 12.656 | 828.239 | 0.000 |
| MaxL_p | 0.280 | 0.230 | 0.150 | 0.130 | 0.220 | 0.330 | 0.140 | 0.000 |
|  |  |  |  |  |  |  |  |  |
| *Otaria byronia* |  |  |  |  |  |  |  |  |
| **Before corrections:** |  |  |  |  |  |  |  |  |
|  | MaxL | K | n | AICc | dAICc | Model_L | Post_Pob |  |
| Function.sep.Mod + same.between | 1074.412 | 6.000 | 703.000 | -2136.702 | 32.462 | 0.000 | 0.000 |  |
| No.modules.default | 1071.936 | 2.000 | 703.000 | -2139.856 | 29.309 | 0.000 | 0.000 |  |
| Tissue.origin.same.Mod + same.between | 1073.166 | 3.000 | 703.000 | -2140.299 | 28.866 | 0.000 | 0.000 |  |
| Function.same.Mod + same.between | 1073.273 | 3.000 | 703.000 | -2140.511 | 28.654 | 0.000 | 0.000 |  |
| ossification.same.Mod + same.between | 1073.393 | 3.000 | 703.000 | -2140.753 | 28.412 | 0.000 | 0.000 |  |
| ossification.sep.Mod + same.between | 1074.808 | 4.000 | 703.000 | -2141.558 | 27.607 | 0.000 | 0.000 |  |
| Tissue.origin.sep.Mod + same.between | 1075.559 | 4.000 | 703.000 | -2143.061 | 26.104 | 0.000 | 0.000 |  |
| Function.sep.Mod + sep.between | 1083.896 | 11.000 | 703.000 | -2145.411 | 23.754 | 0.000 | 0.000 |  |
| Function.same.Mod + sep.between | 1082.757 | 8.000 | 703.000 | -2149.307 | 19.857 | 0.000 | 0.000 |  |
| front.back.sep.Mod + same.between | 1079.057 | 4.000 | 703.000 | -2150.057 | 19.108 | 0.000 | 0.000 |  |
| front.back.same.Mod + same.between | 1079.057 | 3.000 | 703.000 | -2152.080 | 17.085 | 0.000 | 0.000 |  |
| clusters.same.Mod + same.between | 1082.549 | 3.000 | 703.000 | -2159.064 | 10.101 | 0.006 | 0.005 |  |
| clusters.sep.Mod + same.between | 1090.262 | 8.000 | 703.000 | -2164.316 | 4.849 | 0.089 | 0.075 |  |
| clusters.same.Mod + sep.between | 1099.614 | 17.000 | 703.000 | -2164.335 | 4.830 | 0.089 | 0.075 |  |
| clusters.sep.Mod + sep.between | 1107.327 | 22.000 | 703.000 | -2169.165 | 0.000 | 1.000 | 0.844 |  |
|  |  |  |  |  |  |  |  |  |
|  |  |  |  |  |  |  |  |  |
| clusters.sep.Mod + sep.between |  |  |  |  |  |  |  |  |
|  | Module 1 | Module 2 | Module 3 | Module 6 | Module 4 | Module 5 | 1 to 2 | 1 to 3 |
| MaxL | 35.898 | 5.084 | 39.995 | 68.431 | 2.955 | 9.832 | 35.833 | 104.977 |
| MaxL_p | 0.260 | 0.150 | 0.190 | 0.170 | 0.400 | 0.270 | 0.150 | 0.140 |
|  | 1 to 6 | 1 to 4 | 1 to 5 | 2 to 3 | 2 to 6 | 2 to 4 | 2 to 5 | 3 to 6 |
|  | 128.551 | 40.223 | 68.266 | 40.328 | 51.827 | 15.292 | 30.276 | 137.548 |
|  | 0.140 | 0.120 | 0.230 | 0.190 | 0.130 | 0.170 | 0.100 | 0.110 |
|  | 3 to 4 | 3 to 5 | 6 to 4 | 6 to 5 | 4 to 5 | unintegrated |  |  |
|  | 42.909 | 83.004 | 41.791 | 95.146 | 29.164 | 0.000 |  |  |
|  | 0.130 | 0.110 | 0.180 | 0.160 | 0.190 | 0.000 |  |  |
|  |  |  |  |  |  |  |  |  |
| clusters.sep.Mod + same.between |  |  |  |  |  |  |  |  |
|  | Module 1 | Module 2 | Module 3 | Module 6 | Module 4 | Module 5 | betweenModules | unintegrated |
| MaxL | 35.898 | 5.084 | 39.995 | 68.431 | 2.955 | 9.832 | 928.067 | 0.000 |
| MaxL_p | 0.260 | 0.150 | 0.190 | 0.170 | 0.400 | 0.270 | 0.150 | 0.000 |
|  |  |  |  |  |  |  |  |  |
| clusters.same.Mod + sep.between |  |  |  |  |  |  |  |  |
|  | 1 to 2 | 1 to 3 | 1 to 6 | 1 to 4 | 1 to 5 | 2 to 3 | 2 to 6 | 2 to 4 |
| MaxL | 35.833 | 104.977 | 128.551 | 40.223 | 68.266 | 40.328 | 51.827 | 15.292 |
| MaxL_p | 0.150 | 0.140 | 0.140 | 0.120 | 0.230 | 0.190 | 0.130 | 0.170 |
|  | 2 to 5 | 3 to 6 | 3 to 4 | 3 to 5 | 6 to 4 | 6 to 5 | 4 to 5 | withinModules |
|  | 30.276 | 137.548 | 42.909 | 83.004 | 41.791 | 95.146 | 29.164 | 154.482 |
|  | 0.100 | 0.110 | 0.130 | 0.110 | 0.180 | 0.160 | 0.190 | 0.210 |
|  | unintegrated |  |  |  |  |  |  |  |
|  | 0.000 |  |  |  |  |  |  |  |
|  | 0.000 |  |  |  |  |  |  |  |
|  |  |  |  |  |  |  |  |  |
| **After corrections:** |  |  |  |  |  |  |  |  |
| Females: |  |  |  |  |  |  |  |  |
|  | MaxL | K | n | AICc | dAICc | Model_L | Post_Pob |  |
| clusters.sep.Mod + sep.between | 1055.149 | 22.000 | 703.000 | -2064.809 | 30.323 | 0.000 | 0.000 |  |
| clusters.same.Mod + sep.between | 1053.722 | 17.000 | 703.000 | -2072.550 | 22.583 | 0.000 | 0.000 |  |
| Function.sep.Mod + sep.between | 1051.861 | 11.000 | 703.000 | -2081.340 | 13.792 | 0.001 | 0.000 |  |
| ossification.same.Mod + same.between | 1045.885 | 3.000 | 703.000 | -2085.736 | 9.396 | 0.009 | 0.003 |  |
| Tissue.origin.same.Mod + same.between | 1046.063 | 3.000 | 703.000 | -2086.092 | 9.040 | 0.011 | 0.004 |  |
| No.modules.default | 1045.301 | 2.000 | 703.000 | -2086.585 | 8.547 | 0.014 | 0.005 |  |
| Function.same.Mod + sep.between | 1051.434 | 8.000 | 703.000 | -2086.661 | 8.471 | 0.014 | 0.005 |  |
| clusters.sep.Mod + same.between | 1052.010 | 8.000 | 703.000 | -2087.813 | 7.319 | 0.026 | 0.009 |  |
| Function.sep.Mod + same.between | 1050.119 | 6.000 | 703.000 | -2088.117 | 7.015 | 0.030 | 0.011 |  |
| Tissue.origin.sep.Mod + same.between | 1049.488 | 4.000 | 703.000 | -2090.918 | 4.214 | 0.122 | 0.044 |  |
| front.back.sep.Mod + same.between | 1050.260 | 4.000 | 703.000 | -2092.462 | 2.671 | 0.263 | 0.096 |  |
| ossification.sep.Mod + same.between | 1050.276 | 4.000 | 703.000 | -2092.495 | 2.638 | 0.267 | 0.098 |  |
| Function.same.Mod + same.between | 1049.692 | 3.000 | 703.000 | -2093.349 | 1.783 | 0.410 | 0.150 |  |
| front.back.same.Mod + same.between | 1050.021 | 3.000 | 703.000 | -2094.008 | 1.125 | 0.570 | 0.208 |  |
| clusters.same.Mod + same.between | 1050.583 | 3.000 | 703.000 | -2095.132 | 0.000 | 1.000 | 0.365 |  |
|  |  |  |  |  |  |  |  |  |
| Function.same.Mod + same.between |  |  |  |  |  |  |  |  |
|  | withinModules | betweenModules | unintegrated |  |  |  |  |  |
| MaxL | 295.411 | 754.280 | 0.000 |  |  |  |  |  |
| MaxL_p | 0.170 | 0.120 | 0.000 |  |  |  |  |  |
|  |  |  |  |  |  |  |  |  |
| ossification.sep.Mod + same.between |  |  |  |  |  |  |  |  |
|  | Module 1 | Module 2 | betweenModules | unintegrated |  |  |  |  |
| MaxL | 604.661 | 43.414 | 402.201 | 0.000 |  |  |  |  |
| MaxL_p | 0.130 | 0.230 | 0.130 | 0.000 |  |  |  |  |
|  |  |  |  |  |  |  |  |  |
| front.back.sep.Mod + same.between |  |  |  |  |  |  |  |  |
|  | Module 1 | Module 2 | betweenModules | unintegrated |  |  |  |  |
| MaxL | 250.355 | 236.444 | 563.461 | 0.000 |  |  |  |  |
| MaxL_p | 0.150 | 0.160 | 0.110 | 0.000 |  |  |  |  |
|  |  |  |  |  |  |  |  |  |
| front.back.same.Mod + same.between |  |  |  |  |  |  |  |  |
|  | withinModules | betweenModules | unintegrated |  |  |  |  |  |
| MaxL | 486.560 | 563.461 | 0.000 |  |  |  |  |  |
| MaxL_p | 0.160 | 0.110 | 0.000 |  |  |  |  |  |
|  |  |  |  |  |  |  |  |  |
| clusters.same.Mod + same.between |  |  |  |  |  |  |  |  |
|  | withinModules | betweenModules | unintegrated |  |  |  |  |  |
| MaxL | 159.053 | 891.531 | 0.000 |  |  |  |  |  |
| MaxL_p | 0.180 | 0.120 | 0.000 |  |  |  |  |  |
|  |  |  |  |  |  |  |  |  |
| Males: |  |  |  |  |  |  |  |  |
|  | MaxL | K | n | AICc | dAICc | Model_L | Post_Pob |  |
| clusters.sep.Mod + sep.between | 874.527 | 22.000 | 703.000 | -1703.566 | 30.424 | 0.000 | 0.000 |  |
| clusters.same.Mod + sep.between | 872.688 | 17.000 | 703.000 | -1710.483 | 23.507 | 0.000 | 0.000 |  |
| Function.sep.Mod + sep.between | 870.998 | 11.000 | 703.000 | -1719.615 | 14.375 | 0.001 | 0.000 |  |
| Function.sep.Mod + same.between | 867.758 | 6.000 | 703.000 | -1723.396 | 10.594 | 0.005 | 0.002 |  |
| Function.same.Mod + sep.between | 870.179 | 8.000 | 703.000 | -1724.150 | 9.840 | 0.007 | 0.004 |  |
| ossification.same.Mod + same.between | 865.135 | 3.000 | 703.000 | -1724.236 | 9.754 | 0.008 | 0.004 |  |
| Tissue.origin.same.Mod + same.between | 865.413 | 3.000 | 703.000 | -1724.792 | 9.198 | 0.010 | 0.005 |  |
| No.modules.default | 864.729 | 2.000 | 703.000 | -1725.441 | 8.549 | 0.014 | 0.007 |  |
| Tissue.origin.sep.Mod + same.between | 867.501 | 4.000 | 703.000 | -1726.944 | 7.046 | 0.030 | 0.015 |  |
| ossification.sep.Mod + same.between | 867.602 | 4.000 | 703.000 | -1727.147 | 6.843 | 0.033 | 0.016 |  |
| clusters.sep.Mod + same.between | 871.851 | 8.000 | 703.000 | -1727.495 | 6.495 | 0.039 | 0.019 |  |
| Function.same.Mod + same.between | 866.939 | 3.000 | 703.000 | -1727.843 | 6.147 | 0.046 | 0.023 |  |
| front.back.same.Mod + same.between | 869.009 | 3.000 | 703.000 | -1731.983 | 2.007 | 0.367 | 0.182 |  |
| front.back.sep.Mod + same.between | 870.236 | 4.000 | 703.000 | -1732.415 | 1.575 | 0.455 | 0.226 |  |
| clusters.same.Mod + same.between | 870.012 | 3.000 | 703.000 | -1733.990 | 0.000 | 1.000 | 0.497 |  |
|  |  |  |  |  |  |  |  |  |
| front.back.sep.Mod + same.between |  |  |  |  |  |  |  |  |
|  | Module 1 | Module 2 | betweenModules | unintegrated |  |  |  |  |
| MaxL | 208.570 | 199.116 | 462.551 | 0.000 |  |  |  |  |
| MaxL_p | 0.170 | 0.210 | 0.130 | 0.000 |  |  |  |  |
|  |  |  |  |  |  |  |  |  |
| front.back.same.Mod + same.between |  |  |  |  |  |  |  |  |
|  | withinModules | betweenModules | unintegrated |  |  |  |  |  |
| MaxL | 406.458 | 462.551 | 0.000 |  |  |  |  |  |
| MaxL_p | 0.190 | 0.130 | 0.000 |  |  |  |  |  |
|  |  |  |  |  |  |  |  |  |
| clusters.same.Mod + same.between |  |  |  |  |  |  |  |  |
|  | withinModules | betweenModules | unintegrated |  |  |  |  |  |
| MaxL | 135.656 | 734.356 | 0.000 |  |  |  |  |  |
| MaxL_p | 0.230 | 0.150 | 0.000 |  |  |  |  |  |
|  |  |  |  |  |  |  |  |  |
| *Zalophus californianus* |  |  |  |  |  |  |  |  |
| **Before corrections:** |  |  |  |  |  |  |  |  |
|  | MaxL | K | n | AICc | dAICc | Model_L | Post_Pob |  |
| Function.sep.Mod + same.between | 1059.262 | 6.000 | 703.000 | -2106.404 | 23.685 | 0.000 | 0.000 |  |
| No.modules.default | 1057.117 | 2.000 | 703.000 | -2110.217 | 19.872 | 0.000 | 0.000 |  |
| Tissue.origin.same.Mod + same.between | 1058.183 | 3.000 | 703.000 | -2110.332 | 19.757 | 0.000 | 0.000 |  |
| Function.same.Mod + same.between | 1058.275 | 3.000 | 703.000 | -2110.516 | 19.573 | 0.000 | 0.000 |  |
| ossification.same.Mod + same.between | 1058.380 | 3.000 | 703.000 | -2110.726 | 19.363 | 0.000 | 0.000 |  |
| ossification.sep.Mod + same.between | 1059.606 | 4.000 | 703.000 | -2111.154 | 18.935 | 0.000 | 0.000 |  |
| Tissue.origin.sep.Mod + same.between | 1060.257 | 4.000 | 703.000 | -2112.457 | 17.632 | 0.000 | 0.000 |  |
| Function.sep.Mod + sep.between | 1067.483 | 11.000 | 703.000 | -2112.583 | 17.506 | 0.000 | 0.000 |  |
| Function.same.Mod + sep.between | 1066.495 | 8.000 | 703.000 | -2116.783 | 13.306 | 0.001 | 0.001 |  |
| front.back.sep.Mod + same.between | 1063.288 | 4.000 | 703.000 | -2118.520 | 11.569 | 0.003 | 0.001 |  |
| front.back.same.Mod + same.between | 1063.288 | 3.000 | 703.000 | -2120.542 | 9.547 | 0.008 | 0.004 |  |
| clusters.same.Mod + same.between | 1066.315 | 3.000 | 703.000 | -2126.595 | 3.494 | 0.174 | 0.076 |  |
| clusters.same.Mod + sep.between | 1081.105 | 17.000 | 703.000 | -2127.316 | 2.773 | 0.250 | 0.109 |  |
| clusters.sep.Mod + same.between | 1072.999 | 8.000 | 703.000 | -2129.790 | 0.299 | 0.861 | 0.375 |  |
| clusters.sep.Mod + sep.between | 1087.789 | 22.000 | 703.000 | -2130.089 | 0.000 | 1.000 | 0.435 |  |
|  |  |  |  |  |  |  |  |  |
| clusters.sep.Mod + sep.between |  |  |  |  |  |  |  |  |
|  | Module 1 | Module 2 | Module 3 | Module 6 | Module 4 | Module 5 | 1 to 2 | 1 to 3 |
| MaxL | 36.214 | 4.952 | 39.764 | 67.507 | 3.108 | 11.255 | 35.428 | 102.643 |
| MaxL_p | 0.260 | 0.150 | 0.190 | 0.170 | 0.400 | 0.270 | 0.150 | 0.140 |
|  | 1 to 6 | 1 to 4 | 1 to 5 | 2 to 3 | 2 to 6 | 2 to 4 | 2 to 5 | 3 to 6 |
|  | 125.989 | 39.233 | 67.911 | 39.324 | 50.383 | 14.894 | 29.519 | 133.786 |
|  | 0.140 | 0.120 | 0.230 | 0.190 | 0.130 | 0.170 | 0.100 | 0.110 |
|  | 3 to 4 | 3 to 5 | 6 to 4 | 6 to 5 | 4 to 5 | unintegrated |  |  |
|  | 41.561 | 80.683 | 41.685 | 93.393 | 28.555 | 0.000 |  |  |
|  | 0.130 | 0.110 | 0.180 | 0.160 | 0.190 | 0.000 |  |  |
|  |  |  |  |  |  |  |  |  |
| clusters.same.Mod + same.between |  |  |  |  |  |  |  |  |
|  | withinModules | betweenModules | unintegrated |  |  |  |  |  |
| MaxL | 156.116 | 910.199 | 0 |  |  |  |  |  |
| MaxL_p | 0.210 | 0.150 | 0 |  |  |  |  |  |
|  |  |  |  |  |  |  |  |  |
| clusters.sep.Mod + same.between |  |  |  |  |  |  |  |  |
|  | Module 1 | Module 2 | Module 3 | Module 6 | Module 4 | Module 5 | betweenModules | unintegrated |
| MaxL | 36.214 | 4.952 | 39.764 | 67.507 | 3.108 | 11.255 | 910.199 | 0.000 |
| MaxL_p | 0.260 | 0.150 | 0.190 | 0.170 | 0.400 | 0.270 | 0.150 | 0.000 |
|  |  |  |  |  |  |  |  |  |
| clusters.same.Mod + sep.between |  |  |  |  |  |  |  |  |
|  | 1 to 2 | 1 to 3 | 1 to 6 | 1 to 4 | 1 to 5 | 2 to 3 | 2 to 6 | 2 to 4 |
| MaxL | 35.428 | 102.643 | 125.989 | 39.233 | 67.911 | 39.324 | 50.383 | 14.894 |
| MaxL_p | 0.150 | 0.140 | 0.140 | 0.120 | 0.230 | 0.190 | 0.130 | 0.170 |
|  | 2 to 5 | 3 to 6 | 3 to 4 | 3 to 5 | 6 to 4 | 6 to 5 | 4 to 5 | withinModules |
|  | 29.519 | 133.786 | 41.561 | 80.683 | 41.685 | 93.393 | 28.555 | 156.116 |
|  | 0.100 | 0.110 | 0.130 | 0.110 | 0.180 | 0.160 | 0.190 | 0.210 |
|  | unintegrated |  |  |  |  |  |  |  |
|  | 0.000 |  |  |  |  |  |  |  |
|  | 0.000 |  |  |  |  |  |  |  |
|  |  |  |  |  |  |  |  |  |
| **After corrections:** |  |  |  |  |  |  |  |  |
|  | MaxL | K | n | AICc | dAICc | Model_L | Post_Pob |  |
| No.modules.default | 1106.559 | 2.000 | 703.000 | -2209.101 | 25.399 | 0.000 | 0.000 |  |
| Function.same.Mod + same.between | 1116.958 | 3.000 | 703.000 | -2227.881 | 6.619 | 0.037 | 0.021 |  |
| Function.sep.Mod + same.between | 1119.531 | 6.000 | 703.000 | -2226.941 | 7.559 | 0.023 | 0.013 |  |
| Function.same.Mod + sep.between | 1121.885 | 8.000 | 703.000 | -2227.563 | 6.937 | 0.031 | 0.018 |  |
| Function.sep.Mod + sep.between | 1124.458 | 11.000 | 703.000 | -2226.534 | 7.966 | 0.019 | 0.011 |  |
| Tissue.origin.same.Mod + same.between | 1110.093 | 3.000 | 703.000 | -2214.151 | 20.349 | 0.000 | 0.000 |  |
| Tissue.origin.sep.Mod + same.between | 1114.455 | 4.000 | 703.000 | -2220.853 | 13.647 | 0.001 | 0.001 |  |
| ossification.same.Mod + same.between | 1107.649 | 3.000 | 703.000 | -2209.263 | 25.237 | 0.000 | 0.000 |  |
| ossification.sep.Mod + same.between | 1111.465 | 4.000 | 703.000 | -2214.873 | 19.627 | 0.000 | 0.000 |  |
| front.back.same.Mod + same.between | 1120.267 | 3.000 | 703.000 | -2234.500 | 0.000 | 1.000 | 0.577 |  |
| front.back.sep.Mod + same.between | 1120.547 | 4.000 | 703.000 | -2233.037 | 1.463 | 0.481 | 0.278 |  |
| clusters.same.Mod + same.between | 1118.118 | 3.000 | 703.000 | -2230.201 | 4.299 | 0.117 | 0.067 |  |
| clusters.sep.Mod + same.between | 1119.424 | 8.000 | 703.000 | -2222.640 | 11.860 | 0.003 | 0.002 |  |
| clusters.same.Mod + sep.between | 1130.812 | 17.000 | 703.000 | -2226.731 | 7.769 | 0.021 | 0.012 |  |
| clusters.sep.Mod + sep.between | 1132.119 | 22.000 | 703.000 | -2218.749 | 15.751 | 0.000 | 0.000 |  |
|  |  |  |  |  |  |  |  |  |
|  |  |  |  |  |  |  |  |  |
| front.back.sep.Mod + same.between |  |  |  |  |  |  |  |  |
|  | Module 1 | Module 2 | betweenModules | unintegrated |  |  |  |  |
| MaxL | 248.455 | 247.362 | 624.730 | 0.000 |  |  |  |  |
| MaxL_p | 0.170 | 0.150 | 0.100 | 0.000 |  |  |  |  |
|  |  |  |  |  |  |  |  |  |
| front.back.same.Mod + same.between |  |  |  |  |  |  |  |  |
|  | withinModules | betweenModules | unintegrated |  |  |  |  |  |
| MaxL | 495.537 | 624.730 | 0.000 |  |  |  |  |  |
| MaxL_p | 0.160 | 0.100 | 0.000 |  |  |  |  |  |
|  |  |  |  |  |  |  |  |  |
| clusters.same.Mod + same.between |  |  |  |  |  |  |  |  |
|  | withinModules | betweenModules | unintegrated |  |  |  |  |  |
| MaxL | 155.759 | 962.359 | 0.000 |  |  |  |  |  |
| MaxL_p | 0.190 | 0.120 | 0.000 |  |  |  |  |  |
|  |  |  |  |  |  |  |  |  |
| **Phocidae** |  |  |  |  |  |  |  |  |
| *Cystophora cristata* |  |  |  |  |  |  |  |  |
| **Before corrections:** |  |  |  |  |  |  |  |  |
|  | MaxL | K | n | AICc | dAICc | Model_L | Post_Pob |  |
| Tissue.origin.sep.Mod + same.between | 1070.463 | 4.000 | 703.000 | -2132.868 | 40.226 | 0.000 | 0.000 |  |
| ossification.same.Mod + same.between | 1070.082 | 3.000 | 703.000 | -2134.130 | 38.964 | 0.000 | 0.000 |  |
| Tissue.origin.same.Mod + same.between | 1070.334 | 3.000 | 703.000 | -2134.634 | 38.460 | 0.000 | 0.000 |  |
| No.modules.default | 1070.082 | 2.000 | 703.000 | -2136.147 | 36.947 | 0.000 | 0.000 |  |
| ossification.sep.Mod + same.between | 1072.177 | 4.000 | 703.000 | -2136.297 | 36.797 | 0.000 | 0.000 |  |
| clusters.sep.Mod + same.between | 1079.944 | 8.000 | 703.000 | -2143.680 | 29.414 | 0.000 | 0.000 |  |
| clusters.same.Mod + same.between | 1076.974 | 3.000 | 703.000 | -2147.913 | 25.181 | 0.000 | 0.000 |  |
| Function.sep.Mod + same.between | 1082.447 | 6.000 | 703.000 | -2152.774 | 20.319 | 0.000 | 0.000 |  |
| Function.same.Mod + same.between | 1082.316 | 3.000 | 703.000 | -2158.597 | 14.497 | 0.001 | 0.000 |  |
| Function.sep.Mod + sep.between | 1091.604 | 11.000 | 703.000 | -2160.825 | 12.269 | 0.002 | 0.001 |  |
| Function.same.Mod + sep.between | 1091.472 | 8.000 | 703.000 | -2166.736 | 6.358 | 0.042 | 0.015 |  |
| clusters.sep.Mod + sep.between | 1106.793 | 22.000 | 703.000 | -2168.097 | 4.996 | 0.082 | 0.029 |  |
| front.back.sep.Mod + same.between | 1090.400 | 4.000 | 703.000 | -2172.743 | 0.351 | 0.839 | 0.299 |  |
| clusters.same.Mod + sep.between | 1103.823 | 17.000 | 703.000 | -2172.752 | 0.341 | 0.843 | 0.300 |  |
| front.back.same.Mod + same.between | 1089.564 | 3.000 | 703.000 | -2173.094 | 0.000 | 1.000 | 0.356 |  |
|  |  |  |  |  |  |  |  |  |
| front.back.sep.Mod + same.between |  |  |  |  |  |  |  |  |
|  | Module 1 | Module 2 | betweenModules | unintegrated |  |  |  |  |
| MaxL | 239.927 | 236.631 | 613.843 | 0.000 |  |  |  |  |
| MaxL_p | 0.200 | 0.190 | 0.130 | 0.000 |  |  |  |  |
|  |  |  |  |  |  |  |  |  |
| front.back.same.Mod + same.between |  |  |  |  |  |  |  |  |
|  | withinModules | betweenModules | unintegrated |  |  |  |  |  |
| MaxL | 475.721 | 613.843 | 0.000 |  |  |  |  |  |
| MaxL_p | 0.200 | 0.130 | 0.000 |  |  |  |  |  |
|  |  |  |  |  |  |  |  |  |
| clusters.same.Mod + sep.between |  |  |  |  |  |  |  |  |
|  | 1 to 2 | 1 to 3 | 1 to 6 | 1 to 4 | 1 to 5 | 2 to 3 | 2 to 6 | 2 to 4 |
| MaxL | 28.919 | 98.684 | 133.861 | 39.619 | 81.036 | 36.691 | 48.048 | 16.455 |
| MaxL_p | 0.280 | 0.200 | 0.160 | 0.130 | 0.150 | 0.170 | 0.150 | 0.130 |
|  | 2 to 5 | 3 to 6 | 3 to 4 | 3 to 5 | 6 to 4 | 6 to 5 | 4 to 5 | withinModules |
|  | 31.419 | 141.524 | 39.888 | 89.043 | 47.735 | 93.640 | 32.404 | 144.858 |
|  | 0.110 | 0.100 | 0.110 | 0.080 | 0.160 | 0.180 | 0.150 | 0.200 |
|  | unintegrated |  |  |  |  |  |  |  |
|  | 0.000 |  |  |  |  |  |  |  |
|  | 0.000 |  |  |  |  |  |  |  |
|  |  |  |  |  |  |  |  |  |
| **After corrections:** |  |  |  |  |  |  |  |  |
|  | MaxL | K | n | AICc | dAICc | Model_L | Post_Pob |  |
| Tissue.origin.sep.Mod + same.between | 1176.336 | 4.000 | 703.000 | -2344.615 | 22.664 | 0.000 | 0.000 |  |
| ossification.same.Mod + same.between | 1175.857 | 3.000 | 703.000 | -2345.679 | 21.599 | 0.000 | 0.000 |  |
| Tissue.origin.same.Mod + same.between | 1175.871 | 3.000 | 703.000 | -2345.707 | 21.571 | 0.000 | 0.000 |  |
| ossification.sep.Mod + same.between | 1177.425 | 4.000 | 703.000 | -2346.793 | 20.485 | 0.000 | 0.000 |  |
| clusters.sep.Mod + sep.between | 1196.392 | 22.000 | 703.000 | -2347.295 | 19.983 | 0.000 | 0.000 |  |
| No.modules.default | 1175.670 | 2.000 | 703.000 | -2347.324 | 19.955 | 0.000 | 0.000 |  |
| Function.sep.Mod + same.between | 1180.692 | 6.000 | 703.000 | -2349.264 | 18.014 | 0.000 | 0.000 |  |
| clusters.sep.Mod + same.between | 1183.955 | 8.000 | 703.000 | -2351.703 | 15.575 | 0.000 | 0.000 |  |
| clusters.same.Mod + sep.between | 1193.401 | 17.000 | 703.000 | -2351.908 | 15.370 | 0.000 | 0.000 |  |
| Function.same.Mod + same.between | 1180.150 | 3.000 | 703.000 | -2354.266 | 13.012 | 0.001 | 0.001 |  |
| Function.sep.Mod + sep.between | 1189.137 | 11.000 | 703.000 | -2355.892 | 11.386 | 0.003 | 0.002 |  |
| clusters.same.Mod + same.between | 1180.964 | 3.000 | 703.000 | -2355.894 | 11.384 | 0.003 | 0.002 |  |
| Function.same.Mod + sep.between | 1188.595 | 8.000 | 703.000 | -2360.983 | 6.296 | 0.043 | 0.027 |  |
| front.back.sep.Mod + same.between | 1187.091 | 4.000 | 703.000 | -2366.125 | 1.153 | 0.562 | 0.348 |  |
| front.back.same.Mod + same.between | 1186.656 | 3.000 | 703.000 | -2367.278 | 0.000 | 1.000 | 0.619 |  |
|  |  |  |  |  |  |  |  |  |
| front.back.sep.Mod + same.between |  |  |  |  |  |  |  |  |
|  | Module 1 | Module 2 | betweenModules | unintegrated |  |  |  |  |
| MaxL | 285.681 | 258.847 | 642.564 | 0.000 |  |  |  |  |
| MaxL_p | 0.150 | 0.160 | 0.100 | 0.000 |  |  |  |  |
|  |  |  |  |  |  |  |  |  |
| front.back.same.Mod + same.between |  |  |  |  |  |  |  |  |
|  | withinModules | betweenModules | unintegrated |  |  |  |  |  |
| MaxL | 544.093 | 642.564 | 0.000 |  |  |  |  |  |
| MaxL_p | 0.150 | 0.100 | 0.000 |  |  |  |  |  |
|  |  |  |  |  |  |  |  |  |
| *Halichoerus grypus* |  |  |  |  |  |  |  |  |
| **Before corrections:** |  |  |  |  |  |  |  |  |
|  | MaxL | K | n | AICc | dAICc | Model_L | Post_Pob |  |
| No.modules.default | 935.443 | 2.000 | 703.000 | -1866.869 | 216.729 | 0.000 | 0.000 |  |
| Function.same.Mod + same.between | 976.630 | 3.000 | 703.000 | -1947.225 | 136.373 | 0.000 | 0.000 |  |
| Function.sep.Mod + same.between | 1023.389 | 6.000 | 703.000 | -2034.657 | 48.941 | 0.000 | 0.000 |  |
| Function.same.Mod + sep.between | 1001.954 | 8.000 | 703.000 | -1987.700 | 95.898 | 0.000 | 0.000 |  |
| Function.sep.Mod + sep.between | 1048.713 | 11.000 | 703.000 | -2075.044 | 8.554 | 0.014 | 0.014 |  |
| Tissue.origin.same.Mod + same.between | 935.443 | 3.000 | 703.000 | -1864.851 | 218.747 | 0.000 | 0.000 |  |
| Tissue.origin.sep.Mod + same.between | 963.299 | 4.000 | 703.000 | -1918.541 | 165.057 | 0.000 | 0.000 |  |
| ossification.same.Mod + same.between | 936.222 | 3.000 | 703.000 | -1866.410 | 217.188 | 0.000 | 0.000 |  |
| ossification.sep.Mod + same.between | 955.840 | 4.000 | 703.000 | -1903.623 | 179.975 | 0.000 | 0.000 |  |
| front.back.same.Mod + same.between | 982.029 | 3.000 | 703.000 | -1958.025 | 125.573 | 0.000 | 0.000 |  |
| front.back.sep.Mod + same.between | 1037.519 | 4.000 | 703.000 | -2066.980 | 16.618 | 0.000 | 0.000 |  |
| clusters.same.Mod + same.between | 963.312 | 3.000 | 703.000 | -1920.590 | 163.008 | 0.000 | 0.000 |  |
| clusters.sep.Mod + same.between | 994.405 | 8.000 | 703.000 | -1972.602 | 110.996 | 0.000 | 0.000 |  |
| clusters.same.Mod + sep.between | 1033.451 | 17.000 | 703.000 | -2032.008 | 51.590 | 0.000 | 0.000 |  |
| clusters.sep.Mod + sep.between | 1064.543 | 22.000 | 703.000 | -2083.598 | 0.000 | 1.000 | 0.986 |  |
|  |  |  |  |  |  |  |  |  |
|  |  |  |  |  |  |  |  |  |
| clusters.sep.Mod + sep.between |  |  |  |  |  |  |  |  |
|  | Module 1 | Module 2 | Module 3 | Module 6 | Module 4 | Module 5 | 1 to 2 | 1 to 3 |
| MaxL | 38.316 | 5.565 | 44.443 | 41.732 | 2.398 | -19.467 | 39.280 | 110.145 |
| MaxL_p | 0.210 | 0.240 | 0.090 | 0.320 | 0.420 | 0.350 | 0.200 | 0.100 |
|  | 1 to 6 | 1 to 4 | 1 to 5 | 2 to 3 | 2 to 6 | 2 to 4 | 2 to 5 | 3 to 6 |
|  | 140.772 | 37.497 | 79.094 | 37.096 | 54.210 | 13.567 | 32.523 | 142.298 |
|  | 0.110 | 0.130 | 0.160 | 0.120 | 0.120 | 0.160 | 0.090 | 0.110 |
|  | 3 to 4 | 3 to 5 | 6 to 4 | 6 to 5 | 4 to 5 | unintegrated |  |  |
|  | 40.297 | 87.866 | 43.150 | 65.754 | 28.007 | 0.000 |  |  |
|  | 0.110 | 0.080 | 0.220 | 0.310 | 0.220 | 0.000 |  |  |
|  |  |  |  |  |  |  |  |  |
| **After corrections:** |  |  |  |  |  |  |  |  |
| Females: |  |  |  |  |  |  |  |  |
|  | MaxL | K | n | AICc | dAICc | Model_L | Post_Pob |  |
| ossification.same.Mod + same.between | 924.929 | 3.000 | 703.000 | -1843.823 | 27.730 | 0.000 | 0.000 |  |
| Tissue.origin.same.Mod + same.between | 925.136 | 3.000 | 703.000 | -1844.237 | 27.316 | 0.000 | 0.000 |  |
| No.modules.default | 924.929 | 2.000 | 703.000 | -1845.840 | 25.713 | 0.000 | 0.000 |  |
| ossification.sep.Mod + same.between | 927.388 | 4.000 | 703.000 | -1846.719 | 24.834 | 0.000 | 0.000 |  |
| Tissue.origin.sep.Mod + same.between | 927.732 | 4.000 | 703.000 | -1847.406 | 24.147 | 0.000 | 0.000 |  |
| Function.same.Mod + same.between | 929.774 | 3.000 | 703.000 | -1853.514 | 18.039 | 0.000 | 0.000 |  |
| clusters.same.Mod + sep.between | 944.552 | 17.000 | 703.000 | -1854.211 | 17.343 | 0.000 | 0.000 |  |
| Function.sep.Mod + same.between | 933.629 | 6.000 | 703.000 | -1855.138 | 16.416 | 0.000 | 0.000 |  |
| clusters.same.Mod + same.between | 931.154 | 3.000 | 703.000 | -1856.274 | 15.280 | 0.000 | 0.000 |  |
| clusters.sep.Mod + sep.between | 954.167 | 22.000 | 703.000 | -1862.846 | 8.707 | 0.013 | 0.011 |  |
| Function.same.Mod + sep.between | 939.894 | 8.000 | 703.000 | -1863.580 | 7.974 | 0.019 | 0.015 |  |
| Function.sep.Mod + sep.between | 943.748 | 11.000 | 703.000 | -1865.115 | 6.439 | 0.040 | 0.033 |  |
| clusters.sep.Mod + same.between | 940.769 | 8.000 | 703.000 | -1865.331 | 6.222 | 0.045 | 0.037 |  |
| front.back.same.Mod + same.between | 936.522 | 3.000 | 703.000 | -1867.009 | 4.544 | 0.103 | 0.085 |  |
| front.back.sep.Mod + same.between | 939.805 | 4.000 | 703.000 | -1871.553 | 0.000 | 1.000 | 0.820 |  |
|  |  |  |  |  |  |  |  |  |
| front.back.sep.Mod + same.between |  |  |  |  |  |  |  |  |
|  | Module 1 | Module 2 | betweenModules | unintegrated |  |  |  |  |
| MaxL | 214.143 | 206.584 | 519.078 | 0.000 |  |  |  |  |
| MaxL_p | 0.180 | 0.230 | 0.130 | 0.000 |  |  |  |  |
|  |  |  |  |  |  |  |  |  |
| front.back.same.Mod + same.between |  |  |  |  |  |  |  |  |
|  | withinModules | betweenModules | unintegrated |  |  |  |  |  |
| MaxL | 417.444 | 519.078 | 0.000 |  |  |  |  |  |
| MaxL_p | 0.200 | 0.130 | 0.000 |  |  |  |  |  |
|  |  |  |  |  |  |  |  |  |
| Males: |  |  |  |  |  |  |  |  |
|  | MaxL | K | n | AICc | dAICc | Model_L | Post_Pob |  |
| clusters.same.Mod + sep.between | 890.153 | 17.000 | 703.000 | -1745.413 | 22.212 | 0.000 | 0.000 |  |
| ossification.sep.Mod + same.between | 878.280 | 4.000 | 703.000 | -1748.502 | 19.123 | 0.000 | 0.000 |  |
| No.modules.default | 877.034 | 2.000 | 703.000 | -1750.052 | 17.573 | 0.000 | 0.000 |  |
| Tissue.origin.same.Mod + same.between | 878.047 | 3.000 | 703.000 | -1750.060 | 17.564 | 0.000 | 0.000 |  |
| ossification.same.Mod + same.between | 878.106 | 3.000 | 703.000 | -1750.177 | 17.448 | 0.000 | 0.000 |  |
| Function.sep.Mod + same.between | 881.239 | 6.000 | 703.000 | -1750.357 | 17.268 | 0.000 | 0.000 |  |
| Tissue.origin.sep.Mod + same.between | 879.276 | 4.000 | 703.000 | -1750.495 | 17.130 | 0.000 | 0.000 |  |
| Function.same.Mod + same.between | 879.881 | 3.000 | 703.000 | -1753.728 | 13.897 | 0.001 | 0.001 |  |
| Function.sep.Mod + sep.between | 888.326 | 11.000 | 703.000 | -1754.271 | 13.354 | 0.001 | 0.001 |  |
| clusters.same.Mod + same.between | 880.587 | 3.000 | 703.000 | -1755.140 | 12.485 | 0.002 | 0.001 |  |
| clusters.sep.Mod + sep.between | 900.684 | 22.000 | 703.000 | -1755.881 | 11.744 | 0.003 | 0.002 |  |
| Function.same.Mod + sep.between | 886.969 | 8.000 | 703.000 | -1757.731 | 9.894 | 0.007 | 0.004 |  |
| front.back.same.Mod + same.between | 885.731 | 3.000 | 703.000 | -1765.427 | 2.198 | 0.333 | 0.185 |  |
| clusters.sep.Mod + same.between | 891.118 | 8.000 | 703.000 | -1766.029 | 1.596 | 0.450 | 0.250 |  |
| front.back.sep.Mod + same.between | 887.841 | 4.000 | 703.000 | -1767.625 | 0.000 | 1.000 | 0.556 |  |
|  |  |  |  |  |  |  |  |  |
| front.back.sep.Mod + same.between |  |  |  |  |  |  |  |  |
|  | Module 1 | Module 2 | betweenModules | unintegrated |  |  |  |  |
| MaxL | 211.130 | 198.343 | 478.369 | 0.000 |  |  |  |  |
| MaxL_p | 0.180 | 0.230 | 0.130 | 0.000 |  |  |  |  |
|  |  |  |  |  |  |  |  |  |
| front.back.same.Mod + same.between |  |  |  |  |  |  |  |  |
|  | withinModules | betweenModules | unintegrated |  |  |  |  |  |
| MaxL | 407.362 | 478.369 | 0.000 |  |  |  |  |  |
| MaxL_p | 0.210 | 0.130 | 0.000 |  |  |  |  |  |
|  |  |  |  |  |  |  |  |  |
| clusters.sep.Mod + same.between |  |  |  |  |  |  |  |  |
|  | Module 1 | Module 2 | Module 3 | Module 6 | Module 4 | Module 5 | betweenModules | unintegrated |
| MaxL | 34.621 | 4.124 | 37.077 | 56.972 | 3.934 | 14.687 | 739.703 | 0.000 |
| MaxL_p | 0.170 | 0.250 | 0.160 | 0.200 | 0.240 | 0.450 | 0.160 | 0.000 |
|  |  |  |  |  |  |  |  |  |
| *Hydrurga leptonyx* |  |  |  |  |  |  |  |  |
| **Before corrections:** |  |  |  |  |  |  |  |  |
|  | MaxL | K | n | AICc | dAICc | Model_L | Post_Pob |  |
| No.modules.default | 1155.882 | 2.000 | 703.000 | -2307.747 | 52.983 | 0.000 | 0.000 |  |
| ossification.sep.Mod + same.between | 1160.558 | 4.000 | 703.000 | -2313.059 | 47.671 | 0.000 | 0.000 |  |
| ossification.same.Mod + same.between | 1159.655 | 3.000 | 703.000 | -2313.276 | 47.454 | 0.000 | 0.000 |  |
| Tissue.origin.same.Mod + same.between | 1162.019 | 3.000 | 703.000 | -2318.003 | 42.726 | 0.000 | 0.000 |  |
| Tissue.origin.sep.Mod + same.between | 1163.274 | 4.000 | 703.000 | -2318.491 | 42.239 | 0.000 | 0.000 |  |
| Function.sep.Mod + same.between | 1169.567 | 6.000 | 703.000 | -2327.012 | 33.718 | 0.000 | 0.000 |  |
| Function.same.Mod + same.between | 1169.412 | 3.000 | 703.000 | -2332.789 | 27.940 | 0.000 | 0.000 |  |
| clusters.same.Mod + same.between | 1177.230 | 3.000 | 703.000 | -2348.426 | 12.304 | 0.002 | 0.001 |  |
| Function.sep.Mod + sep.between | 1185.601 | 11.000 | 703.000 | -2348.820 | 11.910 | 0.003 | 0.001 |  |
| clusters.sep.Mod + same.between | 1183.821 | 8.000 | 703.000 | -2351.435 | 9.294 | 0.010 | 0.005 |  |
| Function.same.Mod + sep.between | 1185.446 | 8.000 | 703.000 | -2354.685 | 6.045 | 0.049 | 0.027 |  |
| clusters.same.Mod + sep.between | 1195.309 | 17.000 | 703.000 | -2355.724 | 5.006 | 0.082 | 0.045 |  |
| clusters.sep.Mod + sep.between | 1201.900 | 22.000 | 703.000 | -2358.312 | 2.418 | 0.299 | 0.164 |  |
| front.back.sep.Mod + same.between | 1183.427 | 4.000 | 703.000 | -2358.797 | 1.933 | 0.380 | 0.209 |  |
| front.back.same.Mod + same.between | 1183.382 | 3.000 | 703.000 | -2360.730 | 0.000 | 1.000 | 0.548 |  |
|  |  |  |  |  |  |  |  |  |
| front.back.sep.Mod + same.between |  |  |  |  |  |  |  |  |
|  | Module 1 | Module 2 | betweenModules | unintegrated |  |  |  |  |
| MaxL | 255.575 | 261.037 | 666.815 | 0.000 |  |  |  |  |
| MaxL_p | 0.170 | 0.180 | 0.100 | 0.000 |  |  |  |  |
|  |  |  |  |  |  |  |  |  |
| front.back.same.Mod + same.between |  |  |  |  |  |  |  |  |
|  | withinModules | betweenModules | unintegrated |  |  |  |  |  |
| MaxL | 516.567 | 666.815 | 0.000 |  |  |  |  |  |
| MaxL_p | 0.180 | 0.100 | 0.000 |  |  |  |  |  |
|  |  |  |  |  |  |  |  |  |
| clusters.sep.Mod + sep.between |  |  |  |  |  |  |  |  |
|  | Module 1 | Module 2 | Module 3 | Module 6 | Module 4 | Module 5 | 1 to 2 | 1 to 3 |
| MaxL | 41.214 | 4.888 | 32.560 | 74.330 | 2.079 | 12.632 | 36.433 | 111.121 |
| MaxL_p | 0.210 | 0.240 | 0.200 | 0.170 | 0.340 | 0.290 | 0.210 | 0.150 |
|  | 1 to 6 | 1 to 4 | 1 to 5 | 2 to 3 | 2 to 6 | 2 to 4 | 2 to 5 | 3 to 6 |
|  | 150.714 | 43.553 | 92.446 | 35.223 | 54.865 | 15.639 | 33.791 | 145.917 |
|  | 0.090 | 0.120 | 0.080 | 0.120 | 0.110 | 0.170 | 0.110 | 0.100 |
|  | 3 to 4 | 3 to 5 | 6 to 4 | 6 to 5 | 4 to 5 | unintegrated |  |  |
|  | 42.816 | 90.105 | 51.301 | 99.078 | 31.195 | 0.000 |  |  |
|  | 0.140 | 0.100 | 0.170 | 0.160 | 0.150 | 0.000 |  |  |
|  |  |  |  |  |  |  |  |  |
| **After corrections:** |  |  |  |  |  |  |  |  |
|  | MaxL | K | n | AICc | dAICc | Model_L | Post_Pob |  |
| No.modules.default | 1158.704 | 2.000 | 703.000 | -2313.391 | 60.409 | 0.000 | 0.000 |  |
| ossification.sep.Mod + same.between | 1163.509 | 4.000 | 703.000 | -2318.960 | 54.840 | 0.000 | 0.000 |  |
| ossification.same.Mod + same.between | 1162.674 | 3.000 | 703.000 | -2319.313 | 54.487 | 0.000 | 0.000 |  |
| Tissue.origin.sep.Mod + same.between | 1166.717 | 4.000 | 703.000 | -2325.378 | 48.422 | 0.000 | 0.000 |  |
| Tissue.origin.same.Mod + same.between | 1166.107 | 3.000 | 703.000 | -2326.179 | 47.621 | 0.000 | 0.000 |  |
| Function.sep.Mod + same.between | 1171.861 | 6.000 | 703.000 | -2331.601 | 42.199 | 0.000 | 0.000 |  |
| Function.same.Mod + same.between | 1171.371 | 3.000 | 703.000 | -2336.708 | 37.091 | 0.000 | 0.000 |  |
| clusters.same.Mod + same.between | 1180.325 | 3.000 | 703.000 | -2354.616 | 19.184 | 0.000 | 0.000 |  |
| clusters.sep.Mod + same.between | 1185.906 | 8.000 | 703.000 | -2355.605 | 18.195 | 0.000 | 0.000 |  |
| Function.sep.Mod + sep.between | 1194.146 | 11.000 | 703.000 | -2365.910 | 7.890 | 0.019 | 0.008 |  |
| Function.same.Mod + sep.between | 1193.657 | 8.000 | 703.000 | -2371.106 | 2.694 | 0.260 | 0.101 |  |
| front.back.sep.Mod + same.between | 1189.917 | 4.000 | 703.000 | -2371.777 | 2.023 | 0.364 | 0.141 |  |
| clusters.same.Mod + sep.between | 1203.429 | 17.000 | 703.000 | -2371.964 | 1.836 | 0.399 | 0.155 |  |
| clusters.sep.Mod + sep.between | 1209.010 | 22.000 | 703.000 | -2372.531 | 1.269 | 0.530 | 0.206 |  |
| front.back.same.Mod + same.between | 1189.917 | 3.000 | 703.000 | -2373.800 | 0.000 | 1.000 | 0.389 |  |
|  |  |  |  |  |  |  |  |  |
| Function.same.Mod + sep.between |  |  |  |  |  |  |  |  |
|  | 1 to 2 | 1 to 4 | 1 to 3 | 2 to 4 | 2 to 3 | 4 to 3 | withinModules | unintegrated |
| MaxL | 94.493 | 393.649 | 138.373 | 101.417 | 33.320 | 123.063 | 309.342 | 0.000 |
| MaxL_p | 0.180 | 0.090 | 0.130 | 0.100 | 0.150 | 0.190 | 0.180 | 0.000 |
|  |  |  |  |  |  |  |  |  |
| front.back.sep.Mod + same.between |  |  |  |  |  |  |  |  |
|  | Module 1 | Module 2 | betweenModules | unintegrated |  |  |  |  |
| MaxL | 259.259 | 267.445 | 663.213 | 0.000 |  |  |  |  |
| MaxL_p | 0.180 | 0.180 | 0.100 | 0.000 |  |  |  |  |
|  |  |  |  |  |  |  |  |  |
| front.back.same.Mod + same.between |  |  |  |  |  |  |  |  |
|  | withinModules | betweenModules | unintegrated |  |  |  |  |  |
| MaxL | 526.704 | 663.213 | 0.000 |  |  |  |  |  |
| MaxL_p | 0.180 | 0.100 | 0.000 |  |  |  |  |  |
|  |  |  |  |  |  |  |  |  |
| clusters.sep.Mod + sep.between |  |  |  |  |  |  |  |  |
|  | Module 1 | Module 2 | Module 3 | Module 6 | Module 4 | Module 5 | 1 to 2 | 1 to 3 |
| MaxL | 40.694 | 4.733 | 35.090 | 76.590 | 2.669 | 14.823 | 36.822 | 112.462 |
| MaxL_p | 0.220 | 0.250 | 0.210 | 0.170 | 0.330 | 0.280 | 0.210 | 0.160 |
|  | 1 to 6 | 1 to 4 | 1 to 5 | 2 to 3 | 2 to 6 | 2 to 4 | 2 to 5 | 3 to 6 |
|  | 150.752 | 43.256 | 92.209 | 35.604 | 53.680 | 14.098 | 34.022 | 144.927 |
|  | 0.090 | 0.130 | 0.080 | 0.120 | 0.120 | 0.190 | 0.100 | 0.100 |
|  | 3 to 4 | 3 to 5 | 6 to 4 | 6 to 5 | 4 to 5 | unintegrated |  |  |
|  | 44.388 | 91.101 | 50.922 | 98.989 | 31.180 | 0.000 |  |  |
|  | 0.140 | 0.080 | 0.170 | 0.160 | 0.180 | 0.000 |  |  |
|  |  |  |  |  |  |  |  |  |
| *Lobodon carcinophaga* |  |  |  |  |  |  |  |  |
| **Before corrections:** |  |  |  |  |  |  |  |  |
|  | MaxL | K | n | AICc | dAICc | Model_L | Post_Pob |  |
| No.modules.default | 1158.215 | 2.000 | 703.000 | -2312.414 | 45.568 | 0.000 | 0.000 |  |
| ossification.sep.Mod + same.between | 1163.571 | 4.000 | 703.000 | -2319.084 | 38.897 | 0.000 | 0.000 |  |
| ossification.same.Mod + same.between | 1162.568 | 3.000 | 703.000 | -2319.102 | 38.879 | 0.000 | 0.000 |  |
| Tissue.origin.sep.Mod + same.between | 1166.399 | 4.000 | 703.000 | -2324.741 | 33.240 | 0.000 | 0.000 |  |
| Tissue.origin.same.Mod + same.between | 1165.696 | 3.000 | 703.000 | -2325.359 | 32.623 | 0.000 | 0.000 |  |
| Function.sep.Mod + same.between | 1173.038 | 6.000 | 703.000 | -2333.956 | 24.025 | 0.000 | 0.000 |  |
| clusters.same.Mod + same.between | 1171.528 | 3.000 | 703.000 | -2337.022 | 20.959 | 0.000 | 0.000 |  |
| Function.same.Mod + same.between | 1171.653 | 3.000 | 703.000 | -2337.272 | 20.709 | 0.000 | 0.000 |  |
| clusters.sep.Mod + same.between | 1177.959 | 8.000 | 703.000 | -2339.710 | 18.271 | 0.000 | 0.000 |  |
| Function.sep.Mod + sep.between | 1183.919 | 11.000 | 703.000 | -2345.456 | 12.525 | 0.002 | 0.001 |  |
| Function.same.Mod + sep.between | 1182.534 | 8.000 | 703.000 | -2348.860 | 9.121 | 0.010 | 0.005 |  |
| clusters.same.Mod + sep.between | 1194.579 | 17.000 | 703.000 | -2354.264 | 3.717 | 0.156 | 0.074 |  |
| front.back.sep.Mod + same.between | 1182.240 | 4.000 | 703.000 | -2356.423 | 1.558 | 0.459 | 0.217 |  |
| clusters.sep.Mod + sep.between | 1201.009 | 22.000 | 703.000 | -2356.530 | 1.451 | 0.484 | 0.229 |  |
| front.back.same.Mod + same.between | 1182.008 | 3.000 | 703.000 | -2357.981 | 0.000 | 1.000 | 0.474 |  |
|  |  |  |  |  |  |  |  |  |
| front.back.sep.Mod + same.between |  |  |  |  |  |  |  |  |
|  | Module 1 | Module 2 | betweenModules | unintegrated |  |  |  |  |
| MaxL | 261.398 | 263.973 | 656.869 | 0.000 |  |  |  |  |
| MaxL_p | 0.190 | 0.170 | 0.110 | 0.000 |  |  |  |  |
|  |  |  |  |  |  |  |  |  |
| front.back.same.Mod + same.between |  |  |  |  |  |  |  |  |
|  | withinModules | betweenModules | unintegrated |  |  |  |  |  |
| MaxL | 525.138 | 656.869 | 0.000 |  |  |  |  |  |
| MaxL_p | 0.180 | 0.110 | 0.000 |  |  |  |  |  |
|  |  |  |  |  |  |  |  |  |
| clusters.sep.Mod + sep.between |  |  |  |  |  |  |  |  |
|  | Module 1 | Module 2 | Module 3 | Module 6 | Module 4 | Module 5 | 1 to 2 | 1 to 3 |
| MaxL | 34.931 | 3.310 | 42.121 | 72.923 | -1.435 | 13.686 | 41.398 | 107.678 |
| MaxL_p | 0.220 | 0.280 | 0.160 | 0.180 | 0.360 | 0.240 | 0.220 | 0.170 |
|  | 1 to 6 | 1 to 4 | 1 to 5 | 2 to 3 | 2 to 6 | 2 to 4 | 2 to 5 | 3 to 6 |
|  | 150.620 | 41.733 | 87.949 | 36.138 | 51.172 | 15.814 | 35.201 | 142.077 |
|  | 0.100 | 0.160 | 0.100 | 0.170 | 0.150 | 0.160 | 0.090 | 0.130 |
|  | 3 to 4 | 3 to 5 | 6 to 4 | 6 to 5 | 4 to 5 | unintegrated |  |  |
|  | 46.645 | 92.223 | 50.673 | 103.737 | 32.413 | 0.000 |  |  |
|  | 0.080 | 0.090 | 0.190 | 0.140 | 0.160 | 0.000 |  |  |
|  |  |  |  |  |  |  |  |  |
| clusters.same.Mod + sep.between |  |  |  |  |  |  |  |  |
|  | 1 to 2 | 1 to 3 | 1 to 6 | 1 to 4 | 1 to 5 | 2 to 3 | 2 to 6 | 2 to 4 |
| MaxL | 41.398 | 107.678 | 150.620 | 41.733 | 87.949 | 36.138 | 51.172 | 15.814 |
| MaxL_p | 0.220 | 0.170 | 0.100 | 0.160 | 0.100 | 0.170 | 0.150 | 0.160 |
|  | 2 to 5 | 3 to 6 | 3 to 4 | 3 to 5 | 6 to 4 | 6 to 5 | 4 to 5 | withinModules |
|  | 35.201 | 142.077 | 46.645 | 92.223 | 50.673 | 103.737 | 32.413 | 159.106 |
|  | 0.090 | 0.130 | 0.080 | 0.090 | 0.190 | 0.140 | 0.160 | 0.200 |
|  | unintegrated |  |  |  |  |  |  |  |
|  | 0.000 |  |  |  |  |  |  |  |
|  | 0.000 |  |  |  |  |  |  |  |
|  |  |  |  |  |  |  |  |  |
| **After corrections:** |  |  |  |  |  |  |  |  |
|  | MaxL | K | n | AICc | dAICc | Model_L | Post_Pob |  |
| No.modules.default | 1129.515 | 2.000 | 703.000 | -2255.012 | 25.834 | 0.000 | 0.000 |  |
| ossification.same.Mod + same.between | 1132.359 | 3.000 | 703.000 | -2258.683 | 22.163 | 0.000 | 0.000 |  |
| ossification.sep.Mod + same.between | 1133.430 | 4.000 | 703.000 | -2258.803 | 22.043 | 0.000 | 0.000 |  |
| Tissue.origin.sep.Mod + same.between | 1135.082 | 4.000 | 703.000 | -2262.107 | 18.739 | 0.000 | 0.000 |  |
| Tissue.origin.same.Mod + same.between | 1134.293 | 3.000 | 703.000 | -2262.551 | 18.295 | 0.000 | 0.000 |  |
| Function.sep.Mod + same.between | 1137.526 | 6.000 | 703.000 | -2262.932 | 17.914 | 0.000 | 0.000 |  |
| Function.sep.Mod + sep.between | 1144.916 | 11.000 | 703.000 | -2267.450 | 13.396 | 0.001 | 0.001 |  |
| Function.same.Mod + same.between | 1136.876 | 3.000 | 703.000 | -2267.718 | 13.128 | 0.001 | 0.001 |  |
| clusters.sep.Mod + sep.between | 1156.788 | 22.000 | 703.000 | -2268.089 | 12.758 | 0.002 | 0.001 |  |
| clusters.sep.Mod + same.between | 1142.289 | 8.000 | 703.000 | -2268.370 | 12.476 | 0.002 | 0.001 |  |
| clusters.same.Mod + same.between | 1137.508 | 3.000 | 703.000 | -2268.981 | 11.865 | 0.003 | 0.002 |  |
| clusters.same.Mod + sep.between | 1152.007 | 17.000 | 703.000 | -2269.122 | 11.725 | 0.003 | 0.002 |  |
| Function.same.Mod + sep.between | 1144.266 | 8.000 | 703.000 | -2272.325 | 8.522 | 0.014 | 0.010 |  |
| front.back.sep.Mod + same.between | 1143.538 | 4.000 | 703.000 | -2279.018 | 1.828 | 0.401 | 0.281 |  |
| front.back.same.Mod + same.between | 1143.440 | 3.000 | 703.000 | -2280.846 | 0.000 | 1.000 | 0.701 |  |
|  |  |  |  |  |  |  |  |  |
| front.back.sep.Mod + same.between |  |  |  |  |  |  |  |  |
|  | Module 1 | Module 2 | betweenModules | unintegrated |  |  |  |  |
| MaxL | 259.302 | 262.326 | 621.910 | 0.000 |  |  |  |  |
| MaxL_p | 0.180 | 0.170 | 0.120 | 0.000 |  |  |  |  |
|  |  |  |  |  |  |  |  |  |
| front.back.same.Mod + same.between |  |  |  |  |  |  |  |  |
|  | withinModules | betweenModules | unintegrated |  |  |  |  |  |
| MaxL | 521.530 | 621.910 | 0.000 |  |  |  |  |  |
| MaxL_p | 0.180 | 0.120 | 0.000 |  |  |  |  |  |
|  |  |  |  |  |  |  |  |  |
| *Leptonychotes weddellii* |  |  |  |  |  |  |  |  |
| **Before corrections:** |  |  |  |  |  |  |  |  |
|  | MaxL | K | n | AICc | dAICc | Model_L | Post_Pob |  |
| No.modules.default | 1213.956 | 2.000 | 703.000 | -2423.895 | 79.258 | 0.000 | 0.000 |  |
| Function.same.Mod + same.between | 1235.166 | 3.000 | 703.000 | -2464.298 | 38.855 | 0.000 | 0.000 |  |
| Function.sep.Mod + same.between | 1237.166 | 6.000 | 703.000 | -2462.211 | 40.942 | 0.000 | 0.000 |  |
| Function.same.Mod + sep.between | 1248.492 | 8.000 | 703.000 | -2480.776 | 22.377 | 0.000 | 0.000 |  |
| Function.sep.Mod + sep.between | 1250.492 | 11.000 | 703.000 | -2478.601 | 24.552 | 0.000 | 0.000 |  |
| Tissue.origin.same.Mod + same.between | 1222.392 | 3.000 | 703.000 | -2438.749 | 64.404 | 0.000 | 0.000 |  |
| Tissue.origin.sep.Mod + same.between | 1223.712 | 4.000 | 703.000 | -2439.368 | 63.786 | 0.000 | 0.000 |  |
| ossification.same.Mod + same.between | 1218.077 | 3.000 | 703.000 | -2430.120 | 73.033 | 0.000 | 0.000 |  |
| ossification.sep.Mod + same.between | 1220.101 | 4.000 | 703.000 | -2432.145 | 71.008 | 0.000 | 0.000 |  |
| front.back.same.Mod + same.between | 1244.676 | 3.000 | 703.000 | -2483.318 | 19.835 | 0.000 | 0.000 |  |
| front.back.sep.Mod + same.between | 1244.676 | 4.000 | 703.000 | -2481.295 | 21.858 | 0.000 | 0.000 |  |
| clusters.same.Mod + same.between | 1242.060 | 3.000 | 703.000 | -2478.086 | 25.067 | 0.000 | 0.000 |  |
| clusters.sep.Mod + same.between | 1254.147 | 8.000 | 703.000 | -2492.086 | 11.067 | 0.004 | 0.004 |  |
| clusters.same.Mod + sep.between | 1262.234 | 17.000 | 703.000 | -2489.575 | 13.578 | 0.001 | 0.001 |  |
| clusters.sep.Mod + sep.between | 1274.321 | 22.000 | 703.000 | -2503.153 | 0.000 | 1.000 | 0.995 |  |
|  |  |  |  |  |  |  |  |  |
| clusters.sep.Mod + sep.between |  |  |  |  |  |  |  |  |
|  | Module 1 | Module 2 | Module 3 | Module 6 | Module 4 | Module 5 | 1 to 2 | 1 to 3 |
| MaxL | 21.001 | 5.928 | 39.190 | 82.228 | -1.365 | 14.340 | 45.601 | 117.251 |
| MaxL_p | 0.250 | 0.110 | 0.190 | 0.150 | 0.390 | 0.210 | 0.120 | 0.140 |
|  | 1 to 6 | 1 to 4 | 1 to 5 | 2 to 3 | 2 to 6 | 2 to 4 | 2 to 5 | 3 to 6 |
|  | 161.681 | 46.708 | 94.698 | 42.870 | 59.784 | 18.139 | 36.513 | 150.487 |
|  | 0.070 | 0.140 | 0.100 | 0.100 | 0.070 | 0.090 | 0.070 | 0.090 |
|  | 3 to 4 | 3 to 5 | 6 to 4 | 6 to 5 | 4 to 5 | unintegrated |  |  |
|  | 48.901 | 97.041 | 57.447 | 104.078 | 31.801 | 0.000 |  |  |
|  | 0.060 | 0.070 | 0.160 | 0.140 | 0.110 | 0.000 |  |  |
|  |  |  |  |  |  |  |  |  |
| **After corrections:** |  |  |  |  |  |  |  |  |
|  | MaxL | K | n | AICc | dAICc | Model_L | Post_Pob |  |
| No.modules.default | 1212.322 | 2.000 | 703.000 | -2420.627 | 79.177 | 0.000 | 0.000 |  |
| ossification.same.Mod + same.between | 1216.277 | 3.000 | 703.000 | -2426.521 | 73.284 | 0.000 | 0.000 |  |
| ossification.sep.Mod + same.between | 1218.329 | 4.000 | 703.000 | -2428.602 | 71.203 | 0.000 | 0.000 |  |
| Tissue.origin.same.Mod + same.between | 1220.434 | 3.000 | 703.000 | -2434.835 | 64.970 | 0.000 | 0.000 |  |
| Tissue.origin.sep.Mod + same.between | 1221.568 | 4.000 | 703.000 | -2435.078 | 64.727 | 0.000 | 0.000 |  |
| Function.sep.Mod + same.between | 1237.532 | 6.000 | 703.000 | -2462.944 | 36.861 | 0.000 | 0.000 |  |
| Function.same.Mod + same.between | 1234.748 | 3.000 | 703.000 | -2463.462 | 36.343 | 0.000 | 0.000 |  |
| clusters.same.Mod + same.between | 1237.911 | 3.000 | 703.000 | -2469.787 | 30.018 | 0.000 | 0.000 |  |
| Function.sep.Mod + sep.between | 1252.550 | 11.000 | 703.000 | -2482.719 | 17.086 | 0.000 | 0.000 |  |
| front.back.sep.Mod + same.between | 1245.415 | 4.000 | 703.000 | -2482.772 | 17.032 | 0.000 | 0.000 |  |
| Function.same.Mod + sep.between | 1249.766 | 8.000 | 703.000 | -2483.325 | 16.480 | 0.000 | 0.000 |  |
| clusters.same.Mod + sep.between | 1259.808 | 17.000 | 703.000 | -2484.723 | 15.082 | 0.001 | 0.001 |  |
| front.back.same.Mod + same.between | 1245.415 | 3.000 | 703.000 | -2484.795 | 15.009 | 0.001 | 0.001 |  |
| clusters.sep.Mod + same.between | 1250.749 | 8.000 | 703.000 | -2485.291 | 14.514 | 0.001 | 0.001 |  |
| clusters.sep.Mod + sep.between | 1272.647 | 22.000 | 703.000 | -2499.805 | 0.000 | 1.000 | 0.998 |  |
|  |  |  |  |  |  |  |  |  |
|  |  |  |  |  |  |  |  |  |
| clusters.sep.Mod + sep.between |  |  |  |  |  |  |  |  |
|  | Module 1 | Module 2 | Module 3 | Module 6 | Module 4 | Module 5 | 1 to 2 | 1 to 3 |
| MaxL | 22.241 | 5.916 | 38.219 | 82.605 | 0.855 | 14.687 | 45.631 | 113.761 |
| MaxL_p | 0.250 | 0.110 | 0.180 | 0.150 | 0.400 | 0.210 | 0.130 | 0.150 |
|  | 1 to 6 | 1 to 4 | 1 to 5 | 2 to 3 | 2 to 6 | 2 to 4 | 2 to 5 | 3 to 6 |
|  | 161.349 | 46.731 | 94.968 | 43.083 | 59.711 | 17.943 | 36.503 | 150.647 |
|  | 0.070 | 0.140 | 0.100 | 0.100 | 0.070 | 0.100 | 0.070 | 0.090 |
|  | 3 to 4 | 3 to 5 | 6 to 4 | 6 to 5 | 4 to 5 | unintegrated |  |  |
|  | 48.563 | 96.977 | 57.318 | 102.481 | 32.459 | 0.000 |  |  |
|  | 0.060 | 0.070 | 0.170 | 0.140 | 0.120 | 0.000 |  |  |
|  |  |  |  |  |  |  |  |  |
| *Mirounga leonina* |  |  |  |  |  |  |  |  |
| **Before corrections:** |  |  |  |  |  |  |  |  |
|  | MaxL | K | n | AICc | dAICc | Model_L | Post_Pob |  |
| Tissue.origin.same.Mod + same.between | 951.055 | 3.000 | 703.000 | -1896.076 | 40.625 | 0.000 | 0.000 |  |
| ossification.same.Mod + same.between | 951.055 | 3.000 | 703.000 | -1896.076 | 40.625 | 0.000 | 0.000 |  |
| No.modules.default | 951.055 | 2.000 | 703.000 | -1898.093 | 38.607 | 0.000 | 0.000 |  |
| ossification.sep.Mod + same.between | 953.356 | 4.000 | 703.000 | -1898.656 | 38.045 | 0.000 | 0.000 |  |
| Tissue.origin.sep.Mod + same.between | 957.857 | 4.000 | 703.000 | -1907.658 | 29.042 | 0.000 | 0.000 |  |
| clusters.same.Mod + same.between | 959.883 | 3.000 | 703.000 | -1913.732 | 22.968 | 0.000 | 0.000 |  |
| clusters.sep.Mod + same.between | 965.943 | 8.000 | 703.000 | -1915.679 | 21.021 | 0.000 | 0.000 |  |
| front.back.same.Mod + same.between | 961.180 | 3.000 | 703.000 | -1916.326 | 20.374 | 0.000 | 0.000 |  |
| Function.same.Mod + sep.between | 967.401 | 8.000 | 703.000 | -1918.595 | 18.105 | 0.000 | 0.000 |  |
| clusters.same.Mod + sep.between | 977.441 | 17.000 | 703.000 | -1919.989 | 16.711 | 0.000 | 0.000 |  |
| clusters.sep.Mod + sep.between | 983.502 | 22.000 | 703.000 | -1921.515 | 15.185 | 0.001 | 0.000 |  |
| Function.same.Mod + same.between | 965.304 | 3.000 | 703.000 | -1924.574 | 12.126 | 0.002 | 0.002 |  |
| front.back.sep.Mod + same.between | 966.514 | 4.000 | 703.000 | -1924.970 | 11.730 | 0.003 | 0.003 |  |
| Function.sep.Mod + sep.between | 976.507 | 11.000 | 703.000 | -1930.632 | 6.068 | 0.048 | 0.046 |  |
| Function.sep.Mod + same.between | 974.410 | 6.000 | 703.000 | -1936.700 | 0.000 | 1.000 | 0.949 |  |
|  |  |  |  |  |  |  |  |  |
|  |  |  |  |  |  |  |  |  |
| Function.sep.Mod + same.between |  |  |  |  |  |  |  |  |
|  | Module 1 | Module 2 | Module 4 | Module 3 | betweenModules | unintegrated |  |  |
| MaxL | 141.794 | 8.248 | 102.807 | 8.478 | 713.082 | 0.000 |  |  |
| MaxL_p | 0.190 | 0.180 | 0.300 | 0.160 | 0.150 | 0.000 |  |  |
|  |  |  |  |  |  |  |  |  |
| **After corrections:** |  |  |  |  |  |  |  |  |
|  | MaxL | K | n | AICc | dAICc | Model_L | Post_Pob |  |
| Tissue.origin.same.Mod + same.between | 983.141 | 3.000 | 703.000 | -1960.248 | 45.728 | 0.000 | 0.000 |  |
| No.modules.default | 982.548 | 2.000 | 703.000 | -1961.079 | 44.896 | 0.000 | 0.000 |  |
| ossification.same.Mod + same.between | 983.768 | 3.000 | 703.000 | -1961.501 | 44.474 | 0.000 | 0.000 |  |
| ossification.sep.Mod + same.between | 985.766 | 4.000 | 703.000 | -1963.475 | 42.501 | 0.000 | 0.000 |  |
| Function.same.Mod + sep.between | 991.840 | 8.000 | 703.000 | -1967.472 | 38.503 | 0.000 | 0.000 |  |
| clusters.same.Mod + same.between | 988.034 | 3.000 | 703.000 | -1970.033 | 35.942 | 0.000 | 0.000 |  |
| Tissue.origin.sep.Mod + same.between | 989.397 | 4.000 | 703.000 | -1970.738 | 35.238 | 0.000 | 0.000 |  |
| front.back.same.Mod + same.between | 989.276 | 3.000 | 703.000 | -1972.517 | 33.458 | 0.000 | 0.000 |  |
| Function.same.Mod + same.between | 990.025 | 3.000 | 703.000 | -1974.016 | 31.959 | 0.000 | 0.000 |  |
| clusters.sep.Mod + same.between | 996.484 | 8.000 | 703.000 | -1976.760 | 29.215 | 0.000 | 0.000 |  |
| clusters.same.Mod + sep.between | 1010.353 | 17.000 | 703.000 | -1985.812 | 20.163 | 0.000 | 0.000 |  |
| clusters.sep.Mod + sep.between | 1018.803 | 22.000 | 703.000 | -1992.118 | 13.858 | 0.001 | 0.001 |  |
| front.back.sep.Mod + same.between | 1001.589 | 4.000 | 703.000 | -1995.121 | 10.854 | 0.004 | 0.004 |  |
| Function.sep.Mod + sep.between | 1010.863 | 11.000 | 703.000 | -1999.343 | 6.632 | 0.036 | 0.035 |  |
| Function.sep.Mod + same.between | 1009.048 | 6.000 | 703.000 | -2005.975 | 0.000 | 1.000 | 0.960 |  |
|  |  |  |  |  |  |  |  |  |
| Function.sep.Mod + same.between |  |  |  |  |  |  |  |  |
|  | Module 1 | Module 2 | Module 4 | Module 3 | betweenModules | unintegrated |  |  |
| MaxL | 156.226 | 7.437 | 102.419 | 10.474 | 732.491 | 0.000 |  |  |
| MaxL_p | 0.130 | 0.210 | 0.290 | 0.200 | 0.150 | 0.000 |  |  |
|  |  |  |  |  |  |  |  |  |
| *Pagophilus groenlandicus* |  |  |  |  |  |  |  |  |
| **Before corrections:** |  |  |  |  |  |  |  |  |
|  | MaxL | K | n | AICc | dAICc | Model_L | Post_Pob |  |
| No.modules.default | 1158.766 | 2.000 | 703.000 | -2313.514 | 27.221 | 0.000 | 0.000 |  |
| ossification.sep.Mod + same.between | 1160.929 | 4.000 | 703.000 | -2313.801 | 26.934 | 0.000 | 0.000 |  |
| ossification.same.Mod + same.between | 1160.122 | 3.000 | 703.000 | -2314.210 | 26.525 | 0.000 | 0.000 |  |
| Tissue.origin.sep.Mod + same.between | 1163.429 | 4.000 | 703.000 | -2318.800 | 21.935 | 0.000 | 0.000 |  |
| Tissue.origin.same.Mod + same.between | 1163.396 | 3.000 | 703.000 | -2320.758 | 19.978 | 0.000 | 0.000 |  |
| Function.same.Mod + sep.between | 1174.556 | 8.000 | 703.000 | -2332.904 | 7.831 | 0.020 | 0.007 |  |
| clusters.same.Mod + sep.between | 1184.355 | 17.000 | 703.000 | -2333.817 | 6.918 | 0.031 | 0.012 |  |
| Function.same.Mod + same.between | 1170.251 | 3.000 | 703.000 | -2334.467 | 6.268 | 0.044 | 0.016 |  |
| clusters.sep.Mod + sep.between | 1190.851 | 22.000 | 703.000 | -2336.213 | 4.522 | 0.104 | 0.038 |  |
| front.back.same.Mod + same.between | 1171.408 | 3.000 | 703.000 | -2336.782 | 3.953 | 0.139 | 0.051 |  |
| Function.sep.Mod + sep.between | 1180.119 | 11.000 | 703.000 | -2337.856 | 2.879 | 0.237 | 0.087 |  |
| clusters.same.Mod + same.between | 1171.976 | 3.000 | 703.000 | -2337.918 | 2.817 | 0.244 | 0.090 |  |
| front.back.sep.Mod + same.between | 1173.395 | 4.000 | 703.000 | -2338.732 | 2.003 | 0.367 | 0.135 |  |
| Function.sep.Mod + same.between | 1175.814 | 6.000 | 703.000 | -2339.507 | 1.228 | 0.541 | 0.198 |  |
| clusters.sep.Mod + same.between | 1178.471 | 8.000 | 703.000 | -2340.735 | 0.000 | 1.000 | 0.367 |  |
|  |  |  |  |  |  |  |  |  |
|  |  |  |  |  |  |  |  |  |
| Function.sep.Mod + sep.between |  |  |  |  |  |  |  |  |
|  | Module 1 | Module 2 | Module 4 | Module 3 | 1 to 2 | 1 to 4 | 1 to 3 |  |
| MaxL | 147.350 | 7.590 | 147.560 | 11.192 | 103.792 | 377.715 | 131.975 |  |
| MaxL_p | 0.190 | 0.140 | 0.120 | 0.210 | 0.120 | 0.090 | 0.100 |  |
|  | 2 to 4 | 2 to 3 | 4 to 3 | unintegrated |  |  |  |  |
|  | 95.751 | 37.352 | 119.841 | 0.000 |  |  |  |  |
|  | 0.110 | 0.060 | 0.130 | 0.000 |  |  |  |  |
|  |  |  |  |  |  |  |  |  |
| Function.sep.Mod + same.between |  |  |  |  |  |  |  |  |
|  | Module 1 | Module 2 | Module 4 | Module 3 | betweenModules | unintegrated |  |  |
| MaxL | 147.350 | 7.590 | 147.560 | 11.192 | 862.121 | 0.000 |  |  |
| MaxL_p | 0.190 | 0.140 | 0.120 | 0.210 | 0.100 | 0.000 |  |  |
|  |  |  |  |  |  |  |  |  |
| front.back.sep.Mod + same.between |  |  |  |  |  |  |  |  |
|  | Module 1 | Module 2 | betweenModules | unintegrated |  |  |  |  |
| MaxL | 255.065 | 276.860 | 641.469 | 0.000 |  |  |  |  |
| MaxL_p | 0.160 | 0.130 | 0.090 | 0.000 |  |  |  |  |
|  |  |  |  |  |  |  |  |  |
| front.back.same.Mod + same.between |  |  |  |  |  |  |  |  |
|  | withinModules | betweenModules | unintegrated |  |  |  |  |  |
| MaxL | 529.939 | 641.469 | 0.000 |  |  |  |  |  |
| MaxL_p | 0.150 | 0.090 | 0.000 |  |  |  |  |  |
|  |  |  |  |  |  |  |  |  |
| **After corrections:** |  |  |  |  |  |  |  |  |
|  | MaxL | K | n | AICc | dAICc | Model_L | Post_Pob |  |
| No.modules.default | 1162.010 | 2.000 | 703.000 | -2320.003 | 25.796 | 0.000 | 0.000 |  |
| ossification.same.Mod + same.between | 1163.528 | 3.000 | 703.000 | -2321.021 | 24.778 | 0.000 | 0.000 |  |
| ossification.sep.Mod + same.between | 1164.677 | 4.000 | 703.000 | -2321.297 | 24.501 | 0.000 | 0.000 |  |
| Tissue.origin.sep.Mod + same.between | 1166.990 | 4.000 | 703.000 | -2325.923 | 19.876 | 0.000 | 0.000 |  |
| Tissue.origin.same.Mod + same.between | 1166.945 | 3.000 | 703.000 | -2327.855 | 17.943 | 0.000 | 0.000 |  |
| clusters.same.Mod + sep.between | 1185.691 | 17.000 | 703.000 | -2336.489 | 9.309 | 0.010 | 0.004 |  |
| Function.same.Mod + sep.between | 1177.021 | 8.000 | 703.000 | -2337.834 | 7.964 | 0.019 | 0.008 |  |
| clusters.sep.Mod + sep.between | 1191.918 | 22.000 | 703.000 | -2338.347 | 7.451 | 0.024 | 0.010 |  |
| Function.same.Mod + same.between | 1172.958 | 3.000 | 703.000 | -2339.882 | 5.916 | 0.052 | 0.022 |  |
| front.back.same.Mod + same.between | 1173.621 | 3.000 | 703.000 | -2341.208 | 4.591 | 0.101 | 0.044 |  |
| Function.sep.Mod + sep.between | 1182.211 | 11.000 | 703.000 | -2342.041 | 3.758 | 0.153 | 0.066 |  |
| front.back.sep.Mod + same.between | 1175.250 | 4.000 | 703.000 | -2342.444 | 3.355 | 0.187 | 0.081 |  |
| clusters.same.Mod + same.between | 1174.777 | 3.000 | 703.000 | -2343.519 | 2.279 | 0.320 | 0.139 |  |
| Function.sep.Mod + same.between | 1178.149 | 6.000 | 703.000 | -2344.177 | 1.621 | 0.445 | 0.193 |  |
| clusters.sep.Mod + same.between | 1181.003 | 8.000 | 703.000 | -2345.798 | 0.000 | 1.000 | 0.433 |  |
|  |  |  |  |  |  |  |  |  |
|  |  |  |  |  |  |  |  |  |
| Function.sep.Mod + sep.between |  |  |  |  |  |  |  |  |
|  | Module 1 | Module 2 | Module 4 | Module 3 | 1 to 2 | 1 to 4 | 1 to 3 |  |
| MaxL | 149.818 | 7.679 | 146.943 | 11.327 | 102.426 | 378.379 | 131.866 |  |
| MaxL_p | 0.180 | 0.150 | 0.120 | 0.220 | 0.120 | 0.090 | 0.100 |  |
|  | 2 to 4 | 2 to 3 | 4 to 3 | unintegrated |  |  |  |  |
|  | 96.525 | 37.351 | 119.899 | 0.000 |  |  |  |  |
|  | 0.110 | 0.050 | 0.130 | 0.000 |  |  |  |  |
|  |  |  |  |  |  |  |  |  |
| Function.sep.Mod + same.between |  |  |  |  |  |  |  |  |
|  | Module 1 | Module 2 | Module 4 | Module 3 | betweenModules | unintegrated |  |  |
| MaxL | 149.818 | 7.679 | 146.943 | 11.327 | 862.383 | 0.000 |  |  |
| MaxL_p | 0.180 | 0.150 | 0.120 | 0.220 | 0.100 | 0.000 |  |  |
|  |  |  |  |  |  |  |  |  |
| front.back.sep.Mod + same.between |  |  |  |  |  |  |  |  |
|  | Module 1 | Module 2 | betweenModules | unintegrated |  |  |  |  |
| MaxL | 256.349 | 276.134 | 642.767 | 0.000 |  |  |  |  |
| MaxL_p | 0.160 | 0.130 | 0.090 | 0.000 |  |  |  |  |
|  |  |  |  |  |  |  |  |  |
| clusters.same.Mod + same.between |  |  |  |  |  |  |  |  |
|  | withinModules | betweenModules | unintegrated |  |  |  |  |  |
| MaxL | 163.275 | 1011.502 | 0.000 |  |  |  |  |  |
| MaxL_p | 0.180 | 0.100 | 0.000 |  |  |  |  |  |
|  |  |  |  |  |  |  |  |  |
| clusters.sep.Mod + same.between |  |  |  |  |  |  |  |  |
|  | Module 1 | Module 2 | Module 3 | Module 6 | Module 4 | Module 5 | betweenModules |  |
| MaxL | 33.408 | 5.214 | 41.129 | 74.781 | 0.178 | 14.791 | 1011.502 |  |
| MaxL_p | 0.250 | 0.070 | 0.160 | 0.160 | 0.300 | 0.150 | 0.100 |  |
|  | unintegrated |  |  |  |  |  |  |  |
|  | 0.000 |  |  |  |  |  |  |  |
|  | 0.000 |  |  |  |  |  |  |  |
|  |  |  |  |  |  |  |  |  |
| *Phoca vitulina* |  |  |  |  |  |  |  |  |
| **Before corrections:** |  |  |  |  |  |  |  |  |
|  | MaxL | K | n | AICc | dAICc | Model_L | Post_Pob |  |
| ossification.same.Mod + same.between | 1043.989 | 3.000 | 703.000 | -2081.943 | 27.104 | 0.000 | 0.000 |  |
| Tissue.origin.same.Mod + same.between | 1044.060 | 3.000 | 703.000 | -2082.085 | 26.962 | 0.000 | 0.000 |  |
| No.modules.default | 1043.989 | 2.000 | 703.000 | -2083.960 | 25.087 | 0.000 | 0.000 |  |
| ossification.sep.Mod + same.between | 1046.113 | 4.000 | 703.000 | -2084.168 | 24.879 | 0.000 | 0.000 |  |
| Tissue.origin.sep.Mod + same.between | 1046.821 | 4.000 | 703.000 | -2085.584 | 23.463 | 0.000 | 0.000 |  |
| clusters.same.Mod + sep.between | 1062.170 | 17.000 | 703.000 | -2089.447 | 19.600 | 0.000 | 0.000 |  |
| clusters.same.Mod + same.between | 1049.747 | 3.000 | 703.000 | -2093.461 | 15.587 | 0.000 | 0.000 |  |
| clusters.sep.Mod + sep.between | 1072.447 | 22.000 | 703.000 | -2099.405 | 9.642 | 0.008 | 0.005 |  |
| front.back.same.Mod + same.between | 1053.266 | 3.000 | 703.000 | -2100.499 | 8.548 | 0.014 | 0.008 |  |
| Function.same.Mod + sep.between | 1059.447 | 8.000 | 703.000 | -2102.687 | 6.360 | 0.042 | 0.024 |  |
| Function.same.Mod + same.between | 1054.526 | 3.000 | 703.000 | -2103.017 | 6.030 | 0.049 | 0.028 |  |
| clusters.sep.Mod + same.between | 1060.024 | 8.000 | 703.000 | -2103.840 | 5.207 | 0.074 | 0.042 |  |
| Function.sep.Mod + sep.between | 1064.353 | 11.000 | 703.000 | -2106.323 | 2.724 | 0.256 | 0.146 |  |
| Function.sep.Mod + same.between | 1059.431 | 6.000 | 703.000 | -2106.741 | 2.306 | 0.316 | 0.179 |  |
| front.back.sep.Mod + same.between | 1058.552 | 4.000 | 703.000 | -2109.047 | 0.000 | 1.000 | 0.569 |  |
|  |  |  |  |  |  |  |  |  |
| Function.sep.Mod + sep.between |  |  |  |  |  |  |  |  |
|  | Module 1 | Module 2 | Module 4 | Module 3 | 1 to 2 | 1 to 4 | 1 to 3 |  |
| MaxL | 119.971 | 2.549 | 111.972 | 6.305 | 101.807 | 356.098 | 133.133 |  |
| MaxL_p | 0.150 | 0.200 | 0.210 | 0.230 | 0.120 | 0.120 | 0.090 |  |
|  | 2 to 4 | 2 to 3 | 4 to 3 | unintegrated |  |  |  |  |
|  | 92.426 | 30.088 | 110.004 | 0.000 |  |  |  |  |
|  | 0.130 | 0.160 | 0.160 | 0.000 |  |  |  |  |
|  |  |  |  |  |  |  |  |  |
| Function.sep.Mod + same.between |  |  |  |  |  |  |  |  |
|  | Module 1 | Module 2 | Module 4 | Module 3 | betweenModules | unintegrated |  |  |
| MaxL | 119.971 | 2.549 | 111.972 | 6.305 | 818.634 | 0.000 |  |  |
| MaxL_p | 0.150 | 0.200 | 0.210 | 0.230 | 0.120 | 0.000 |  |  |
|  |  |  |  |  |  |  |  |  |
| front.back.sep.Mod + same.between |  |  |  |  |  |  |  |  |
|  | Module 1 | Module 2 | betweenModules | unintegrated |  |  |  |  |
| MaxL | 223.147 | 225.790 | 609.615 | 0.000 |  |  |  |  |
| MaxL_p | 0.140 | 0.190 | 0.120 | 0.000 |  |  |  |  |
|  |  |  |  |  |  |  |  |  |
| **After corrections:** |  |  |  |  |  |  |  |  |
| Females |  |  |  |  |  |  |  |  |
| clusters.sep.Mod + sep.between | 720.515 | 22.000 | 703.000 | -1395.542 | 20.791 | 0.000 | 0.000 |  |
| ossification.same.Mod + same.between | 701.267 | 3.000 | 703.000 | -1396.500 | 19.833 | 0.000 | 0.000 |  |
| Tissue.origin.same.Mod + same.between | 701.457 | 3.000 | 703.000 | -1396.880 | 19.452 | 0.000 | 0.000 |  |
| clusters.same.Mod + sep.between | 715.899 | 17.000 | 703.000 | -1396.904 | 19.428 | 0.000 | 0.000 |  |
| No.modules.default | 701.166 | 2.000 | 703.000 | -1398.315 | 18.017 | 0.000 | 0.000 |  |
| ossification.sep.Mod + same.between | 704.245 | 4.000 | 703.000 | -1400.432 | 15.900 | 0.000 | 0.000 |  |
| Tissue.origin.sep.Mod + same.between | 704.844 | 4.000 | 703.000 | -1401.630 | 14.702 | 0.001 | 0.000 |  |
| Function.same.Mod + sep.between | 709.077 | 8.000 | 703.000 | -1401.946 | 14.386 | 0.001 | 0.000 |  |
| clusters.sep.Mod + same.between | 709.974 | 8.000 | 703.000 | -1403.740 | 12.592 | 0.002 | 0.001 |  |
| clusters.same.Mod + same.between | 705.357 | 3.000 | 703.000 | -1404.681 | 11.652 | 0.003 | 0.002 |  |
| Function.same.Mod + same.between | 706.038 | 3.000 | 703.000 | -1406.041 | 10.291 | 0.006 | 0.004 |  |
| Function.sep.Mod + sep.between | 716.397 | 11.000 | 703.000 | -1410.413 | 5.919 | 0.052 | 0.033 |  |
| front.back.same.Mod + same.between | 708.811 | 3.000 | 703.000 | -1411.589 | 4.744 | 0.093 | 0.059 |  |
| Function.sep.Mod + same.between | 713.358 | 6.000 | 703.000 | -1414.596 | 1.736 | 0.420 | 0.266 |  |
| front.back.sep.Mod + same.between | 712.195 | 4.000 | 703.000 | -1416.332 | 0.000 | 1.000 | 0.634 |  |
|  |  |  |  |  |  |  |  |  |
|  |  |  |  |  |  |  |  |  |
| Function.sep.Mod + same.between |  |  |  |  |  |  |  |  |
|  | Module 1 | Module 2 | Module 4 | Module 3 | betweenModules | unintegrated |  |  |
| MaxL | 108.533 | 4.154 | 71.514 | 9.203 | 519.955 | 0.000 |  |  |
| MaxL_p | 0.200 | 0.340 | 0.350 | 0.340 | 0.200 | 0.000 |  |  |
|  |  |  |  |  |  |  |  |  |
| front.back.sep.Mod + same.between |  |  |  |  |  |  |  |  |
|  | Module 1 | Module 2 | betweenModules | unintegrated |  |  |  |  |
| MaxL | 169.709 | 146.126 | 396.360 | 0.000 |  |  |  |  |
| MaxL_p | 0.230 | 0.310 | 0.180 | 0.000 |  |  |  |  |
|  |  |  |  |  |  |  |  |  |
| front.back.same.Mod + same.between |  |  |  |  |  |  |  |  |
|  | withinModules | betweenModules | unintegrated |  |  |  |  |  |
| MaxL | 312.452 | 396.360 | 0.000 |  |  |  |  |  |
| MaxL_p | 0.270 | 0.180 | 0.000 |  |  |  |  |  |
|  |  |  |  |  |  |  |  |  |
| Males |  |  |  |  |  |  |  |  |
|  | MaxL | K | n | AICc | dAICc | Model_L | Post_Pob |  |
| clusters.sep.Mod + sep.between | 741.389 | 22.000 | 703.000 | -1437.290 | 16.663 | 0.000 | 0.000 |  |
| Tissue.origin.sep.Mod + same.between | 724.526 | 4.000 | 703.000 | -1440.995 | 12.958 | 0.002 | 0.001 |  |
| Tissue.origin.same.Mod + same.between | 723.560 | 3.000 | 703.000 | -1441.085 | 12.868 | 0.002 | 0.001 |  |
| ossification.same.Mod + same.between | 723.692 | 3.000 | 703.000 | -1441.349 | 12.604 | 0.002 | 0.002 |  |
| Function.same.Mod + sep.between | 728.922 | 8.000 | 703.000 | -1441.636 | 12.317 | 0.002 | 0.002 |  |
| Function.sep.Mod + sep.between | 732.473 | 11.000 | 703.000 | -1442.563 | 11.390 | 0.003 | 0.003 |  |
| No.modules.default | 723.560 | 2.000 | 703.000 | -1443.102 | 10.851 | 0.004 | 0.004 |  |
| clusters.same.Mod + sep.between | 739.219 | 17.000 | 703.000 | -1443.545 | 10.408 | 0.005 | 0.004 |  |
| Function.same.Mod + same.between | 724.909 | 3.000 | 703.000 | -1443.785 | 10.168 | 0.006 | 0.005 |  |
| Function.sep.Mod + same.between | 728.460 | 6.000 | 703.000 | -1444.800 | 9.153 | 0.010 | 0.008 |  |
| ossification.sep.Mod + same.between | 727.153 | 4.000 | 703.000 | -1446.249 | 7.704 | 0.021 | 0.017 |  |
| front.back.sep.Mod + same.between | 727.963 | 4.000 | 703.000 | -1447.869 | 6.084 | 0.048 | 0.039 |  |
| clusters.sep.Mod + same.between | 732.163 | 8.000 | 703.000 | -1448.119 | 5.834 | 0.054 | 0.044 |  |
| front.back.same.Mod + same.between | 727.202 | 3.000 | 703.000 | -1448.370 | 5.583 | 0.061 | 0.050 |  |
| clusters.same.Mod + same.between | 729.994 | 3.000 | 703.000 | -1453.953 | 0.000 | 1.000 | 0.819 |  |
|  |  |  |  |  |  |  |  |  |
|  |  |  |  |  |  |  |  |  |
| front.back.same.Mod + same.between |  |  |  |  |  |  |  |  |
|  | withinModules | betweenModules | unintegrated |  |  |  |  |  |
| MaxL | 341.671 | 385.531 | 0.000 |  |  |  |  |  |
| MaxL_p | 0.230 | 0.170 | 0.000 |  |  |  |  |  |
|  |  |  |  |  |  |  |  |  |
| clusters.same.Mod + same.between |  |  |  |  |  |  |  |  |
|  | withinModules | betweenModules | unintegrated |  |  |  |  |  |
| MaxL | 113.249 | 616.745 | 0.000 |  |  |  |  |  |
| MaxL_p | 0.280 | 0.180 | 0.000 |  |  |  |  |  |
