# Supplementary material for: Shifts in cranial integration associated with ecological specialization in pinnipeds (Mammalia, Carnivora)
Source: R Soc Open Sci. 2019 Mar 27;6(3):190201. doi: 10.1098/rsos.190201 (PMC6458409; doi:10.1098/rsos.190201)
Supplement: Reviewer comments [file rsos190201_review_history.pdf]

THE ROYAL SOCIETY
PUBLISHING

ROYAL SOCIETY
OPEN SCIENCE

Shifts in cranial integration associated with ecological specialization in pinnipeds (Mammalia, Carnivora)

Marcela Randau, Daniela Sanfelice and Anjali Goswami

Article citation details
R. Soc. open sci. **6**: 190201.
http://dx.doi.org/10.1098/rsos.190201

Review timeline

Original submission:	5 February 2019
Final acceptance:	15 February 2019

Note: Reports are unedited and appear as submitted by the referee. The review history appears in chronological order.

Note: This manuscript was transferred from another Royal Society journal with peer review.

Review History

Decision letter (RSOS-190201.R0)

15-Feb-2019

Dear Dr Randau:

It is a pleasure to accept your manuscript entitled "Shifts in cranial integration associated with ecological specialisation in pinnipeds (Mammalia, Carnivora)" in its current form for publication in Royal Society Open Science. The comments of the reviewer(s) who reviewed your manuscript are included at the foot of this letter.

Thank you for your fine contribution. On behalf of the Editors of Royal Society Open Science, we look forward to your continued contributions to the Journal.

Kind regards,
Andrew Dunn
Senior Publishing Editor
Royal Society Open Science
openscience@royalsociety.org

Reports © 2019 The Reviewers; Decision Letters © 2019 The Reviewers and Editors; Responses © 2019 The Reviewers, Editors and Authors. Published by the Royal Society under the terms of the Creative Commons Attribution License http://creativecommons.org/licenses/by/4.0/, which permits unrestricted use, provided the original author and source are credited

on behalf of Professor Emily Standen (Associate Editor) and Dr Kevin Padian (Subject Editor).

Associate Editor Professor Emily Standen Comments to Author:

Associate Editor
Comments to the Author:
Dear Marcela Randau,

We are happy to receive your manuscripts entitled "Shifts in cranial integration associated with ecological specialization in pinnipeds (Mammalia, Carnivora). The reviews that accompany your paper are very positive and if you can address their comments we are happy to consider your manuscript for publication.

I look forward to seeing your revised ms.

Sincerely,

Emily Standen

Follow Royal Society Publishing on Twitter: @RSocPublishing
Follow Royal Society Publishing on Facebook:
https://www.facebook.com/RoyalSocietyPublishing.FanPage/
Read Royal Society Publishing's blog: https://blogs.royalsociety.org/publishing/
